# Holding in the stream: convergent evolution of suckermouth structures in Loricariidae (Siluriformes)

**DOI:** 10.1186/s12983-023-00516-w

**Published:** 2023-12-01

**Authors:** Wencke Krings, Daniel Konn-Vetterlein, Bernhard Hausdorf, Stanislav N. Gorb

**Affiliations:** 1 Department of Functional Morphology and Biomechanics, Zoological Institute, Kiel University, Am Botanischen Garten 1–9, 24118 Kiel, Germany; 2 Department of Cariology, Endodontology and Periodontology, University of Leipzig, Liebigstraße 12, 04103 Leipzig, Germany; 3 Department of Mammalogy and Palaeoanthropology, Leibniz Institute for the Analysis of Biodiversity Change, Martin-Luther-King-Platz 3, 20146 Hamburg, Germany; 4 Department of Electron Microscopy, Institute of Cell and Systems Biology of Animals, University of Hamburg, Martin-Luther-King-Platz 3, 20146 Hamburg, Germany; 5 Department of Malacology, Leibniz Institute for the Analysis of Biodiversity Change, Martin-Luther-King-Platz 3, 20146 Hamburg, Germany

**Keywords:** Adhesion, Friction, Attachment, Interlocking, Suction, Fish, Unculi, Oral disc

## Abstract

Suckermouth armoured catfish (Loricariidae) are a highly speciose and diverse freshwater fish family, which bear upper and lower lips forming an oral disc. Its hierarchical organisation allows the attachment to various natural surfaces. The discs can possess papillae of different shapes, which are supplemented, in many taxa, by small horny projections, i.e. unculi. Although these attachment structures and their working mechanisms, which include adhesion and interlocking, are rather well investigated in some selected species, the loricariid oral disc is unfortunately understudied in the majority of species, especially with regard to comparative aspects of the diverse oral structures and their relationship to the ecology of different species. In the present paper, we investigated the papilla and unculi morphologies in 67 loricariid species, which inhabit different currents and substrates. We determined four papilla types and eight unculi types differing by forms and sizes. Ancestral state reconstructions strongly suggest convergent evolution of traits. There is no obvious correlation between habitat shifts and the evolution of specific character states. From handling the structures and from drying artefacts we could infer some information about their material properties. This, together with their shape, enabled us to carefully propose hypotheses about mechanisms of interactions of oral disc structures with natural substrates typical for respective fish species.

**Supplementary Information:**

The online version contains supplementary material available at 10.1186/s12983-023-00516-w.

## Background

The suckermouth armored catfishes (Loricariidae, Siluriformes, Teleostei) are among the most interesting animal groups, because this family is, with about 1000 species [1], highly specious as a result of an evolutionary radiation, which included convergent evolution of traits [2, 3]. These neotropical freshwater fish are particularly diverse with regard to body colorations and shapes, reflecting their high degree of specialisation to different habitats and speciation.

Loricariidae are characterized by a depressed body shape covered by bony plates and the modification of the mouth to an oral sucker disc, which is used, besides for feeding, for the attachment to various substrates [2, 4–7]. In specialized species, it also facilitates climbing [8]. This oral disc, which can only be found in three more catfish families (Astroblepidae, some genera in Mochokidae and in Sisoridae), is an adaptation to fast flowing water bodies and is used to generate negative pressure to allow surface attachment [6]. In fish taxa, such as Balitoridae, Gobiesocidae, Cyclopteridae, Liparidae, Echeneidae, and Cyprinidae, attachment structures and attachment mechanisms have been previously investigated [9–23].

In general, attachment mechanisms are omnipresent in animals, including insects [24–32], molluscs [33, 34], reptiles [35, 36], amphibians [37, 38], mammals [39, 40] and, as mentioned above, fishes [17, 41, 42].

These mechanisms are diverse and can include interlocking structures, such as hooks, locks, clamps or spacers [26], wet and dry adhesion [43, 44], and/or suction cups [15, 17]. Depending on the attachment system, physical effects as friction, mechanical interlocking, muscular force, viscous forces, chemical bonding, capillary effects, van der Waals forces, and electrostatic forces are involved and can lead to permanent, transitory and temporary attachment time to the substrate [26, 45, 46].

With regard to the aquatic environment, two main attachment strategies, bioadhesive secretion or suction attachment, seem to be present as adaptation to the specific physical conditions [see reviews 46, 47]. Glue-like bioadhesive secretions include complex mixtures of proteins, lipids and sugars and can be found in echnioderms, mussels, or barnacles. Suction attachment involves muscular contraction to generate pressure differences and can be found in cephalopods, some insect taxa, and fish. In some species, both mechanisms can be found, as in lottiid limpets or fish. Depending on the taxa, attachment is achieved by multiple points of interaction, as in Echniodermata or Cephalopoda, or by one single attachment point, as in limpets or fish [see review 47].

In fish, the attachment structure, i.e., the suction disc, represents a chamber of subatmospheric pressure to create adhesion by suction to various substrates [7, 9, 14, 16, 17, 41, 42, 48, 49], which can even enable climbing vertical surfaces outside the water column [8, 11, 50–52].

Performance of the fish sucker depends on many different factors of the attachment structure itself, such as muscle contraction, kinematics, material properties, size, and shape [9, 15, 16, 22, 42, 53–58]. Additionally, the fish attachment ability is affected by the intensity of the water stream [59, 60] and the substrate curvature and its surface properties [13, 16, 17, 22, 48, 59, 61–66]. During attachment, the animals maintain their grip by friction with structures at micro- and nanoscale, i.e. papillae or microvilli [16, 19, 41, 42, 48, 67–71, 20, 21, 23]. Additionally, the mucus between and on these structures provides strong contribution to the attachment strength [72].

The whole body of the fish can be modified as ventral sucker (Balitoridae, Gastromyzontidae), or the fins have convergently evolved to attachment structures (Oxudercidae, Gobiesocidae, Cyclopteridae, Liparidae, Echeneidae, Cyprinidae) [9–19, 66]. In Gyrinocheilidae, some Gobiesocidae, and suckermouth catfishes of Africa (Mochokidae) and South America (Loricariidae, Astroblepidae), the mouth structures are transformed to an oral sucker, allowing animals to adhere to surfaces while simultaneously foraging and performing respiration [6, 7, 11, 15, 48, 61, 73–75]. This specialized suckermouth is similar to attachment structures of other fish, highly textured with papillae bearing small keratinized outgrowths of single epithelia cells, i.e., unculi [67–69, 74, 76–79].

The papillae are probably used for foraging and increasing the attachment capability by friction to reinforce the seal of the disc [48, 73, 74, 76, 80]. The unculi can be found on top of the papillae, which are highly diverse in morphology among fish [67]. In Loricariidae, they are potentially involved in feeding [73, 76], but probably increase the friction during attachment as well [6, 48, 67–69, 74, 81].

In general, attachment structures in fish have received attention in the last decades, which even enabled the development of adhesive materials or gripping and adhesive devices [42, 56, 58, 65, 72, 82–87, 20–23, 88].

However, in contrast to remoras, gobies, hillstream loaches, darters, or clingfish, which were experimentally studied with regard to attachment performance and mechanisms [9, 16, 17, 41, 42, 49, 60, 63–66, 70, 89], to date only a few experiments [48, 59] addresses the real attachment performance of the loricariid suckermouth fish.

Besides of the above-mentioned references, little is known about the structures of the suckermouth and oral papillae, even though they are highly diverse [2, 3, 7, 67, 90]. We here study the morphology of papillae and unculi of 67 species and undescribed taxa from all kinds of habitats to pave the way for more research addressing the diversity of oral discs. According to their shape, the structures were sorted to different categories. Although, comparative studies on the attachment performance of these fish taxa are lacking, we here present hypotheses on the interaction between mouth and substrate based on the diverse literature on attachment structures on different animal taxa.

## Results

### Morphology of papillae

**Fig. 1:**
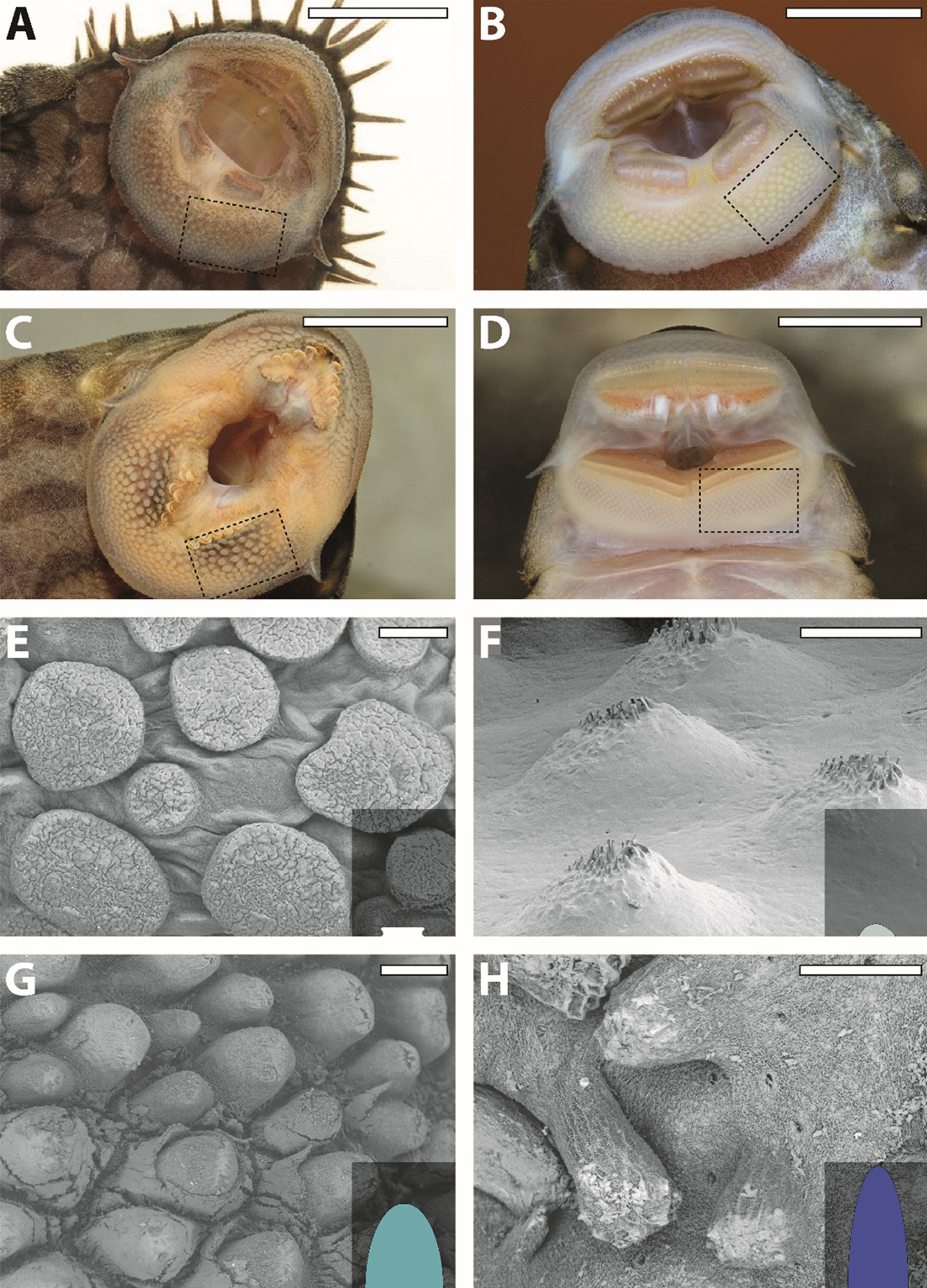
**A**–**D** Suckermouths of selected taxa. **A** *Ancistrus* sp. L464. **B** *Baryancistrus xanthellus.* **C** *Panaque nigrolineatus.* **D** *Chaetostoma formosae.* The highlighted region was documented by SEM to study the papillae and unculi. **E**–**H** Types of papillae. **E** Flat type, *Pseudohemiodon almendarizi*. **F** Short type, *Ancistrus* sp. L464. **G** Medium type, *Acanthicus adonis.* **H** Long type, *Hypancistrus* sp. L333. The sizes of the insets relate the sizes of the papillae to another. Scale bars: **A** 6 mm; **B** 6.5 mm; **C** 10.5 mm; **D** 9 mm; **E**, **F** 100 µm; **G**, **H** 250 µm

On the lower lips (Fig. 1A–D), we could differentiate four papilla types (Fig. 1E–H):

1. Flat papillae

This papilla is broad with a flattened tip (Fig. 1E). Each papilla measures between 100–200 μm in diameter and has a height of approximately 50 μm. Up to 20 papillae are found on each studied area, depending on the size of the lip. When this papilla type is manipulated, the bases seem relatively flexible and the tip rather stiff. This pattern is detected in *Ancistrus ranunculus*, *Pseudacanthicus pitanga*, *Crossoloricaria cephalaspis*, *Loricaria luciae*, *Loricaria simillima*, *Pseudohemiodon almendarizi* (Fig. 1E), *Pterosturisoma microps*, and *Spatuloricaria puganensis*.

2. Short papillae

Papillae are rather roundish in profile (Fig. 1F). They are of 100–200 μm diameter and 70–100 μm in height. When the papillae are manipulated, the tip seems rather flexible whereas the base seems rather stiff. This kind is determined for *Hypoptopoma inexspectatum*, *Ancistrus* sp. L464 (Fig. 1F), *Chaetostoma formosae*, *Scobinancistrus aureatus*, and *Rineloricaria melini*.

3. Medium papillae

These papillae are roundish and of medium height (300–400 μm) and of 100–300 μm diameter (Fig. 1G). The tip seems rather flexible, but the base rather stiff. We observed them in *Otocinclus cocama*, *Rhinotocinclus isabelae*, *Acanthicus adonis* (Fig. 1G), *Peckoltia sabaji*, *Ancistomus spilomma*, *Ancistrus cirrhosus*, *Ancistrus dolichopterus*, *Ancistrus luzia*, *Ancistrus* sp. L107, *Ancistrus* sp. L519, *Baryancistrus niveatus*, *Baryancistrus xanthellus*, *Chaetostoma dorsale*, *Chaetostoma lineopunctatum*, *Dekeyseria picta*, *Guyanancistrus brevispinis*, *Hypancistrus contradens*, *Hypancistrus inspector*, *Hypancistrus* sp. L174, *Hypancistrus zebra*, *Hypostomus cochliodon*, *Leporacanthicus joselimai*, *Leporacanthicus* cf. *galaxias*, *‘Spectracanthicus’ immaculatus*, *Panaqolus* sp. L271, *Panaqolus* sp. L351, *Panaque nigrolineatus*, *Parancistrus nudiventris*, *Pseudacanthicus* sp. L97, *Pseudacanthicus* sp. L185, *Pseudacanthicus* sp. L273, *Pseudacanthicus* sp. L65, *Pseudacanthicus spinosus*, *Pseudolithoxus dumus*, *Scobinancistrus pariolispos*, *Cteniloricaria platystoma*, *Farlowella oxyrryncha*, *Farlowella platorynchus*, *Hemiodontichthys acipenserinus*, *Lamontichthys filamentosus*, *Lamontichthys stibaros*, *Sturisomatichthys aureus*, and *Sturisomatichthys festivus*.

4. Long papillae

The papillae are roundish and long (500–550 μm) and of 80–100 μm diameter (Fig. 1H). The tip seems rather flexible, but the base rather stiff. We detect long papillae in *Ancistrus claro*, *Ancistrus megalostomus*, *Aphanotorulus horridus*, *Hypancistrus* sp. L333 (Fig. 1H), *Hypostomus bolivianus*, *Hypostomus laplatae*, *Parancistrus aurantiacus*, *Peckoltia* sp. L76, *Scobinancistrus raonii*, *Pseudorinelepis genibarbis*, and *Rhinelepis aspera*.

### Morphology of unculi

**Fig. 2:**
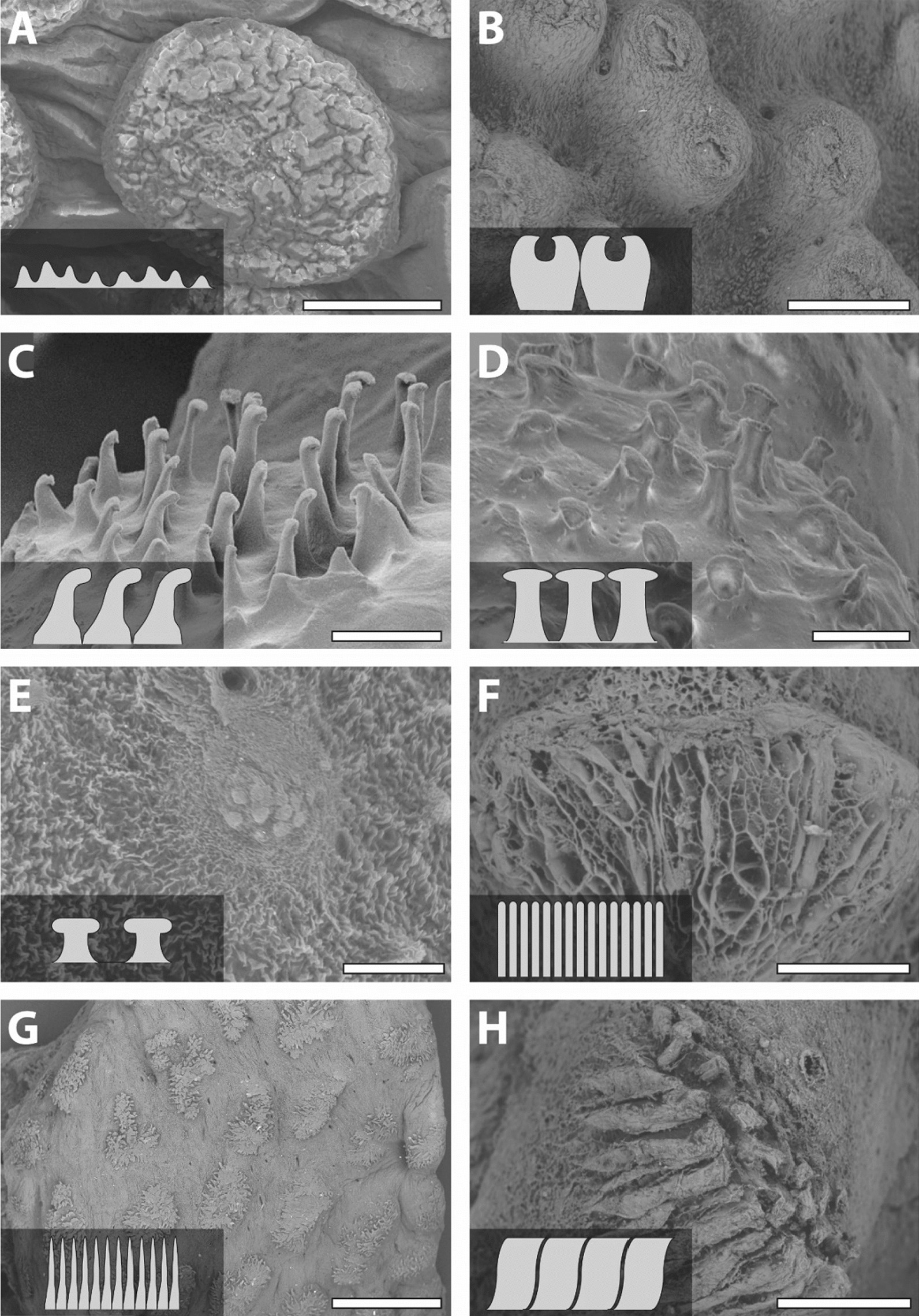
Types of unculi. **A** None is present, *Pseudohemiodon almendarizi*. **B** Suction cup, *Acanthicus adonis.* **C** Hooks, *Ancistrus* sp. L464*.* **D** Mushrooms, *Chaetostoma formosae*. **E** Small mushrooms, *Spatuloricaria puganensis*. **F** Honey-combed, *Ancistrus megalostomus*. **G** Long filaments, *Pseudacanthicus pitanga*. **H** Folds, *Hypostomus bolivianus.* Scale bars: **A**, **H**, **E** 100 µm; **B** 150 µm; **C**, **D** 15 µm; **F** 50 µm; **G** 1 mm

We could not detect a high degree of intraspecific variability in species with more than one examined specimen (Additional file 1: Figure S1). Overall, we can differentiate eight types related to the unculi on the papillae surfaces (see below) (Fig. 2).

1. No unculi

Here, no small projections but a rather bulky surface of the papilla is found (Fig. 2A). The surface seems rather stiff. We observed this in *Rhinotocinclus isabelae*, *Ancistrus ranunculus*, *Dekeyseria picta*, *Hypancistrus* sp. L174, *Hypancistrus zebra*, *Leporacanthicus joselimai*, *Leporacanthicus* cf. *galaxias*, *‘Spectracanthicus’ immaculatus*, *Parancistrus aurantiacus*, *Parancistrus nudiventris*, *Crossoloricaria cephalaspis*, *Loricaria luciae*, *Loricaria simillima*, and *Pseudohemiodon almendarizi* (Fig. 2A).

2. Suction cups (no unculi)

On the central surface of the papilla, a round to oval structure with a thick outer bulge and an inner depression is determined (Fig. 2B). The papillae themselves vary from 150 to 200 μm in diameter and the structure is of about 50 μm in diameter. The tip of each papilla seems flexible and the base rather stiff. This surface structure is detected in *Acanthicus adonis* (Fig. 2B).

3. Elongated unculi with hook-like tips

Long projections of 15–20 μm length are situated in the centre of each papilla (Fig. 2C). Each papilla hosts 20–40 single unculi, depending on the papilla size. The tips of the unculi are formed like a hook and most of them are pointed into the same direction. Tips of the hooks seem rather stiff, whereas the bases seem rather flexible. We document these unculi in *Ancistrus* sp. L464 (Fig. 2C) and *Lamontichthys stibaros*.

4. Mushroom-like unculi

Here, elongated unculi of 15–25 μm with flattened tips are found (Fig. 2D). The papillae are entirely covered with 25–40 unculi, depending on the size of the papillae. The tips seem rather stiff, whereas the bases seem rather flexible. This is observed in *Otocinclus cocama*, *Ancistrus cirrhosus*, *Ancistrus claro*, *Ancistrus luzia*, *Ancistrus* sp. L107, *Ancistrus* sp. L519, *Chaetostoma dorsale*, *Chaetostoma formosae* (Fig. 2D), *Chaetostoma lineopunctatum*, *Scobinancistrus aureatus*, *Scobinancistrus raonii*, *Cteniloricaria platystoma*, *Farlowella oxyrryncha*, *Farlowella platorynchus*, *Lamontichthys filamentosus*, *Sturisomatichthys aureus*, and *Pseudorinelepis genibarbis*.

5. Small mushroom-like unculi

In some species, unculi of 10–15 μm length with flattened tips are found (Fig. 2E). The papillae are covered with 10–15 unculi. The tips seem rather stiff, whereas the bases seem rather flexible. We document this type of unculi for *Hypancistrus contradens*, *Rineloricaria melini*, *Hemiodontichthys acipenserinus*, and *Spatuloricaria puganensis* (Fig. 2E).

6. Honey-combed pattern

Here, the whole papilla surface (150–200 μm diameter) pattern is reticulated (Fig. 2F). The parts of the structure interacting with the target surface seem rather flexible and the bases rather stiff. This type is determined for *Ancistrus megalostomus* (Fig. 2F).

7. Long filamentous unculi

Unculi are thin (~ 1 µm thick) and of 10 μm length (Fig. 2G). On each papilla, 30–40 single filaments are found. They seem very flexible. This surface pattern is detected in *Hypancistrus* sp. L333, *Pseudacanthicus pitanga* (Fig. 2G), *Pseudacanthicus* sp. L97, *Pseudacanthicus* sp. L185, *Pseudacanthicus* sp. L273, *Pseudacanthicus* sp. L65, *Pseudacanthicus spinosus*, *Sturisomatichthys festivus*, and *Rhinelepis aspera*.

8. Folds

Unculi are of 800–1000 μm length and of 90–100 μm width (Fig. 2H). On each side of the papilla, the unculi are arranged inversely. At the very tip of papilla, the unculi form a fold. The unculi seem rather flexible on their bases and stiff at their tips. This pattern is detected in *Aphanotorulus horridus*, *Baryancistrus niveatus*, *Baryancistrus xanthellus*, *Hypostomus bolivianus* (Fig. 2H), *Hypostomus laplatae*, *Panaqolus* sp. L271, *Panaqolus* sp. L351, *Panaque nigrolineatus*, *Peckoltia* sp. L76, and *Scobinancistrus pariolispos*.

### Relationship between ecology, morphology, and systematic position

Most of the studied taxa either inhabit rivers with strong or medium current (see Additional file 1: Table S1). In strong current, most species could be found on stone, followed by wood, and finally sand. Most of the species inhabiting streams with medium current inhabited wood, followed by stone, sand, and both wood and stone. Only one species was found in slow flowing water; here the studied species adhered to wood.

In strong currents, most species possessed medium papillae, followed by long, flat, and short papillae (see Additional file 1: Table S1). Most taxa bore either mushrooms or no unculi, followed by long filaments, folds, honey-combed and hooks. In medium currents, most species possessed medium-sized papillae, followed by long, short and flat papillae. Here, most species bore mushrooms or folds, followed by no unculi, small mushrooms, long filaments, hooks and suction cups. The two species inhabiting slow streams possessed long papillae with mushroom-shaped unculi; these species can be found on wood.

When the substrate is on the focus, we find that most species could be found on stone, followed by wood, sand, and finally both wood and stone (see Additional file 1: Table S1). Most species adhering to stone were found in rivers with strong current, followed by medium current. Wood as preferred substrate seemed to be mostly inhabited in medium currents, followed by strong and slow current. Most species inhabiting sand were found in streams with medium, followed by strong current. Both wood and stone were inhabited in medium currents.

Most stone-inhabitants possessed medium papillae, followed by long, short and flat papillae (see Additional file 1: Table S1). Here, most taxa bore no unculi, followed by long filaments and mushrooms, folds, and finally honey-combed papillae and small mushrooms. When wood was preferred, most taxa possessed medium papillae, followed by long, short, and finally flat ones. Most species bore mushrooms, followed by folds, hooks, and finally long filaments, no unculi or suction cups. Sand-inhabitants mostly possessed flat papillae, followed by medium and short ones. Here, taxa bore mostly no unculi or small mushrooms. The two species adhering to both wood and stones possessed medium papillae with either long filaments or mushrooms.

**Fig. 3:**
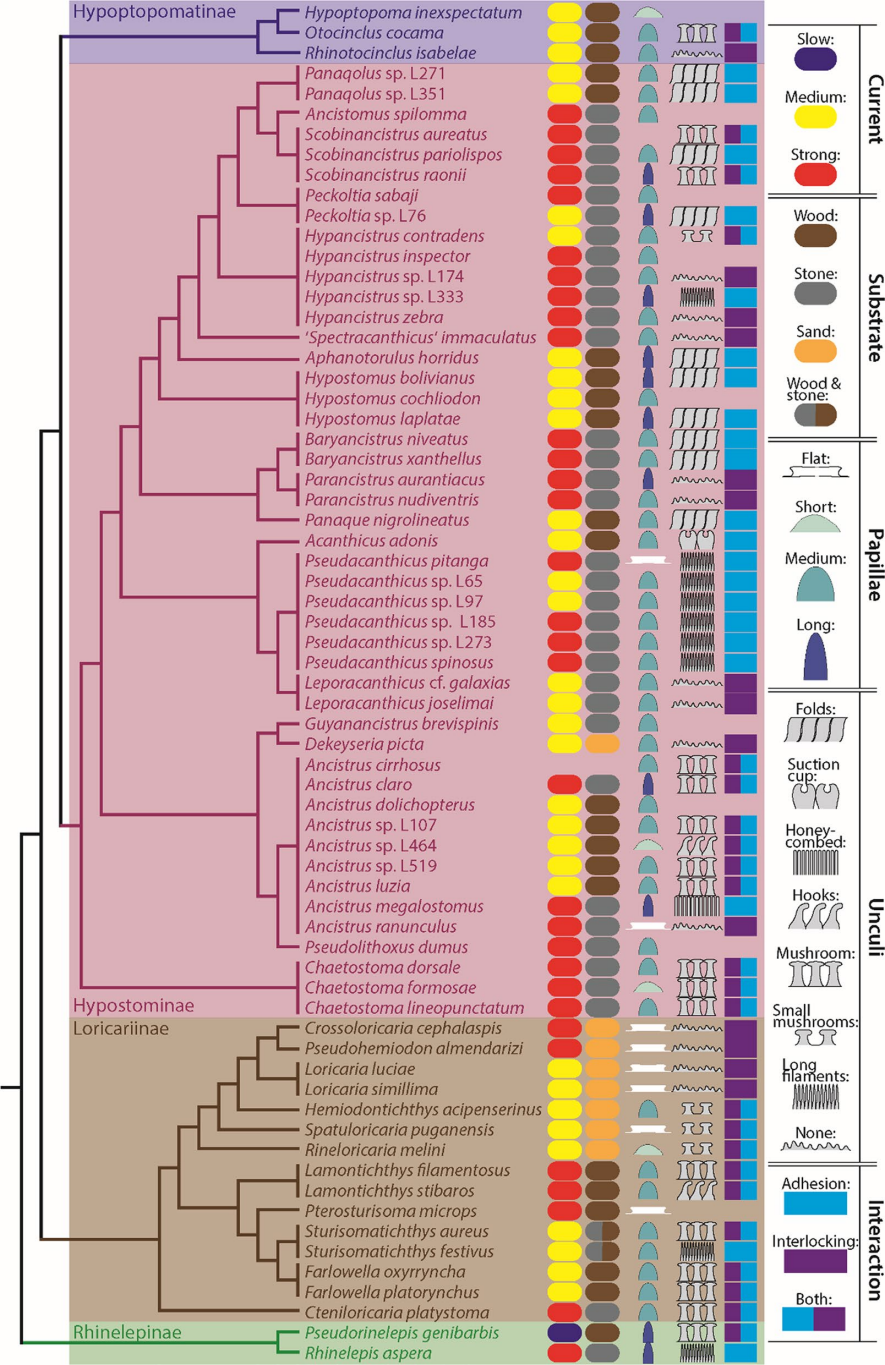
Summary of ecological data and results from morphological analyses, visualized on a cladogram. From left to right: current type, preferred substrate, papilla type, unculi type, proposed type of interaction between substrate and organism – for each species studied. When the field is empty, no data was available

With regard to the phylogenetic position (Fig. 3), we found that species inhabiting streams with strong current belonged to Hypostominae, Loricariinae, and Rhinelepinae. Rivers with medium current were colonized by Hypoptopomatinae, Hypostominae, and Loricariinae. The one species living in slow flowing waters belonged to the Rhinelepinae. In most cases, when more than one species of a genus was studied (*Leporacanthicus*, *Panaqolus*, *Scobinancistrus*, *Parancistrus*, *Baryancistrus*, *Chaetostoma*, *Hypostomus*, *Loricaria*, *Lamontichthys*, *Sturisomatichthys*, *Farlowella*), we found that the taxa of the same genus preferred the same current type. In some cases (*Pseudacanthicus*, *Hypancistrus*, *Ancistrus*, *Peckoltia*), species of the same genus inhabited different current types (Fig. 3).

Preference of stone could be determined in some taxa of Hypostominae, Loricariinae, and Rhinelepinae (Fig. 3). Wood-preference was found in Hypoptopomatinae, Hypostominae, Loricariinae, and Rhinelepinae. Sand-dwelling was observed in *Dekeyseria* (Hypostominae) and some Loricariinae taxa. Both wood and stones were only inhabited by *Sturisomatichthys*, belonging to Loricariinae. In most cases, when more than one species of a genus was studied (*Pseudacanthicus*, *Leporacanthicus*, *Panaqolus*, *Scobinancistrus*, *Parancistrus*, *Hypancistrus*, *Peckoltia*, *Baryancistrus*, *Chaetostoma*, *Hypostomus*, *Loricaria*, *Lamontichthys*, *Sturisomatichthys*, *Farlowella*), we found that the taxa of the same genus preferred the same substrate. Only in *Ancistrus*, species adhere to different substrates (Fig. 3).

Flat papillae were detected in Hypostominae and Loricariinae (Fig. 3). The short type was determined for Hypoptopomatinae, Hypostominae, and Loricariinae. Medium-sized ones were identified for Hypoptopomatinae, Hypostominae, and Loricariinae. Large ones were found in Hypostominae and Rhinelepinae. In some cases, when more than one species of a genus was studied (*Leporacanthicus*, *Panaqolus*, *Baryancistrus*, *Loricaria*, *Lamontichthys*, *Sturisomatichthys*, *Farlowella*), we found that the taxa of the same genus possessed the same papilla type. In most cases (*Pseudacanthicus*, *Scobinancistrus*, *Hypancistrus*, *Ancistrus, Chaetostoma, Hypostomus*, *Parancistrus*, *Peckoltia*), species of the same genus showed different types (Fig. 3).

Unculi of the fold type were determined in Hypostominae (Fig. 3). The suction cups were only found in *Acanthicus adonis* (Hypostominae) and the honey-combed type in *Ancistrus megalostomus* (Hypostominae). Hooks were detected in *Ancistrus* sp. L464 (Hypostominae) and *Lamontichthys stibaros* (Loricariinae). Mushrooms were determined in Hypoptopomatinae, Hypostominae, Loricariinae, and Rhinelepinae. Small mushrooms were found in *Hypancistrus contradens* (Hypostominae) and some Loricariinae. Long filaments were detected in taxa of Hypostominae, *Sturisomatichthys festivus* (Loricariinae), and *Rhinelepis aspera* (Rhinelepinae). No unculi were found in some species of Hypoptopomatinae, Hypostominae, and Loricariinae. In most cases, when more than one species of a genus was studied (*Pseudacanthicus*, *Leporacanthicus*, *Panaqolus*, *Parancistrus*, *Baryancistrus*, *Chaetostoma, Hypostomus, Loricaria*, *Farlowella*), we found that the taxa of the same genus possessed the same unculi type. In some cases (*Scobinancistrus*, *Hypancistrus*, *Ancistrus, Lamontichthys*, *Sturisomatichthys*), species of the same genus showed different types (Fig. 3).

### Proposed interaction between organisms and substrate

**Fig. 4:**
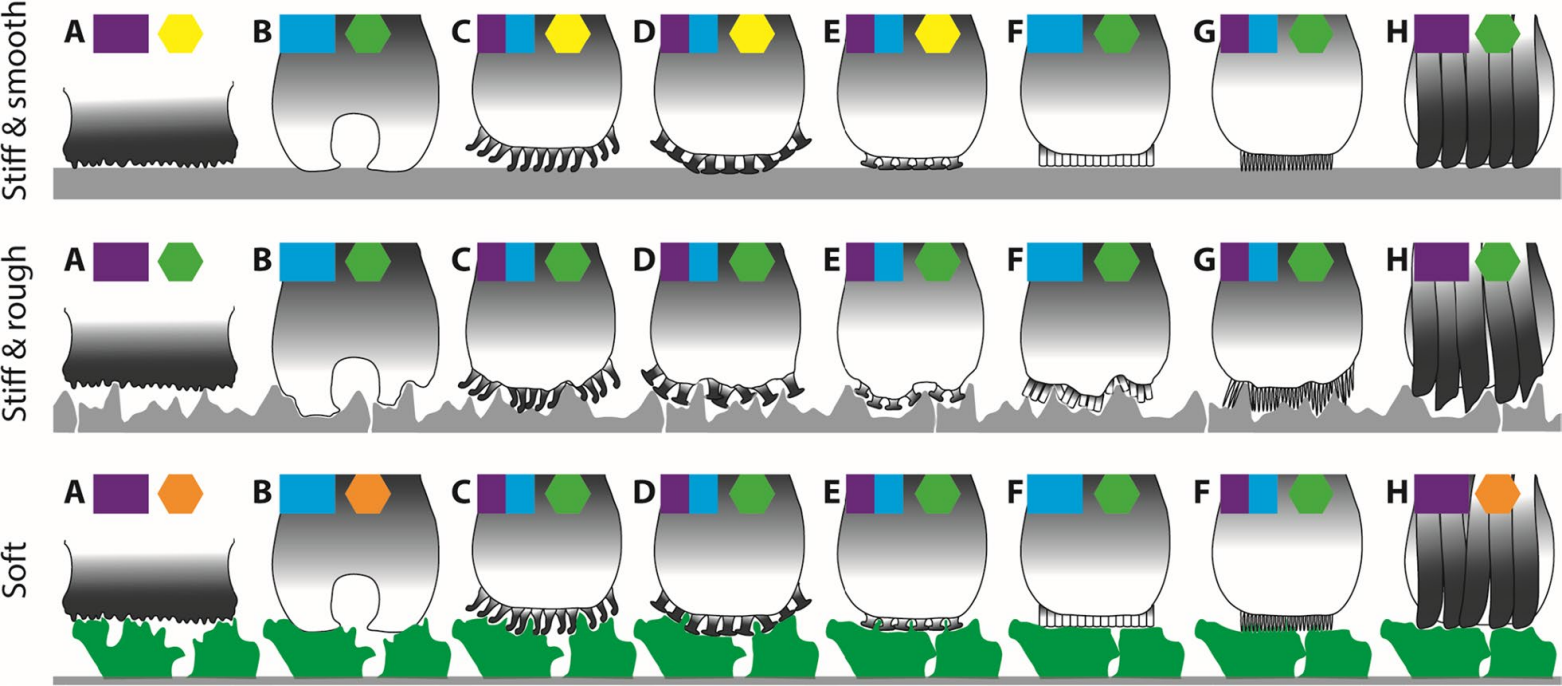
Proposed interaction between some papillae and unculi types and some substrate occurring naturally in the habitat (stiff and smooth as e.g. rounded stone or wood; stiff and rough as e.g. wood or stone; soft substrate as e.g. plant covers on stone or wood). The grey color gradients in the structures relate to the mechanical properties (black = stiff; white = flexible), which are inferred from manipulation of structures and by documenting the artefacts caused by drying and shrinking. Purple boxes identify the structures, which probably facilitate interlocking (interlocking type) and the blue boxes identify the structures, which probably support adhesion to the target surface (adhesion type). Hexagons show, how presumably well the specific structures enable attachment with the target surface (orange = not well, yellow = medium, green = well). **A** Flat papillae with no unculi but a bulky surface. **B** Medium papillae with suction cups. **C** Medium papillae with hooks. **D** Medium papillae with mushrooms. **E** Medium papillae with small mushrooms. **F** Medium papillae with honey-combed unculi. **G** Medium papillae with long filaments. **H** Medium papillae with folds

As comparative experimental data on species with different mouthpart morphologies is lacking, we can only hypothesize about their attachment capabilities. However, attachment structures were well investigated in various animal phyla and basic principles of adhesion and interlocking are known, we can infer some functionality based on the morphology analysis together with material property estimations and propose hypotheses about the interaction of papillae and unculi with some different kinds of substrate that can be found in nature (Fig. 4). These substrate types include stiff and smooth (stones or wood that were smoothened by the stream), stiff and rough (stones or wood), or soft substrate (plant covers on stone or wood).

The flat papillae (detected in *Pseudacanthicus pitanga*, *Ancistrus ranunculus*, *Crossoloricaria cephalaspis*, *Pseudohemiodon almendarizi*, *Loricaria luciae*, *Loricaria simillima*, *Spatuloricaria puganensis*, and *Pterosturisoma microps*) seem to be composed of rather rigid material, which is embedded in the softer and more flexible lip (Fig. 4A). In some species (*Ancistrus ranunculus*, *Crossoloricaria cephalaspis*, *Pseudohemiodon almendarizi*, *Loricaria luciae*, and *Loricaria simillima*), no unculi were detected and the papilla surface seems to be rather bulky. During attachment, the papillae are probably capable of interlocking with rather stiff and rough substrate (*Ancistrus ranunculus*), which is facilitated by the soft embedment. We, however, expect these species to underperform on stiff and smooth substrates and to hardly attach to soft substrates (plant covers), since contact areas are reduced (Fig. 4A). This could potentially explain, why this pattern is mostly found in species living on sand or mud (*Crossoloricaria cephalaspis*, *Pseudohemiodon almendarizi*, *Loricaria luciae*, and *Loricaria simillima*), where attachment will probably not play such an important role. Here, the species probably only temporarily attach to substrate (e.g., to rough substrate or wood) and might not need a tight and more continuous attachment. However, thick mucus, covering the bulky surface, might compensate these shortcomings, which should be investigated in the future. However, in some species (*Pseudacanthicus pitanga*, and *Spatuloricaria puganensis*), these papillae are covered by flexible long and thin filaments or by small mushrooms. Here, we expect the unculi to adapt to the substrate increasing either adhesion (by filaments) or interlocking (by mushrooms) (Fig. 4E,G).

The short papillae were detected in *Hypoptopoma inexspectatum*, *Scobinancistrus aureatus*, *Ancistrus* sp. L464, *Chaetostoma formosae,* and *Rineloricaria melini*. These papillae seem to have limited range of motion as well, but could potentially function as bolsters, when the unculi interact with the substrate, or support rearrangement during attachment, because the papillae tips seem to be flexible. They were either covered by unculi of the mushroom type (*Scobinancistrus aureatus*, *Chaetostoma formosae*), hooks (*Ancistrus* sp. L464), or short mushrooms (*Rineloricaria melini*). Here, unculi together with the flexible papillae could enable a tight interaction with the stiff and rough or with the soft substrate by interlocking and with the stiff and smooth one by adhesion (Fig. 4C–E). However, mucus could also be potentially distributed between the unculi and additionally support adhesion under water.

The medium-sized papillae were detected in most studied species. Due to the length of the papillae we expect this type to have a higher range of motion, which presumably enables them to adapt to rather challenging surfaces. The bases of the papillae seem to be stiffer and the tips more flexible, and therefore we expect high attachment forces, as the flexible papilla tips (which make unculi bases flexible) can easily adapt to corrugated substrates and to interact with them. They were usually covered with unculi, either with mushrooms (*Otocinclus cocama*, *Ancistrus* sp. L519, *Ancistrus* sp. L107, *Ancistrus cirrhosus*, *Ancistrus luzia*, *Chaetostoma dorsale*, *Chaetostoma lineopunctatum*, *Lamontichthys filamentosus*, *Sturisomatichthys aureus*, *Farlowella platorynchus*, *Farlowella oxyrryncha*, and *Cteniloricaria platystoma*), hooks (*Lamontichthys stibaros*), folds (*Panaqolus* sp. L271, *Panaqolus* sp. L351, *Panaque nigrolineatus*, *Scobinancistrus pariolispos*, *Baryancistrus xanthellus*, and *Baryancistrus niveatus*), long filaments (*Pseudacanthicus spinosus*, *Pseudacanthicus* sp. L97, *Pseudacanthicus* sp. L65, *Pseudacanthicus* sp. L185, *Pseudacanthicus* sp. L273, and *Sturisomatichthys festivus*) or short mushrooms (*Hypancistrus contradens* and *Hemiodontichthys acipenserinus*). The species bearing mushrooms, hooks, or short mushrooms can probably interact with the stiff and rough substrates and the soft substrate (plants, biofilm, etc.) by interlocking and with the stiff and smooth substrate by adhesion (Fig. 4C–E). However, on stiff and smooth substrates, contact points might be reduced, since less unculi are in contact. For the species bearing long filaments, we expect a high degree of interlocking on stiff and rough or soft substrates and of adhesion on stiff and smooth substrate, as these soft structures seem flexible enough to establish contact on most surfaces (Fig. 4G). The unculi of the fold type are potentially rather used for establishing contact by interlocking, since their tips seem to be rather stiff (Fig. 4H). We expect this type to underperform on soft substrates (i.e., plant covers); however, mucus between these structures might increase their attachment ability. In one species (*Acanthicus adonis*), we determined medium papillae with a suction cups surface pattern. Due to morphology and material property estimation, we expect this type to adhere tightly with stiffer surfaces (both smooth and rough), but underperform on soft substrates (Fig. 4B). Only a few species (*Rhinotocinclus isabelae*, *Leporacanthicus joselimai*, *Leporacanthicus* cf. *galaxias*, *‘Spectracanthicus’ immaculatus*, *Parancistrus nudiventris*, *Hypancistrus* sp. L174, *Hypancistrus zebra*, and *Dekeyseria picta*) did not have unculi but rather a bulky surface (Fig. 4A). For these species, we expect an interlocking mechanism to be present. In this case, the relatively flexible papilla bases probably adapt to the target surface and the stiffer bulky surface enables interlocking with the rough and stiff substrate.

Long papillae were detected in some species (*Scobinancistrus raonii*, *Peckoltia* sp. L76, *Parancistrus aurantiacus*, *Hypancistrus* sp. L333, *Ancistrus claro*, *Ancistrus megalostomus*, *Aphanotorulus horridus*, *Hypostomus bolivianus*, *Hypostomus laplatae*, *Pseudorinelepis genibarbis*, and *Rhinelepis aspera*). Because of their length, we expect these papillae to adapt best to very rough surfaces, due to an increased range of motion. On top of these papillae, we detected either mushrooms (*Scobinancistrus raonii*, *Ancistrus claro*, and *Pseudorinelepis genibarbis*), folds (*Peckoltia* sp. L76, *Aphanotorulus horridus*, *Hypostomus bolivianus*, and *Hypostomus laplatae*), bulky surface without unculi (*Parancistrus aurantiacus*), long filaments (*Hypancistrus* sp. L333 and *Rhinelepis aspera*) or honey-combs (*Ancistrus megalostomus*). Since the mushrooms, long filaments and honey-combs seem to be flexible, we expect these structures to adhere to any surface either by interlocking or by adhesion (Fig. 4D,F,G). For the folds, which seem to be stiffer at their tips, we expect an underperformance on soft plant surfaces (Fig. 4H) due to their limited ability to adapt their shape to the target substrate, which would hinder the oral disc to form an effective seal. The bulky surfaces (Fig. 4A) potentially also underperform on soft plant surfaces, due to the limited flexibility, and on stiff and smooth surfaces, because interlocking is here not facilitated.

### Ancestral state reconstruction

Ancestral state reconstruction (Additional file 1: Figure S3) suggested that Loricariidae lived initially lived in medium or strong currents. Only *Pseudorinelepis genibarbis* (Rhinelepinae) colonized slow-flowing rivers. Based on the tree, 19 shifts between streams with different velocities were reconstructed.

With regard to the substrate, stone seemed to be ancestral (Additional file 1: Figure S4). There were at least 11 shifts between substrate types, mostly from stony substrates to wood. Only few species live in streams with sandy substrates, but even this habitat type has been colonized twice, in a larger clade of the Loricariinae and in *Dekeyseria picta* (Hypostominae).

With regard to the papillae (see Additional file 1: Figure S5), either the medium or long papilla was ancestral. In the group containing Loricariinae, Hypostominae, and Hypoptopomatinae the medium-sized papillae evolved and were kept in most lineages. Long papillae are found in all examined Rhinelepinae, but convergently evolved from medium papillae at least seven times convergently within Hypostominae. The flat papilla evolved at least four times convergently from the medium-sized type (within Loricariinae: in *Pterosturisoma microps*, *Spatuloricaria puganensis*, and the group containing *Crossoloricaria* and *Loricaria* highlighted in Additional file 1: Figure S5 by a blue box; within Hypostominae: in *Ancistrus ranunculus* and *Pseudacanthicus pitanga*). Medium papilla evolved at least five times into short papillae.

With regard to the unculi, the ancestral state reconstruction suggested that mushrooms were ancestral (Additional file 1: Figure S6). Mushrooms were replaced by long filaments at least two times convergently (in Rhinelepinae: in *Rhinelepis aspera*; in Loricariinae: in S*turisomatichthys festivus*). Evolution from mushrooms to hooks convergently happened in *Lamontichthys stibaros* and in *Ancistrus* sp. L464. Mushrooms convergently evolved into small mushrooms in *Hypancistrus contradens* and ‘*Spectracanthicus*’ *immaculatus* in Hypostominae and in the group of Loricariinae highlighted in Additional file 1: Figure S6 by a blue box. In the latter, small mushrooms were lost in the clade containing *Crossoloricaria*, *Pseudohemiodon*, and *Loricaria*. In Hypostominae, mushrooms were lost several times, but apparently were regained in *Scobinancistrus aureatus* and *Scobinancistrus raonii*. Mushrooms evolved into honey-combs in *Ancistrus megalostomus*. In the group highlighted in Additional file 1: Figure S6 by a red box, folds evolved, which were lost convergently again. Long filaments convergently evolved at least four times, whereas suction cusps evolved only in *Acanthicus adonis*. In Hypoptopomatinae, the mushrooms were lost in *Rhinotocinclus isabelae*.

With regard to the interaction type, ancestral state reconstruction suggested that both adhesion and interlocking occurred in the ancestral state (Additional file 1: Figure S7). Within Rhinelepinae, Loricariinae, and Hypostominae, adhesion-dominated interaction convergently evolved at least four times (in *Rhinelepis aspersa*, *Ancistrus megalostomus*, *Sturisomatichthys festivus*, the group containing *Acanthicus*, *Leporacanthicus*, and *Pseudacanthicus*). Within the latter group, this shifted to interlocking in *Leporacanthicus*. Using both adhesion and interlocking was convergently replaced by interlocking-dominated attachment at least five times (in Loricariinae: in the group containing *Crossoloricaria*, *Pseudohemiodon*, and *Loricaria*; in Hypoptopomatinae: in *Rhinotocinclus isabelae*; in Hypostominae: in *Dekeyseria*, in *Ancistrus ranunculus*, in the group containing *Panaque* and *Ancistomus* highlighted in Additional file 1: Figure S7 by a red box). In the latter group, interlocking changed to adhesion-dominated attachment in *Hypancistrus* sp. L333 and to both types of interaction convergently in *Hypancistrus contradens*, *Scobinancistrus aureatus*, and *Scobinancistrus raonii*.

## Discussion

We here aim at presenting the structural diversity of papillae and unculi types in Loricariidae. As here only 67 species were studied, we expect that more diversity can be potentially discovered, when more species are included in such a study.

The loricariid oral disc is composed of upper and lower jaw and is surrounded by a softer outer rim, which was found to make tight contact with the substrates during attachment [6]. This seems similar to the soft tissues surrounding the suckers of remoras, which conform to the local roughness and curvature of the substrate [91], or to the outer papillae, setae or microvilli (which are of smaller diameter and densely packed) of the clingfish and loaches [16, 17, 41, 42, 70, 71].

The fleshy lips of Loricariidae are highly variable with regard to morphology, size and the content of collagen, which was previously found to relate to the substrate and the flow [92]. The collagen probably reinforces the oral suction cups and reduces slipping, failure or buckling in streams with high flow velocities [92], while being manipulated and bolstered by the jaws and maxillary barbels [73]. The lips are covered ventrally by uniculiferous papillae [73, 74, 76, 80], which probably increase wet friction and hydrodynamic adhesion to reinforce the seal of the oral disc [48, 80]. The geometry and arrangement of papillae in other fish taxa were previously found to support the resistance to shear forces and to arrests cracks at the interface between suction cups and substrate, which would compromise the subambient pressure in the mouth chamber [16, 17, 41, 70, 71]. This mechanism is partially similar to segmented adhesive pads of insects [27, 32, 93].

The unculi, which can be found on top of the papillae, are potentially involved in feeding [73, 76, 81]. But additionally, they probably increase the friction/interlocking during attachment on rough substrates [6, 48, 67, 74, 81]. This, together with the mucus, increases attachment strength as in other fish taxa [9, 72, 94].

With regard to interspecific variation of papilla size and morphology in Loricariidae, there is a huge lack of knowledge. In the here examined species, we were able to recognize four papilla types (Fig. 1), which differ in their height and range of motion, and eight unculi types (Fig. 2).

The reconstructions of ancestral habitats and character states (Additional file 1: Figures S3-S7) suggested that the different habitat types were colonized repeatedly and the states of all studied traits convergently evolved multiple times in Loricariidae. High levels of convergent evolution were previously also proposed for mandibles and body shapes in suckermouth armoured catfishes [2, 3] and for foot adhesive pads in animals [32].

With regard to the ecological data collected in the field, the current (slow, medium, strong) and the preferred substrate (stone, wood, sand) type, we could detect that medium and long papillae were often found in species inhabiting strong and medium currents. The length of the structure probably relates positively to the attachment ability on most types of substrate, except surely for sand. This is likely important in strong currents, since with higher current velocities higher forces act on the fish and thus a higher attachment is needed. With regard to the unculi we found that folds, mushrooms, as well as no unculi related to medium and strong currents. Here, we propose that the presence of unculi and the surface pattern of the papillae increase the attachment performance by interlocking, which is especially necessary in strong currents. This however awaits further investigation in e.g. the course of controlled experiments on model species with different unculi and papilla types. The comparison of the ancestral state reconstructions (Additional file 1: Figures S3-S7) revealed no obvious correlations between habitat shifts and the evolution of specific character states.

Since comparative experiments on species with different mouthpart morphologies have not been performed, we could only hypothesize about their attachment capabilities to some natural surfaces that are omnipresent in the fish habitat (stiff and smooth as e.g. rounded stones and wood; stiff and rough as e.g. rough stones or wood; soft as e.g. plants covering stones or wood) in this manuscript. However, our hypotheses were based on the knowledge about attachment structures in other animal phyla and the basic principles of adhesion and interlocking. With this we could infer some functionality based on the morphology analysis together with material property estimations and propose hypotheses about the interaction of papillae and unculi with different kinds of substrate (Fig. 4). Since attachment in gobies was previously found to rely on the ability to form an effective seal and to underperform on rougher surfaces [13, 49], we expect therefore that the suction cusp unculi type underperforms on stiff and rough surfaces as well.

We, however, do not know the position of the unculi and the papillae during attachment, which hopefully can be tested experimentally in the future. In addition to the micro- and nanostructures, the mucus covering the mouth apparatus is on expect to contribute to the contact formation and adhesion as well and therefore should be investigated deeply in the future.

## Conclusion

The oral discs of suckermouth armoured catfish (Loricariidae), which enable attachment and interlocking to various natural surfaces, are highly diverse with regard to the morphology of the papillae and of the unculi, small horny projections. Here, we studied 67 taxa and determined four papilla types and eight unculi types. From handling the structures and from drying artefacts we could infer some information about their material properties. This, together with their shape, enabled us to carefully propose hypotheses about mechanisms of interactions of oral disc structures with natural substrates typical for respective fish species. Reconstructions of ancestral habitats and states indicated frequent habitat shifts and a highly convergent evolution of most character states in Loricariidae. There is no obvious correlation between habitat shifts and the evolution of specific character states.

## Methods

### Specimens and preparation

In this project, 67 species and undescribed taxa (here named according to their L numbers) were studied (see Additional file 1: Table S1). For each species, between one and five specimens were examined, depending on the availability of material: some species are quite rare and only one specimen could be gathered, whereas for other taxa more individuals could be used (see Additional file 1: Table S1). From the species with a higher quantity of individuals, we were able to study the intraspecific variability with regard to the morphology of the adhesive suckermouth papilla structures. Since this was not high, we decided to include the species with only one specimen in this study as well. As papillae and unculi are regularly shed, we investigated every papilla on the studied lip part to obtain information about unculi types.

We did not experiment with living fish or kill fish for this study; instead we used the network of German suckermouth catfish owners (they were kept either directly by DKV or other hobbyists) which provided us with specimens that died naturally in the aquariums (fish died between 1995 and 2022). All animals were wild caught, imported to Germany by the ornamental fish trade, and then kept by hobbyists. Fish were, directly after death, fixed and stored in 70% EtOH.

In general, suckermouths are very variable between species. To illustrate this diversity, we have included some images of alive fish in the Additional file 1: Figures S8-S23. For this study, we studied the lower left lip of the specimens, which were carefully dissected using scalpel and forceps. Samples were stored in 70% EtOH and cleaned from mucus by a short ultrasonic bath for 2 min. The samples were dried in a critical point dryer (Betta-Tech-Controls, Blakelands, UK). For this method, samples were transferred to 100% EtOH first, then to the liquid CO_2_ and then slowly critical point dried from the CO_2_.

### Scanning electron microscopy

Lips were attached to scanning-electron-microscopy (SEM) sample holders by double-sided adhesive carbon tapes. Samples were then sputter-coated with gold–palladium (layer of 10 nm) employing a Leica EM SCD400 (Leica Microsystems, Wetzlar, DE). All samples were documented with a Scanning Electron TM3000 Tabletop Microscope (Hitachi, Tokyo, JP). All images were taken with different magnifications (30x – 2500x) at 5 kV.

Some lips from species with multiple animals at hand were investigated more detailed employing a cryo-SEM S4800 (Hitachi, Tokyo, JP) equipped with the Gatan ALTO-2500 cryo-preparation system (Gatan, Abingdon, UK). For this purpose, the lips were carefully attached to SEM sample holders and then frozen in liquid nitrogen at – 210 °C, to avoid the formation of ice crystals, in the cryo-preparation chamber. Then, the temperature was raised to − 95 °C, initiating the process of sublimation (freezing-drying). After sublimation for 5–7 min., the temperature was reduced to − 140 °C and the sample was sputter-coated by gold–palladium (layer of 3–4 nm) directly in the cryo-preparation chamber. Afterwards the samples were transferred into the SEM and observed at – 120 °C at 3 kV accelerating voltage.

We concentrated on two different hierarchical levels of the oral disc morphology: the labial papillae and the unculi on the papillae. In few species, the unculi type could not be determined due to high content of mucus. In these cases, we were only able to collect data on papilla morphology.

### Material property estimation

We did not perform any material property tests for this manuscript. However, from the manipulation of papillae before critical point drying by forceps and from the observation of artefacts resulting from drying and shrinking of the unculi and the papillae, we can propose some hypotheses about the relative stiffness and flexibility of different sites of samples. We hope that the characterization of material properties of different parts of the sucker can be addressed by using micro- and nanoindentation in the future.

### Data on ecology

Since data on the precise microhabitat of each species are lacking and await future investigations, we here rather chose rather broad categories (see Additional file 1: Table S1). The current or stream (macro habitat) was categorized in slow, medium and strong. We use this term according to its meaning as a flowing body of water or a ‘continuous flow of a liquid’. It can either be a small creek with slow current, a broad river with fast current, or a stream in between. The preferred substrate (micro habitat) was wood, stone, wood and stone, or sand. This data was obtained from the personal observation of the collectors, mainly DKV, in the field (see Additional file 1: Figure S2 and Table S1).

### Systematization

We studied 67 species belonging to four subfamilies of Loricariidae: Hypoptopomatinae, Loricariinae, Rhinelepinae and Hypostominae (see Additional file 1: Table S1). To gain insight, whether the specific morphology of the suckermouth structures relates to phylogeny, we plotted the data obtained on a cladogram based on recent phylogenies [95–98]; personal comment from Jon Armbruster, Auburn University]. Within each genus, we sorted the species in alphabetical order, since there is, to the best of our knowledge, no phylogeny which includes all of the species studied.

### Character state evolution

Ancestral states of habitats and characters at internal nodes were reconstructed under maximum parsimony assumptions using the Trace Character History command in Mesquite [99]. Current velocity (slow, medium, strong) and interaction states (adhesion, both, interlocking) were treated as ordered; substrate states (wood, stone, sand) were treated as unordered. Transitions between flat, short and medium papillae as well as between medium and long papillae were counted as one step; transitions between flat or short and long papillae were counted as two steps. Unculi were classified into two categories, one including folds, suction cups and honey combed unculi, the other including hooks, mushrooms, small mushrooms and long filaments. Transitions within a category were counted as one step; transitions between the two categories were counted as two steps. Transitions between any state of unculi and no unculi were counted as one step.

### Supplementary Information

**Additional file 1.** Supplementary material including list of species with localities, images of the localities, and the intraspecific variability of unculi.

## Data Availability

The datasets are included in the supplementary.
