# Supplementary material for: Holding in the stream: convergent evolution of suckermouth structures in Loricariidae (Siluriformes)
Source: Front Zool. 2023 Dec 1;20:37. doi: 10.1186/s12983-023-00516-w (PMC10691160; doi:10.1186/s12983-023-00516-w)
Supplement: Supplementary file 1 — Additional file 1. Supplementary material including list of species with localities, images of the localities, and the intraspecific variability of unculi. [file 12983_2023_516_MOESM1_ESM.docx]

Wencke Krings^1,2,3,4*^, Daniel Konn-Vetterlein^1^, Bernhard Hausdorf^5^, Stanislav N. Gorb^1^

^1^ Department of Functional Morphology and Biomechanics, Zoological Institute, Christian-Albrechts-Universität zu Kiel, Am Botanischen Garten 1–9, 24118 Kiel, Germany

^2^ Department of Cariology, Endodontology and Periodontology, Universität Leipzig, Liebigstraße 12, 04103 Leipzig, Germany

^3^ Department of Mammalogy and Palaeoanthropology, Leibniz Institute for the Analysis of Biodiversity Change, Martin-Luther-King-Platz 3, 20146 Hamburg, Germany

^4^ Department of Electron Microscopy, Institute of Cell and Systems Biology of Animals, Universität Hamburg, Martin-Luther-King-Platz 3, 20146 Hamburg, Germany

^5^ Department of Malacology, Leibniz Institute for the Analysis of Biodiversity Change, Martin-Luther-King-Platz 3, 20146 Hamburg, Germany

*corresponding author: wencke.krings@uni-hamburg.de

**Supplementary Material**

**
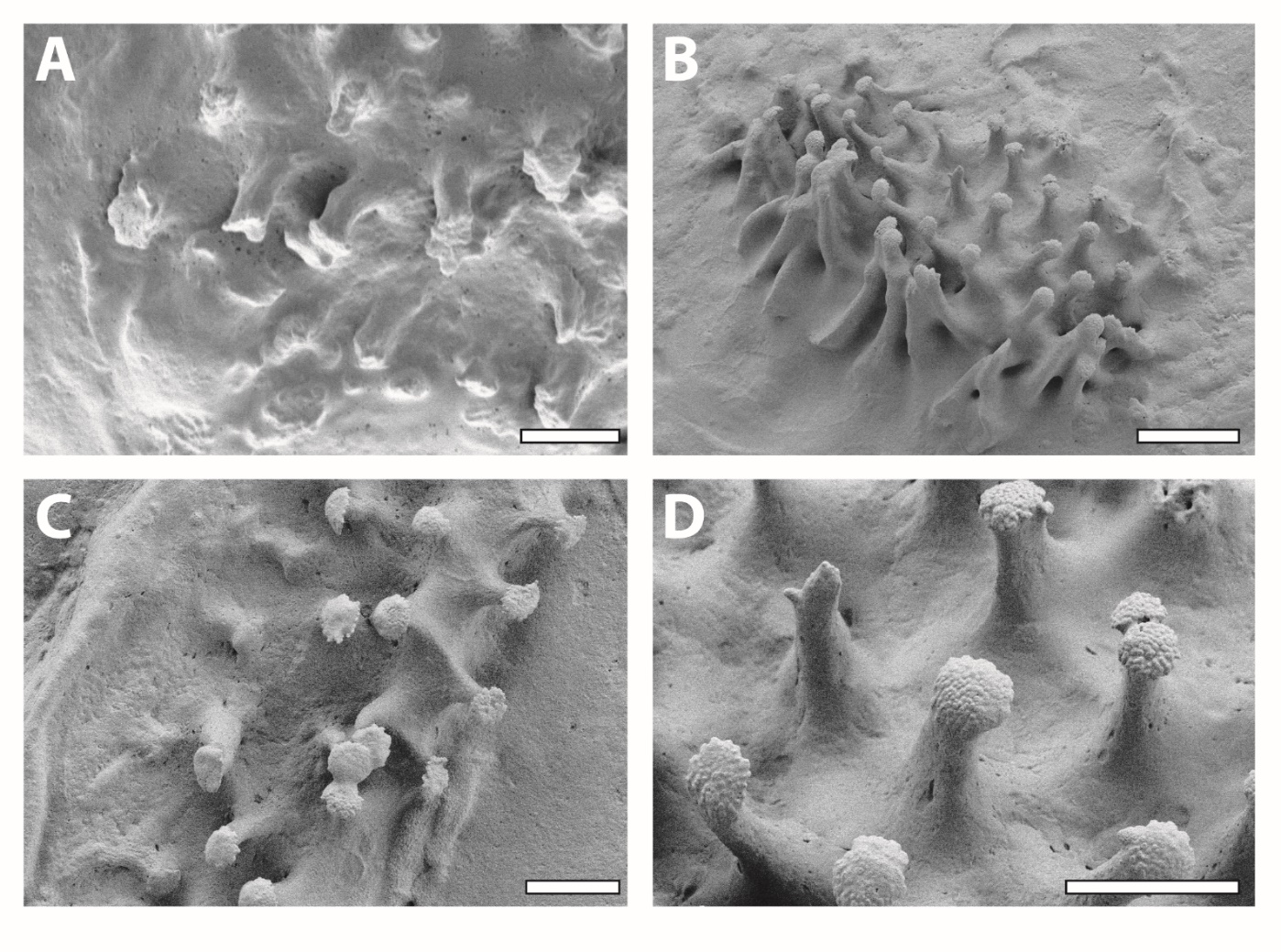
Supplementary Figure 1.** Unculi (mushrooms) of the four documented specimens of *Ancistrus* sp. L519. Each image comes from one specimen. Scale bars: A, C, D, 10 µm; 20 µm.


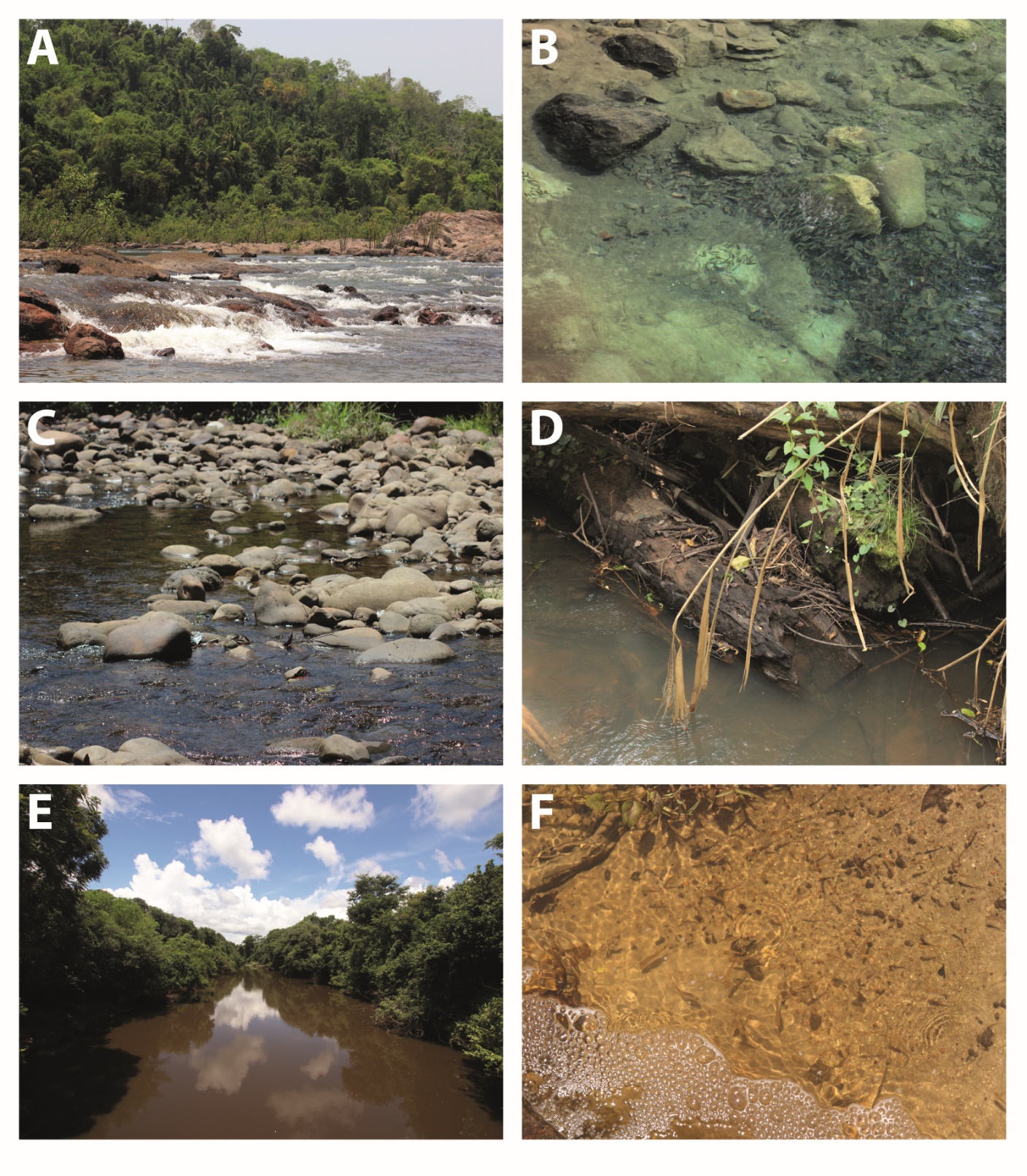


**Supplementary Figure 2. Different habitats and preferred substrate.** A. Strong current (Rio Xingu, Brazil). B. Stone substrate (Río Carrasco, Bolivia). C. Medium current (Río El Fuerte, Bolivia). D. Wood substrate (Río Tucavaca, Bolivia). E. Slow current (Río Tucavaca, Bolivia). F. Sandy substrate (Río Curicha, Bolivia).

**
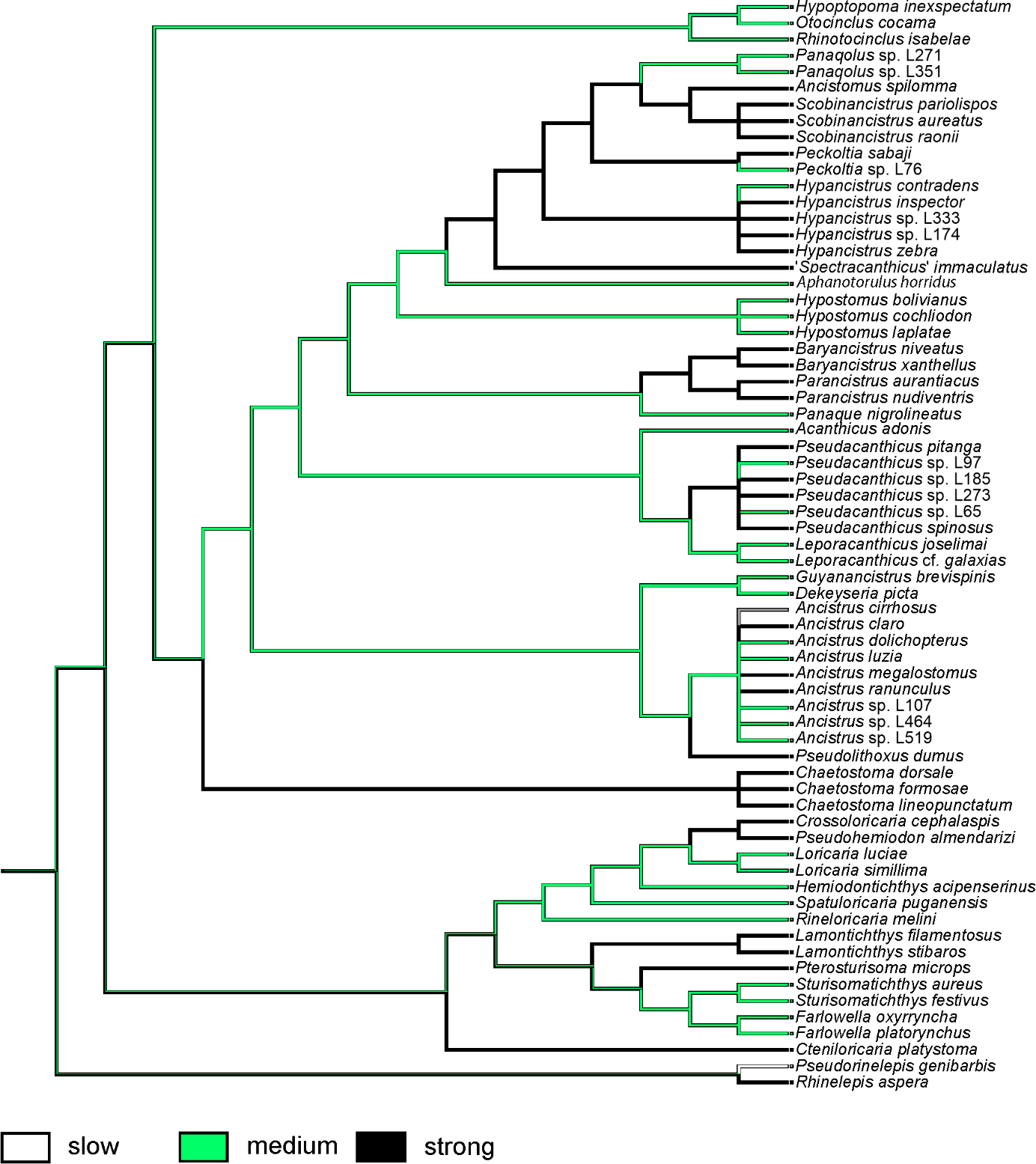
**

**Supplementary Figure 3. Reconstruction of ancestral current types (grey: habitat unknown).**

**
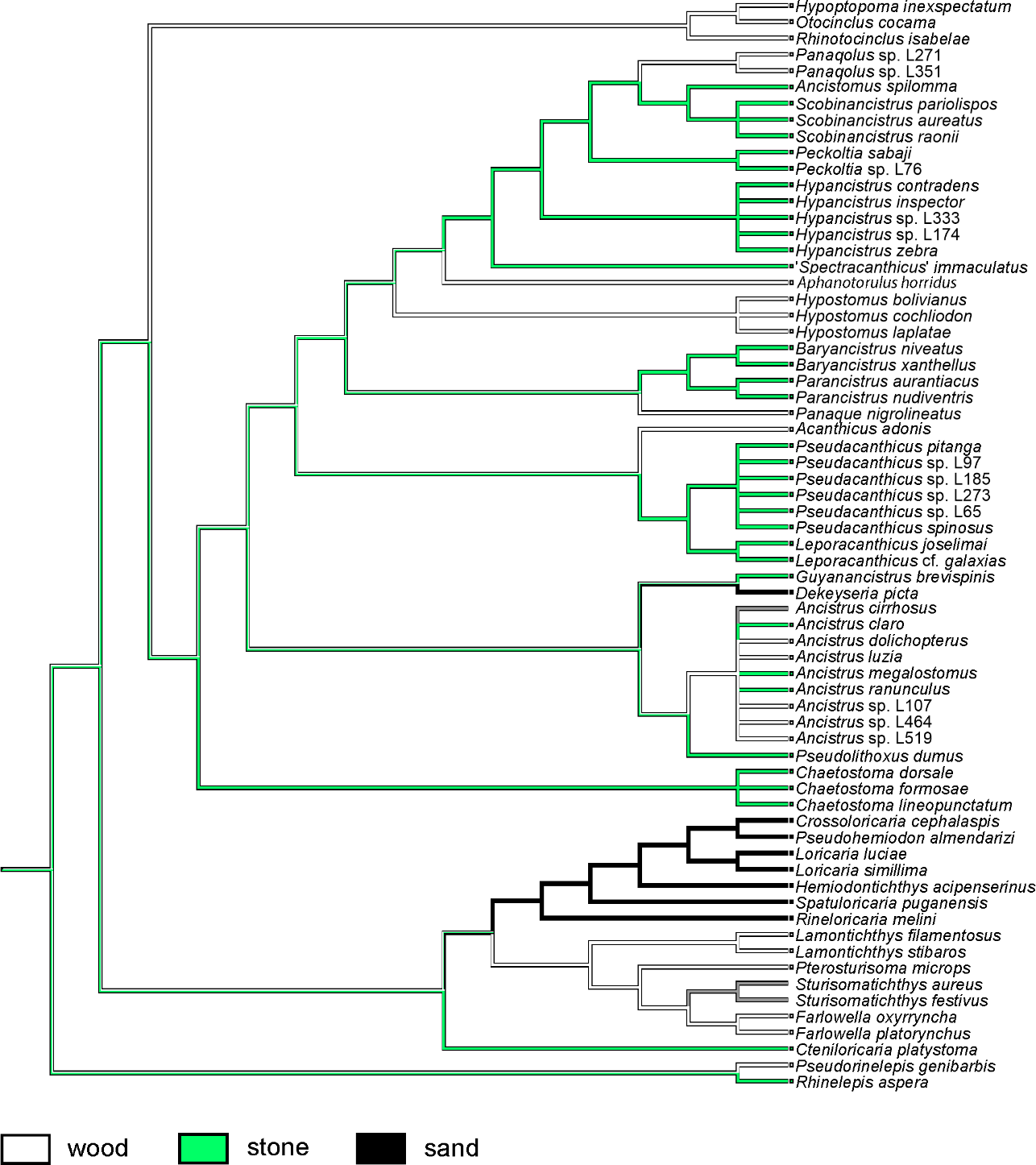
**

**Supplementary Figure 4. Reconstruction of ancestral substrate type (grey: habitat unknown or variable).**

**
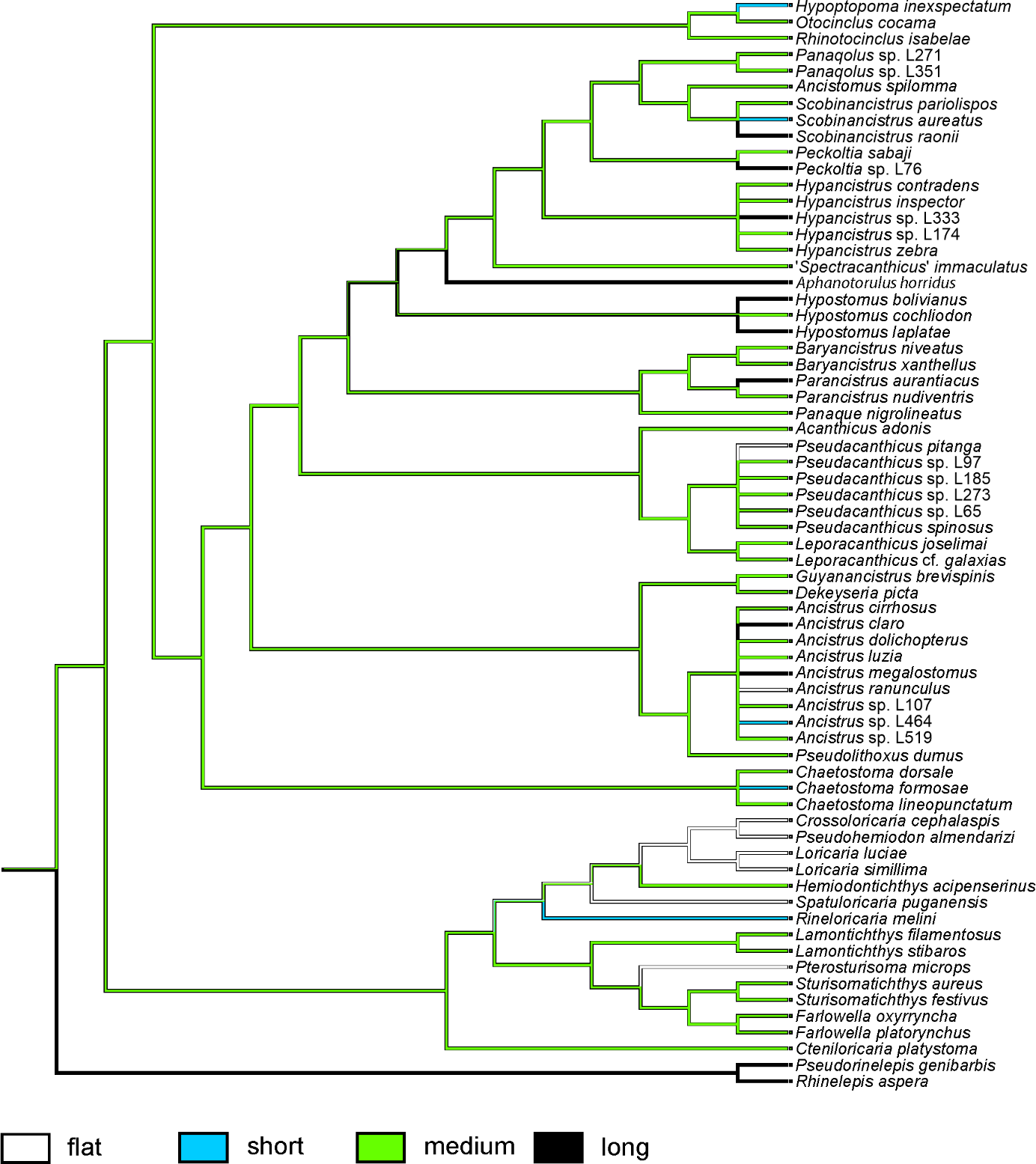
**

**Supplementary Figure 5. Reconstruction of ancestral papilla types.**

**
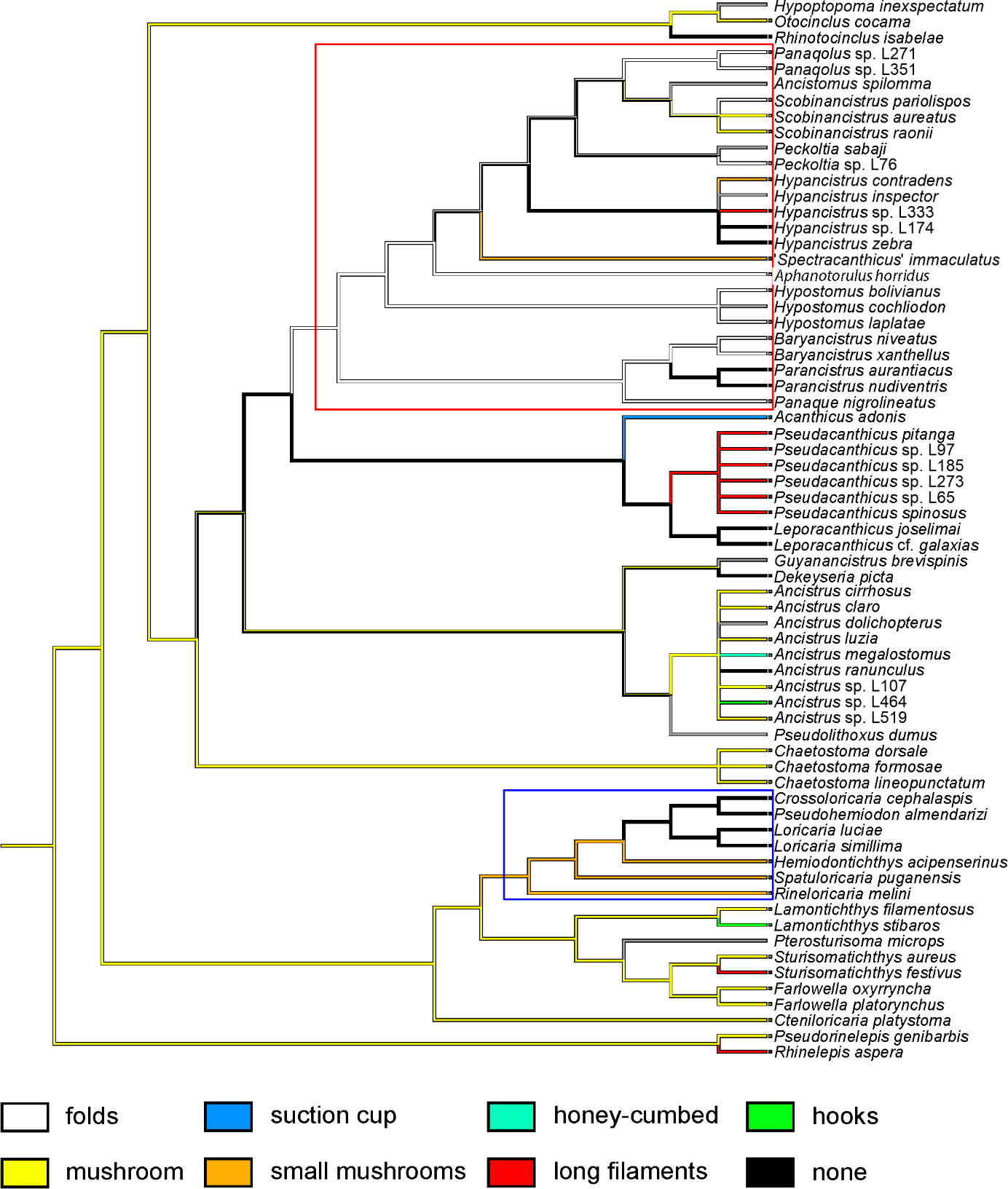
**

**Supplementary Figure 6. Reconstruction of ancestral unculi types (grey: unculi type unknown).**

**
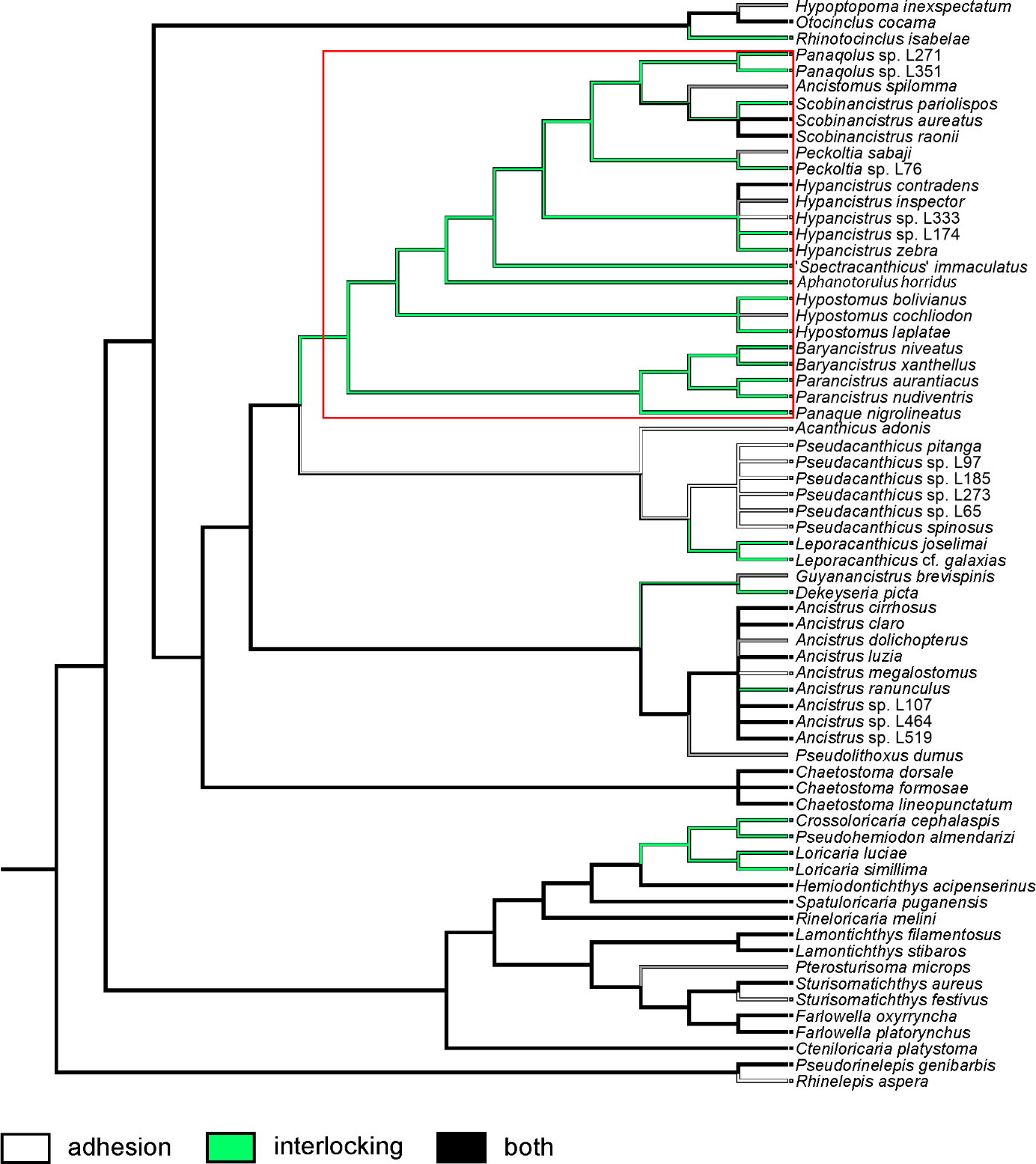
**

**Supplementary Figure 7. Reconstruction of ancestral interaction types (grey: unculi type unknown).**

**Supplementary** **Table 1.** For each species: ecological data (current and preferred substrate), results from morphological study (papillae and unculi types), inferred type of interaction between organism and substrate, and data on localities. N, number of specimens studied.

| **Group** | **Species** | **L number** | **Current** | **Substrate** | **Papillae** | **Unculi** | **Interaction type** | **Locality** | **GPS coordinates** | **N** |
| --- | --- | --- | --- | --- | --- | --- | --- | --- | --- | --- |
| Hypoptopomatinae | *Hypoptopoma inexspectatum* | LDA13 | Medium | Wood | Short | No data | No data | Río Paraguay, Argentina | 26°12'31.79"S,  58°7'27.86"W | 2 |
| Hypoptopomatinae | *Otocinclus cocama* |  | Medium | Wood | Medium | Mushroom | Interlocking and adhesion | Río Ucayali, Peru | 4°55'S,  73°43'W | 1 |
| Hypoptopomatinae | *Rhinotocinclus isabelae* |  | Medium | Wood | Medium | None | Interlocking | Río Nanay, Peru | 03°48'15"S,  73°23'53"W | 2 |
| Hypostominae | *Acanthicus adonis* |  | Medium | Wood | Medium | Suction cup | Adhesion | Rio Tocantins, Brazil | 2°14'39.23"S,  49°29'55.44"W | 2 |
| Hypostominae | *Ancistomus spilomma* | L12 | Strong | Stone | Medium | No data | No data | Rio Xingu, Brazil | 3°44'18.40"S,  52°31'33.36"W | 2 |
| Hypostominae | *Ancistrus cirrhosus* |  | No data | No data | Medium | Mushroom | Interlocking and adhesion | Río Paraguay drainage, Paraguay | 25°00'11"S,  57°36'58"W | 5 |
| Hypostominae | *Ancistrus claro* | LDA008 | Strong | Stone | Long | Mushroom | Interlocking and adhesion | Rio Paraguay, Brazil | 15°27'49.13"S,  55°58'53.30"W | 2 |
| Hypostominae | *Ancistrus dolichopterus* | L183 | Medium | Wood | Medium | No data | No data | Rio Negro, Brazil | 3°9'48.88"S,  60°3'1.05"W | 3 |
| Hypostominae | *Ancistrus luzia* | L159 | Medium | Wood | Medium | Mushroom | Interlocking and adhesion | Rio Xingu, Brazil | 09°47’15"S,  54°57’33"W | 2 |
| Hypostominae | *Ancistrus megalostomus* |  | Strong | Stone | Long | Honey-combed | Adhesion | Río Beni, Bolivia | 17°0'29.05"S,  65°27'21.45"W | 4 |
| Hypostominae | *Ancistrus ranunculus* | L34 | Strong | Stone | Flat | None | Interlocking | Rio Xingu, Brazil | 3°44'18.40"S,  52°31'33.36"W | 3 |
| Hypostominae | *Ancistrus* sp. | L107 | Medium | Wood | Medium | Mushroom | Interlocking and adhesion | Rio Negro, Brazil | 0°28'0.63"S,  62°54'58.69"W | 2 |
| Hypostominae | *Ancistrus* sp. | L464 | Medium | Wood | Short | Hooks | Interlocking and adhesion | Río Mamoré, Bolivia | 17°23'27.36"S,  63°31'30.17"W | 4 |
| Hypostominae | *Ancistrus* sp. | L519 | Medium | Wood | Medium | Mushroom | Interlocking and adhesion | Río Itenéz, Bolivia | 12°29'51.13"S  64°6'40.10"W | 4 |
| Hypostominae | *Aphanotorulus horridus* |  | Medium | Wood | Long | Folds | Interlocking | Río Beni, Bolivia | 16°54'3.50"S,  65°24'35.44"W | 1 |
| Hypostominae | *Baryancistrus* *niveatus* | L142 | Strong | Stone | Medium | Folds | Interlocking | Rio Tapajós, Brazil | 4°17'27.75"S,  55°59'8.89"W | 1 |
| Hypostominae | *Baryancistrus xanthellus* | L177; L81 | Strong | Stone | Medium | Folds | Interlocking | Rio Xingu, Brazil | 3°27'07"S,  51°41'53"W | 3 |
| Hypostominae | *Chaetostoma dorsale* | L443 | Strong | Stone | Medium | Mushroom | Interlocking and adhesion | Río Orinoco, Venezuela | 8°35'18.85"N,  71°9'34.63"W | 2 |
| Hypostominae | *Chaetostoma formosae* | L444 | Strong | Stone | Short | Mushroom | Interlocking and adhesion | Río Orinoco, Venezuela | 4°20'11.27"N,  72°45'53.96"W | 3 |
| Hypostominae | *Chaetostoma lineopunctatum* | L276 | Strong | Stone | Medium | Mushroom | Interlocking and adhesion | Río Ucayali, Peru | 10°23'24.32"S,  74°54'5.40"W | 3 |
| Hypostominae | *Dekeyseria picta* | L168 | Medium | Sand/Mudd | Medium | None | Interlocking | Río Atabapo, Colombia | 3°53'58.97"N,  67°40'13.47"W | 1 |
| Hypostominae | *Guyanancistrus brevispinis* |  | Medium | Stone | Medium | No data | No data | Nickerie-River, Surinam | 05˚24'28.6"N 53˚34'53.6"W | 1 |
| Hypostominae | *Hypancistrus contradens* |  | Medium | Stone | Medium | Small mushrooms | Interlocking and adhesion | Río Orinoco, Venezuela | 4°04'50"N 66°51'31"W | 2 |
| Hypostominae | *Hypancistrus inspector* |  | Strong | Stone | Medium | No data | No data | Rio Negro, Brazil | 1°58'N,  67°05'W | 3 |
| Hypostominae | *Hypancistrus* sp. | L333 | Strong | Stone | Long | Long filaments | Adhesion | Rio Xingu, Brazil | 1°52'7.18"S,  52°12'41.86"W | 3 |
| Hypostominae | *Hypancistrus* sp. | L174 | Strong | Stone | Medium | None | Interlocking | Rio Xingu, Brazil | 3°9'2.41"S,  51°39'56.89"W | 2 |
| Hypostominae | *Hypancistrus zebra* | L46 | Strong | Stone | Medium | None | Interlocking | Rio Xingu, Brazil | 3°44'18.40"S,  52°31'33.36"W | 2 |
| Hypostominae | *Hypostomus bolivianus* |  | Medium | Wood | Long | Folds | Interlocking | Río Beni, Bolivia | 11°20'48.16"S,  66°14'4.53"W | 1 |
| Hypostominae | *Hypostomus cochliodon* | LDA051 | Medium | Wood | Medium | No data | No data | Rio Cuiabá, Brazil. | 15°47'49.38"S,  56°7'33.12"W | 1 |
| Hypostominae | *Hypostomus laplatae* |  | Medium | Wood | Long | Folds | Interlocking | Río de la Plata, Argentina | 34°51'14.45"S,  57°52'42.74"W | 1 |
| Hypostominae | *Leporacanthicus joselimai* | L264 | Medium | Stone | Medium | None | Interlocking | Río Tapajós, Brazil | 4°17'27.75"S,  55°59'8.89"W | 2 |
| Hypostominae | *Leporacanthicus* cf. *galaxias* | L240 | Medium | Stone | Medium | None | Interlocking | Río Orinoco, Venezuela | 5°36'4.53"N,  67°37'21.25"W | 2 |
| Hypostominae | *Panaqolus* sp. | L271 | Medium | Wood | Medium | Folds | Interlocking | Rio Tapajós, Brazil | 4°37'10.21"S,  56°17'31.81"W | 2 |
| Hypostominae | *Panaqolus* sp. | L351 | Medium | Wood | Medium | Folds | Interlocking | Río Huallaga, Peru | 9°19'14.52"S,  76°0'5.06"W | 3 |
| Hypostominae | *Panaque nigrolineatus* | L190 | Medium | Wood | Medium | Folds | Interlocking | Río Apure, Venezuela | 8°50'9.79"N,  67°26'48.12"W | 1 |
| Hypostominae | *Parancistrus aurantiacus* |  | Strong | Stone | Long | None | Interlocking | Rio Tocantins, Brazil | 3°42'43.66"S,  49°39'47.72"W | 2 |
| Hypostominae | *Parancistrus nudiventris* | L31; L258; L300 | Strong | Stone | Medium | None | Interlocking | Rio Xingu, Brazil | 3°23'19"S,  51°43'24"W | 3 |
| Hypostominae | *Peckoltia* *sabaji* | L75 | Strong | Stone | Medium | No data | No data | Rio Xingu, Brazil | 3°44'18.40"S,  52°31'33.36"W | 2 |
| Hypostominae | *Peckoltia* sp. | L76 | Medium | Stone | Long | Folds | Interlocking | Rio Amazonas, Brazil | 1°52'33.36"S,  50°52'21.44"W | 2 |
| Hypostominae | *Pseudacanthicus pitanga* | L24 | Strong | Stone | Flat | Long filaments | Adhesion | Rio Araguaia, Brazil | 5°19'33.22"S,  49°0'4.64"W | 1 |
| Hypostominae | *Pseudacanthicus* sp. | L97 | Medium | Stone | Medium | Long filaments | Adhesion | Rio Amazonas, Brazil | 2°52'25.69"S,  54°20'24.33"W | 2 |
| Hypostominae | *Pseudacanthicus* sp. | L185 | Strong | Stone | Medium | Long filaments | Adhesion | Rio Xingu, Brazil | 3°2'0.49"S,  51°51'1.41"W | 1 |
| Hypostominae | *Pseudacanthicus* sp. | L273 | Strong | Stone | Medium | Long filaments | Adhesion | Rio Tapajós, Brazil | 4°37'10.21"S,  56°17'31.81"W | 1 |
| Hypostominae | *Pseudacanthicus* sp. | L65 | Medium | Stone | Medium | Long filaments | Adhesion | Rio Amazonas, Brazil | 3°12'25.47"S,  60°46'3.92"W | 1 |
| Hypostominae | *Pseudacanthicus spinosus* | L160 | Strong | Stone | Medium | Long filaments | Adhesion | Rio Amazonas, Brazil | 1°33'18.58"S,  48°9'43.92"W | 1 |
| Hypostominae | *Pseudolithoxus dumus* | L244 | Strong | Stone | Medium | No data | No data | Río Orinoco, Venezuela | 3°04'N,  66°28'W | 1 |
| Hypostominae | *Scobinancistrus* *pariolispos* | L48 | Strong | Stone | Medium | Folds | Interlocking | Rio Xingu, Brazil | 3°51'35.81"S,  52°50'24.96"W | 2 |
| Hypostominae | *Scobinancistrus aureatus* | L14 | Strong | Stone | Short | Mushroom | Interlocking and adhesion | Rio Xingu, Brazil | 3°51'35.81"S,  52°50'24.96"W | 1 |
| Hypostominae | *Scobinancistrus* *raonii* | L82 | Strong | Stone | Long | Mushroom | Interlocking and adhesion | Rio Xingu, Brazil | 3°44'18.40"S,  52°31'33.36"W | 2 |
| Hypostominae | ‘*Spectracanthicus*’ *immaculatus* | L269 | Strong | Stone | Medium | None | Interlocking | Rio Tapajós, Brazil | 4°16’46.56"S,  55°59’5.77"W | 1 |
| Loricariinae | *Crossoloricaria cephalaspis* |  | Strong | Sand/Mudd | Flat | None | Interlocking | Río Magdalena, Colombia | 5°15'0.00"N,  74°49'60.00"W | 1 |
| Loricariinae | *Cteniloricaria platystoma* |  | Strong | Stone | Medium | Mushroom | Interlocking and adhesion | Corantijn River, Suriname | 2°1'4.75"N,  56°7'39.69"W | 1 |
| Loricariinae | *Farlowella oxyrryncha* |  | Medium | Wood | Medium | Mushroom | Interlocking and adhesion | Río Mamoré, Brazil | 11°27'51.23"S,  65°18'54.54"W | 1 |
| Loricariinae | *Farlowella platorynchus* |  | Medium | Wood | Medium | Mushroom | Interlocking and adhesion | Río Ucayali, Peru | 8°23'S,  74°32'W | 1 |
| Loricariinae | *Rineloricaria melini* |  | Medium | Sand/Mudd | Short | Small mushrooms | Interlocking and adhesion | Rio Negro, Brazil | 3°9'48.88"S,  60°3'1.05"W | 2 |
| Loricariinae | *Hemiodontichthys acipenserinus* |  | Medium | Sand/Mudd | Medium | Small mushrooms | Interlocking and adhesion | Rio Guaporé, Brazil | 12°29'38.55"S,  64°4'12.72"W | 3 |
| Loricariinae | *Lamontichthys filamentosus* |  | Strong | Wood | Medium | Mushroom | Interlocking and adhesion | Rio Jurua, Brazil | 7°25'58.65"S,  70°2'44.42"W | 2 |
| Loricariinae | *Lamontichthys stibaros* |  | Strong | Wood | Medium | Hooks | Interlocking and adhesion | Río Pastaza, Ecuador | 2°25'S,  76°38'W | 2 |
| Loricariinae | *Loricaria luciae* |  | Medium | Sand/Mudd | Flat | None | Interlocking | Río Paraná, Argentina | 27°25’6.94"S,  58°45’27.47"W | 1 |
| Loricariinae | *Loricaria simillima* |  | Medium | Sand/Mudd | Flat | None | Interlocking | Río Pastaza, Ecuador | 1°35'28.25"S,  77°44'29.05"W | 1 |
| Loricariinae | *Pseudohemiodon almendarizi* |  | Strong | Sand/Mudd | Flat | None | Interlocking | Río Napo, Ecuador | 0°3'57.51"N,  76°51'13.05"W | 2 |
| Loricariinae | *Pterosturisoma microps* |  | Strong | Wood | Flat | No data | No data | Río Amazonas, Peru | 3°43'20.36"S,  73°17'0.38"W | 2 |
| Loricariinae | *Spatuloricaria puganensis* |  | Medium | Sand/Mudd | Flat | Small mushrooms | Interlocking and adhesion | Río Marañon, Peru | 5°3'28.75"S,  75°59'59.13"W | 2 |
| Loricariinae | *Sturisomatichthys aureus* |  | Medium | Wood & Stone | Medium | Mushroom | Interlocking and adhesion | Río Magdalena, Colombia | 9°34'23.24"N,  73°19'24.62"W | 2 |
| Loricariinae | *Sturisomatichthys festivus* |  | Medium | Wood & Stone | Medium | Long filaments | Adhesion | Río Maracaíbo, Venezuela | 9°39'8.55"N,  70°26'38.97"W | 2 |
| Rhinelepinae | *Pseudorinelepis* *genibarbis* | L152; L 95 | Slow | Wood | Long | Mushroom | Interlocking and adhesion | Rio Negro, Brazil | 3°9'48.88"S, 60°3'1.05"W;  3°4'46.78"S, 61°29'17.86"W | 2 |
| Rhinelepinae | *Rhinelepis aspera* |  | Strong | Wood | Long | Long filaments | Adhesion | Rio São Francisco, Brazil | 15°29’37"S,  44°21’25"W | 2 |


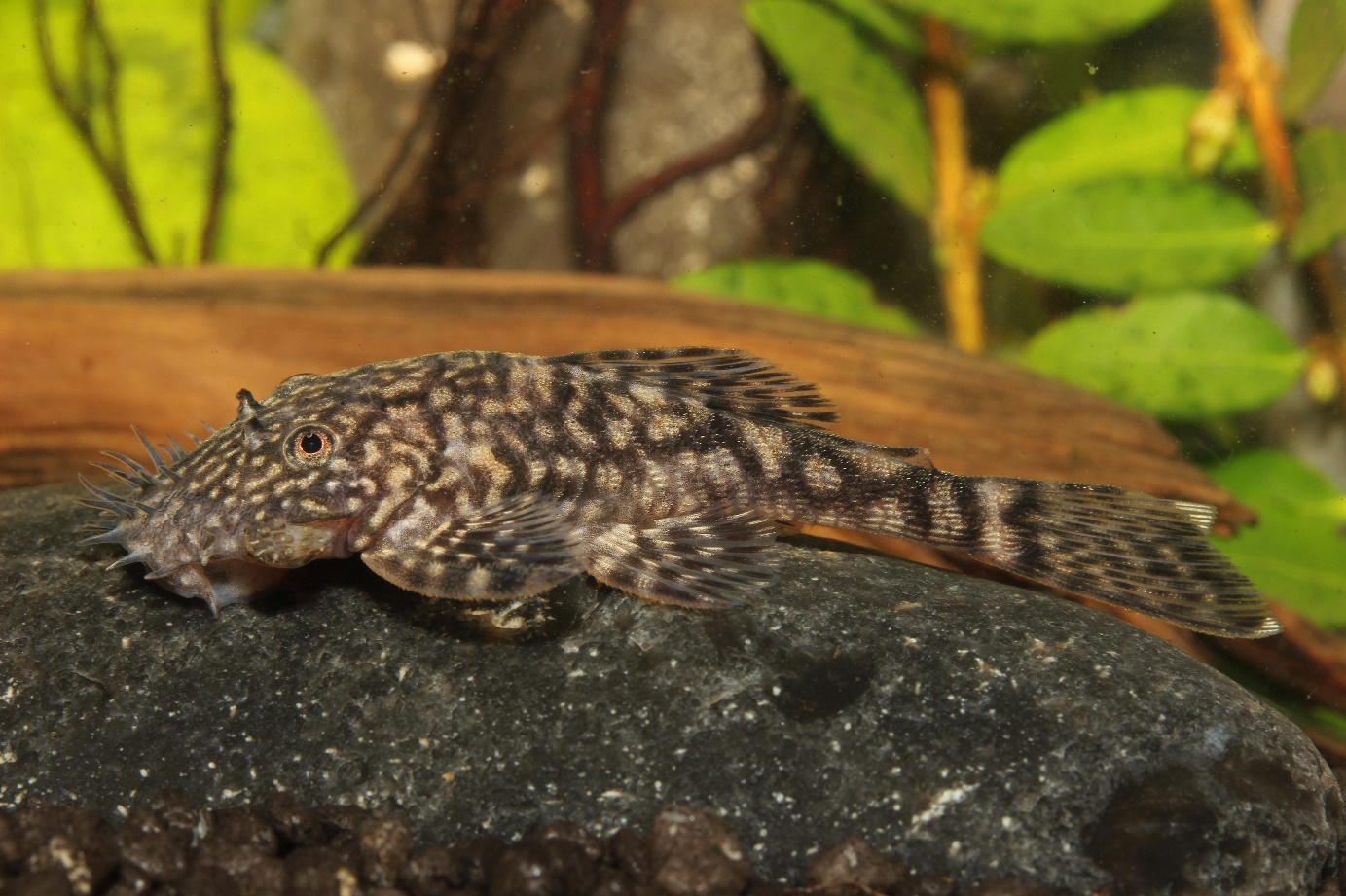

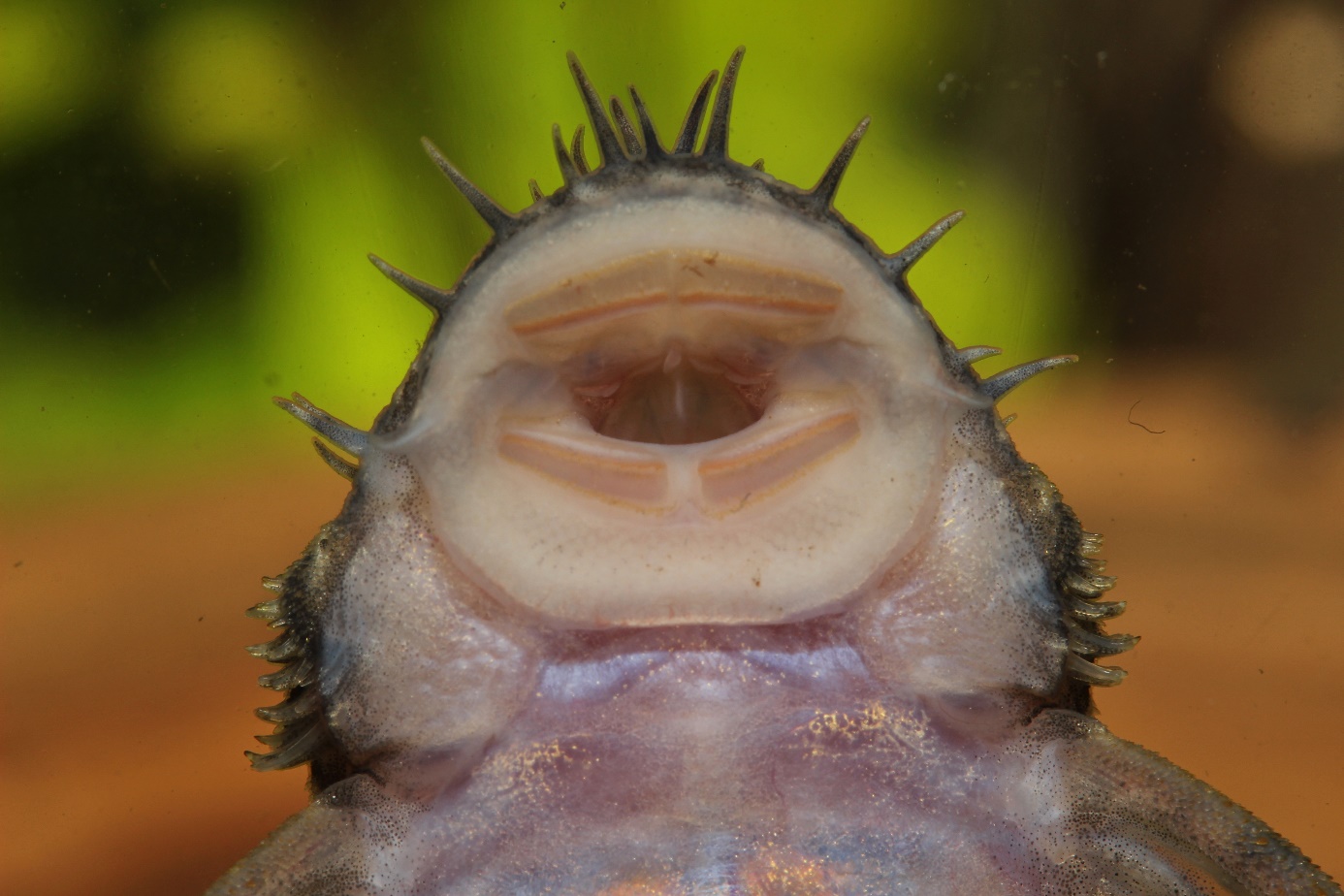


**Supplementary Figure 8. Habitus images of *Ancistrus claro*.**


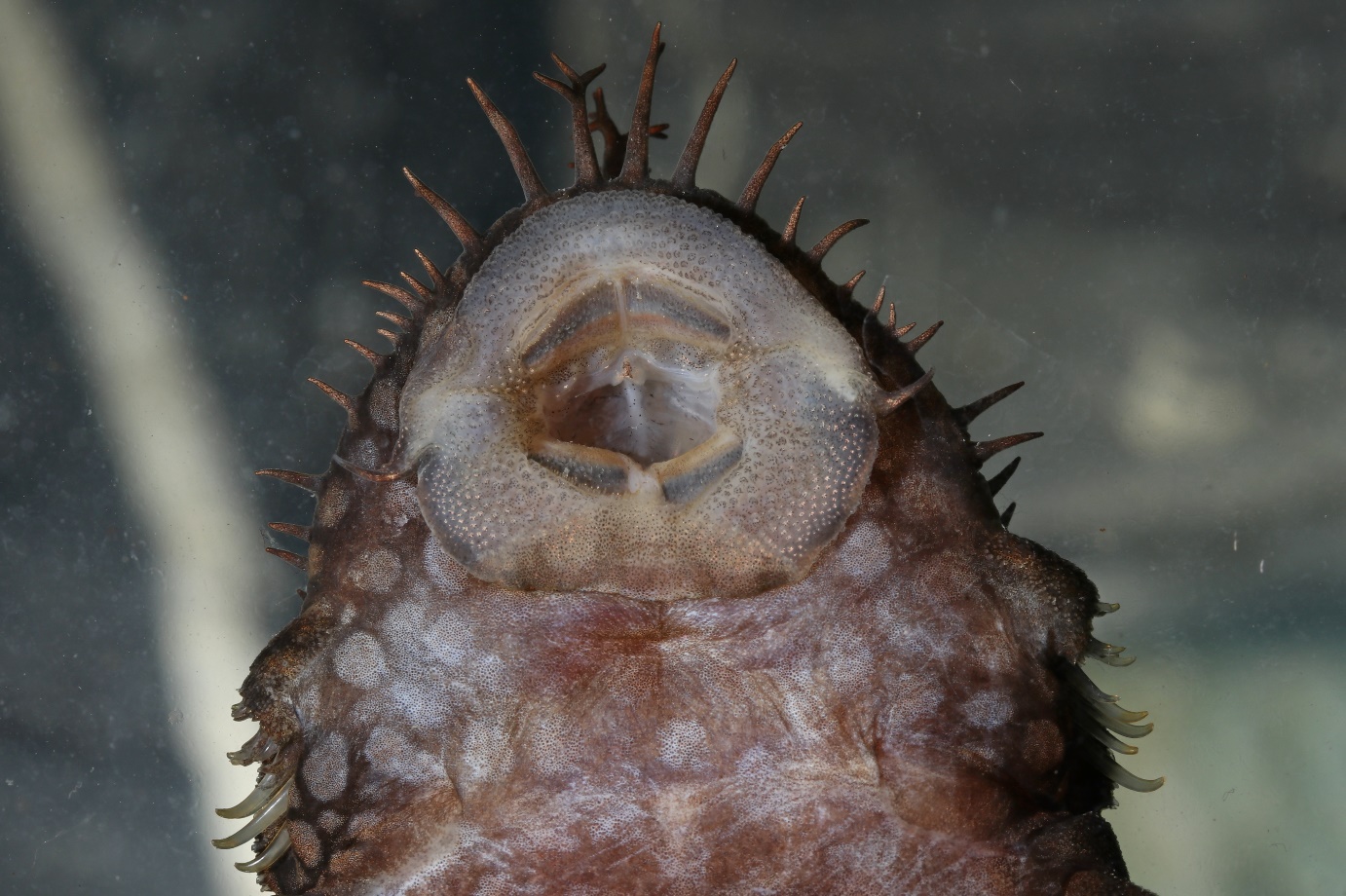

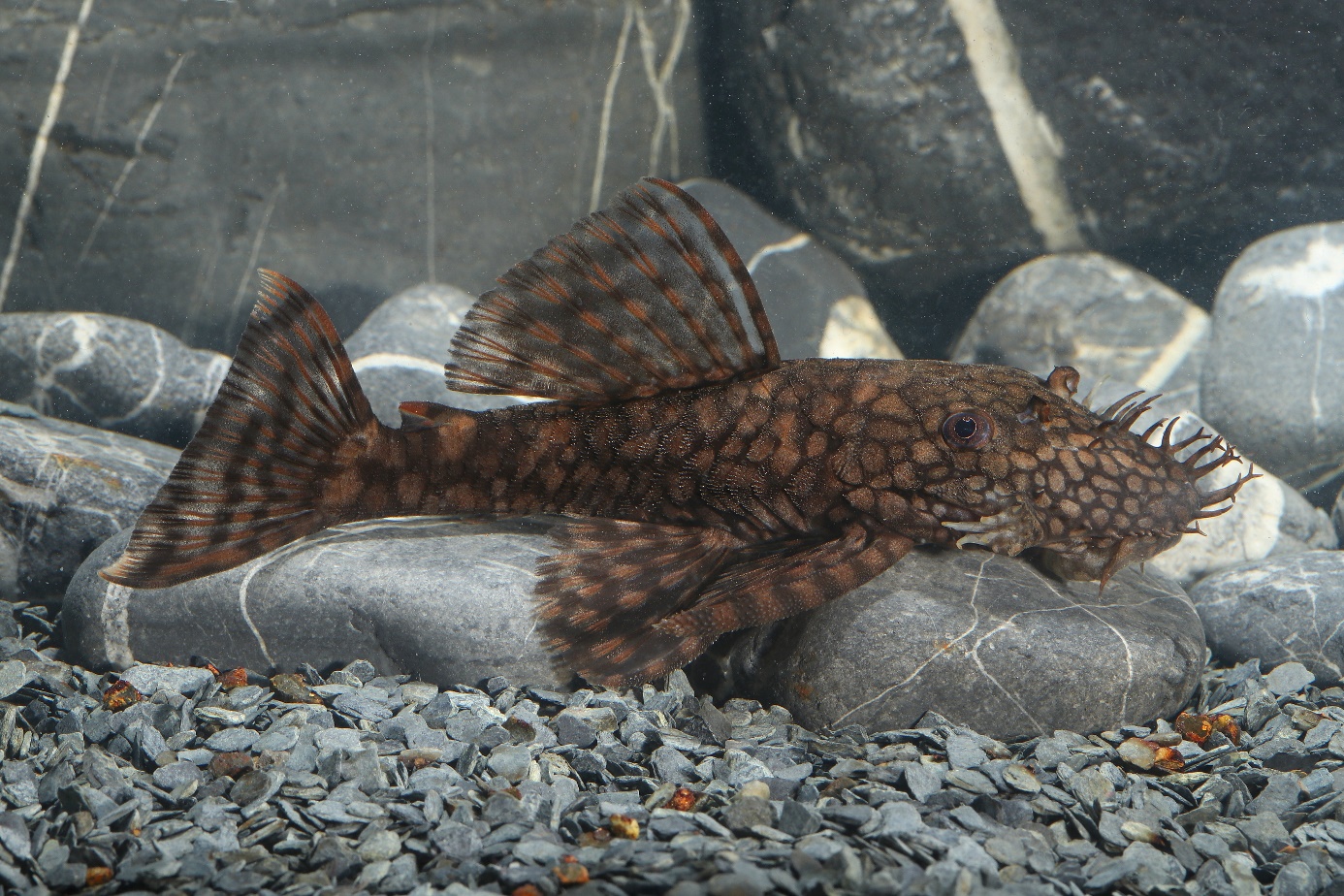


**Supplementary Figure 9. Habitus images of *Ancistrus* sp. L464.**


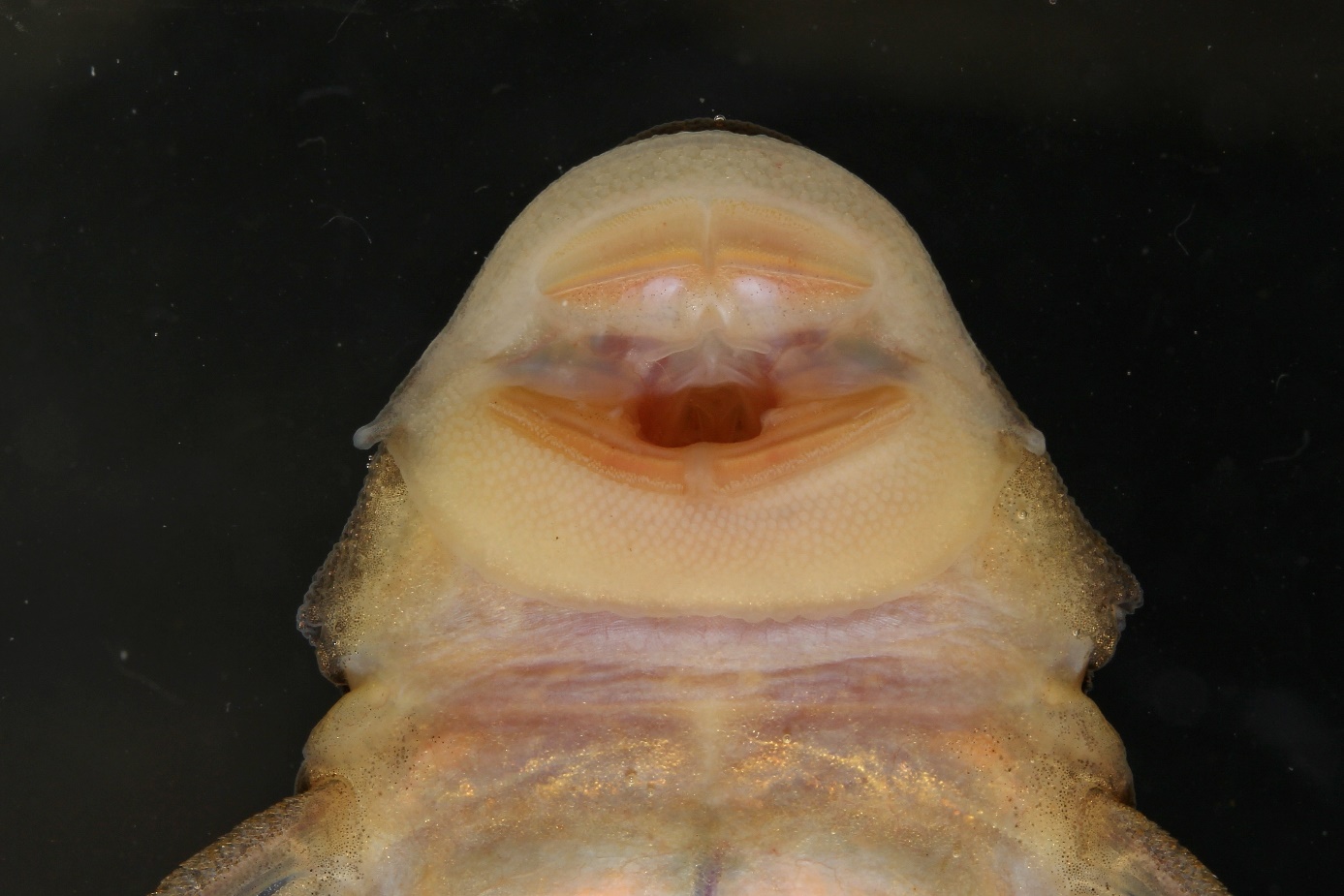

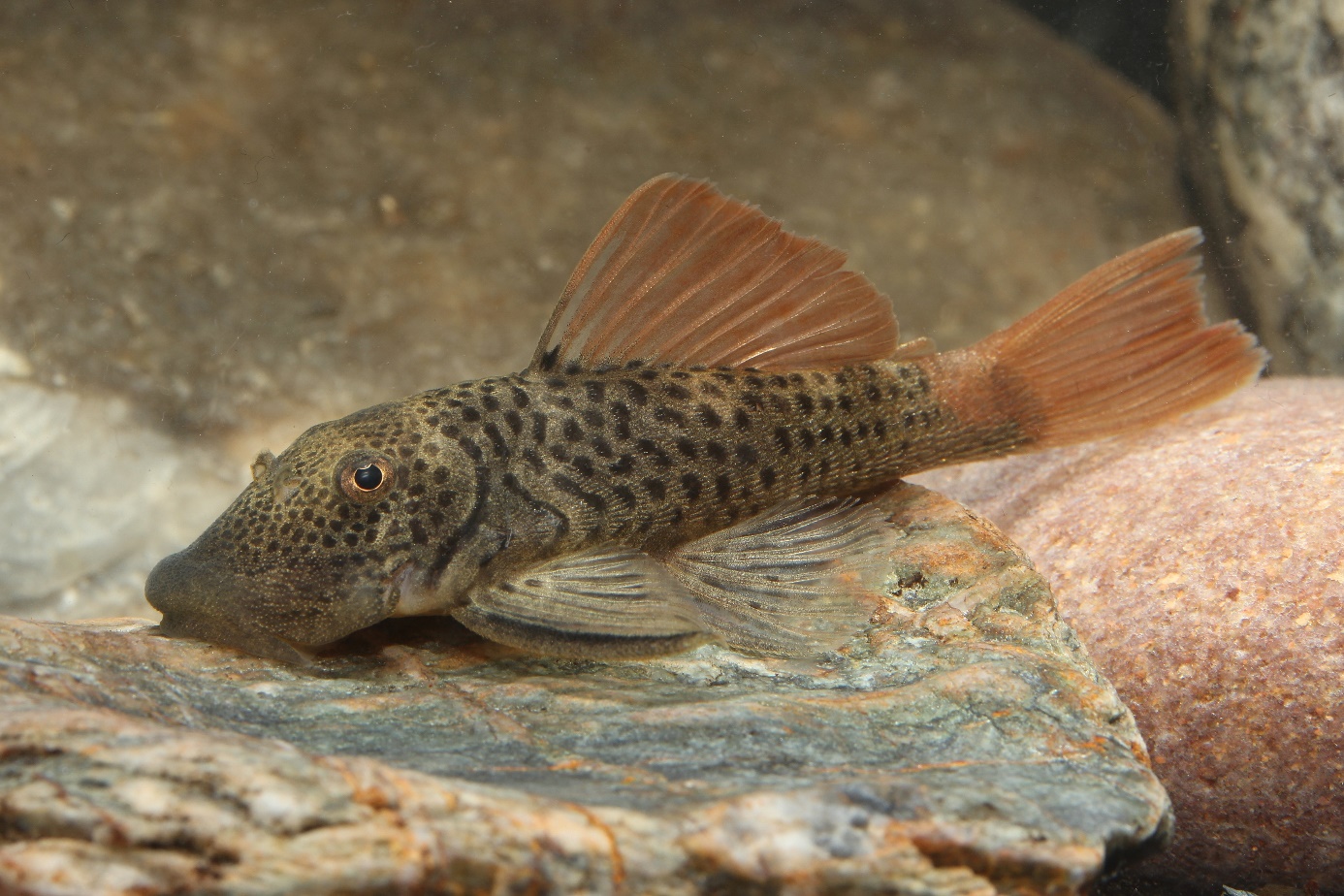


**Supplementary Figure 10. Habitus images of *Chaetostoma lineopunctatum*.**


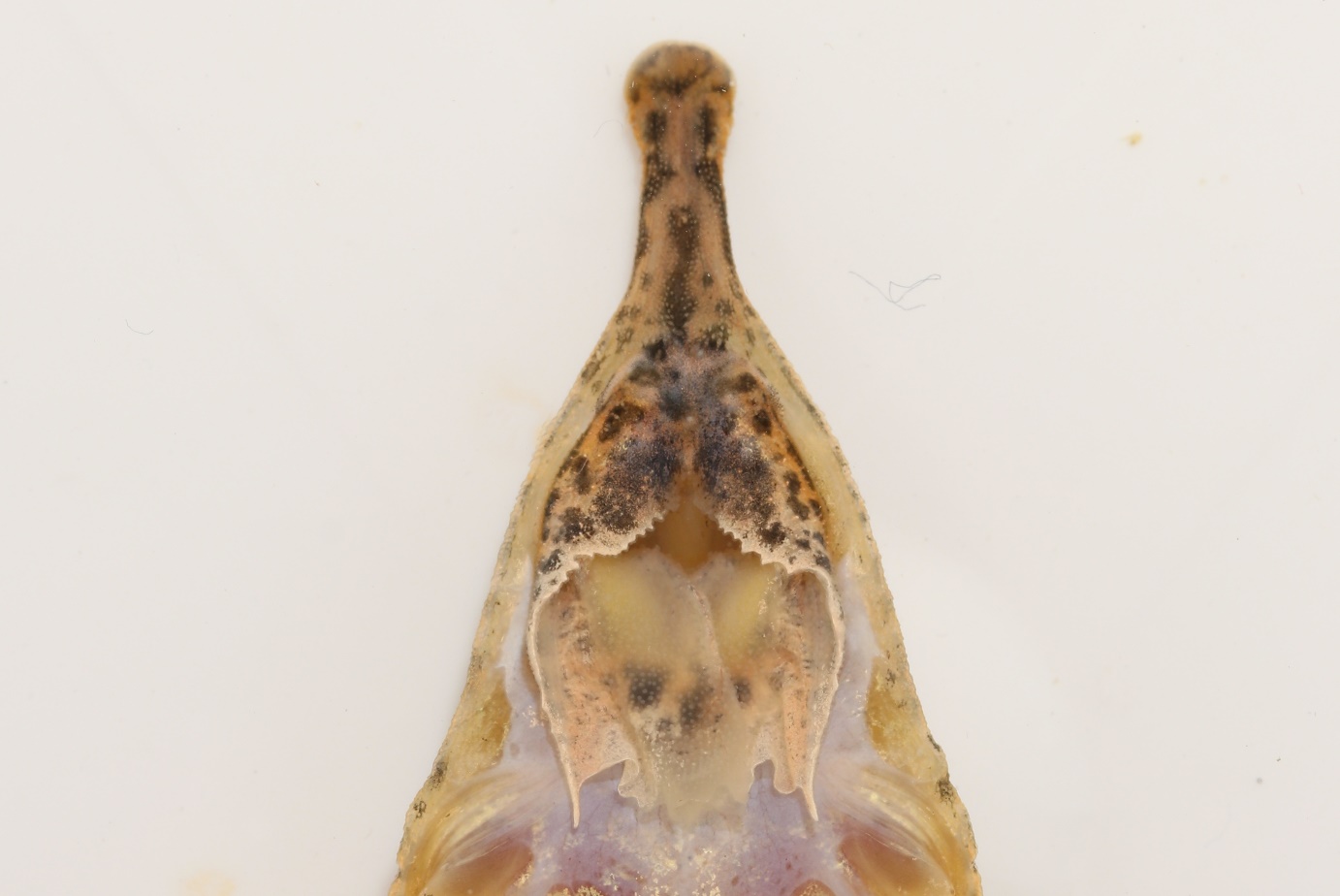

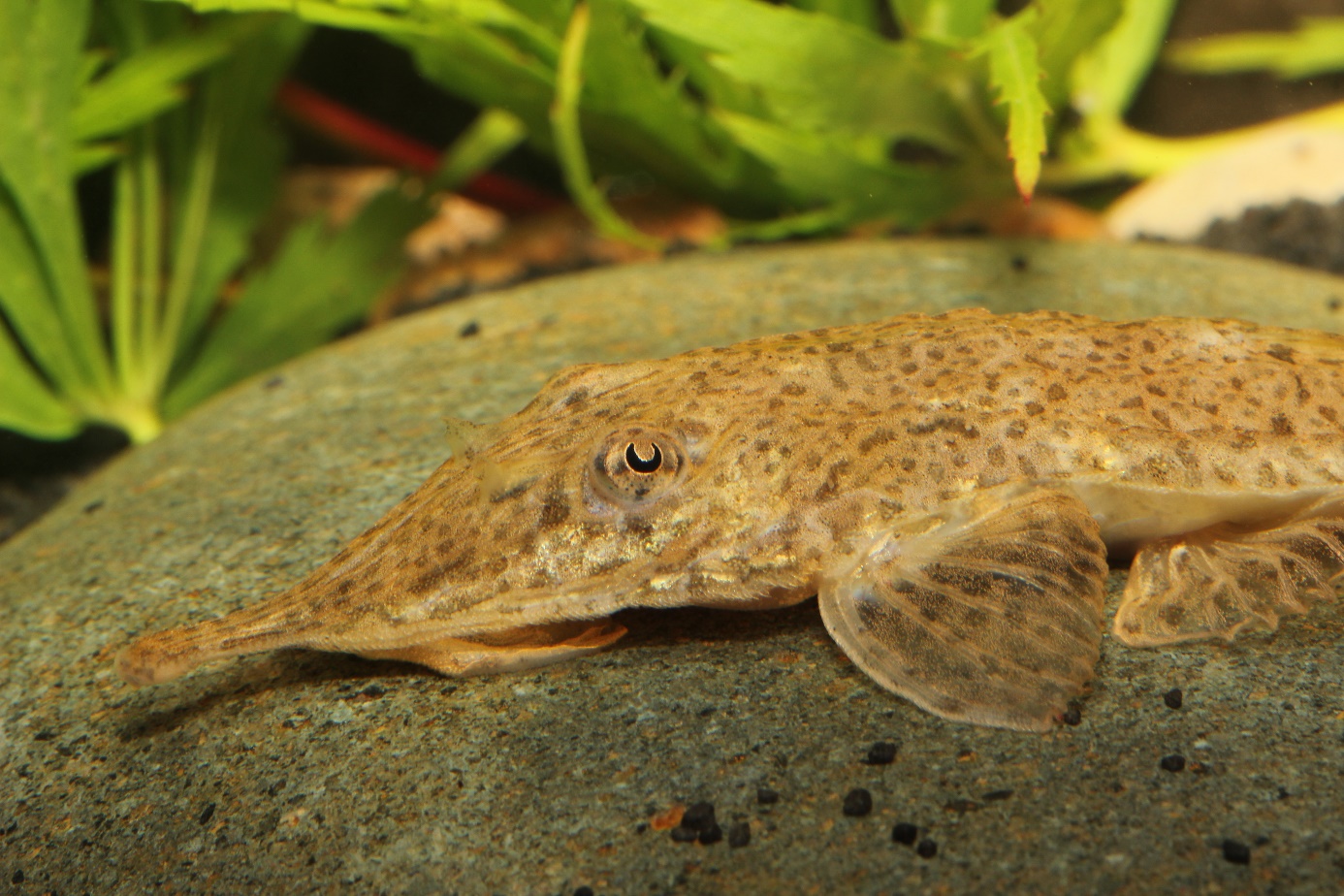


**Supplementary Figure 11. Habitus images of *Hemiodontichthys acipenserinus*.**


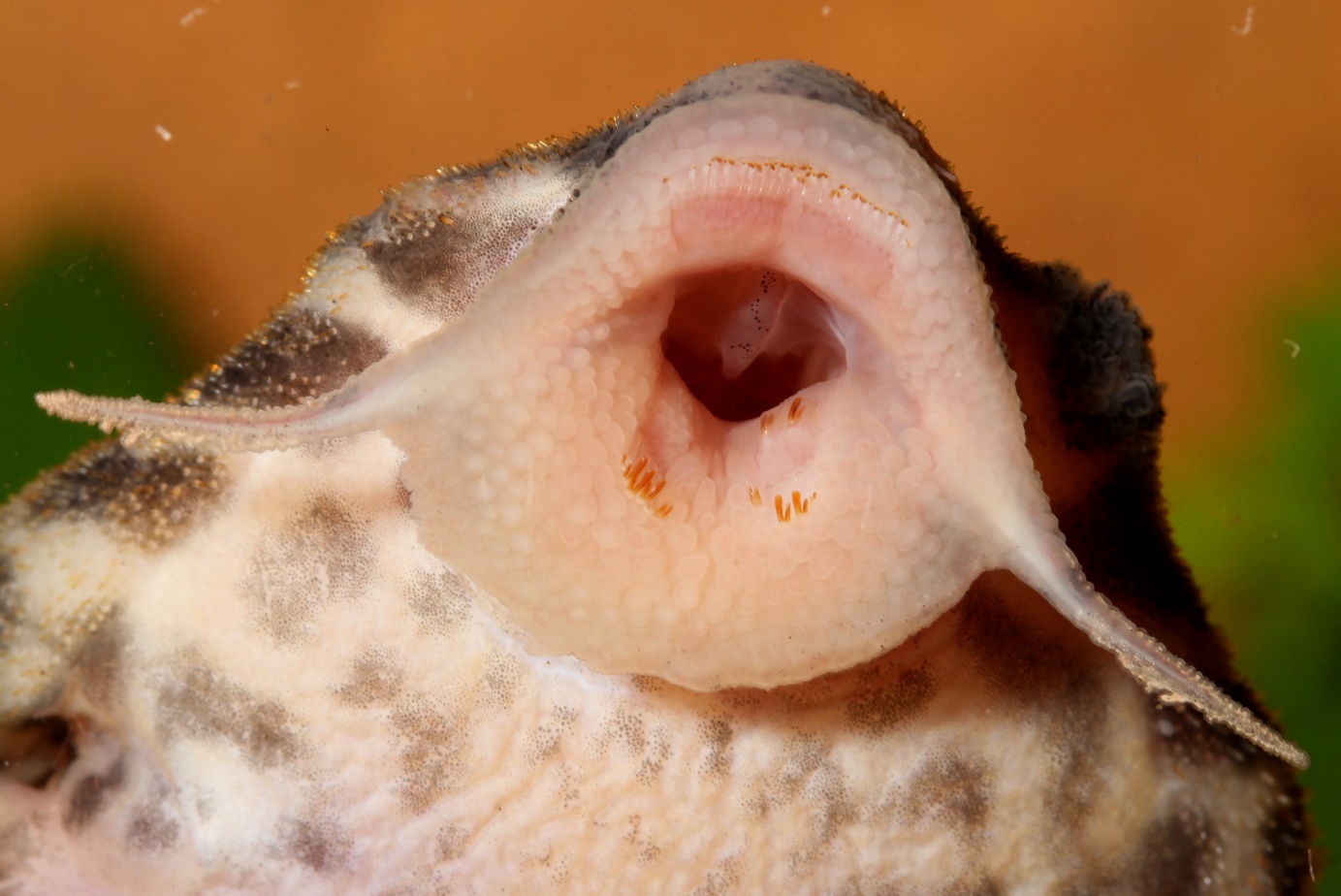

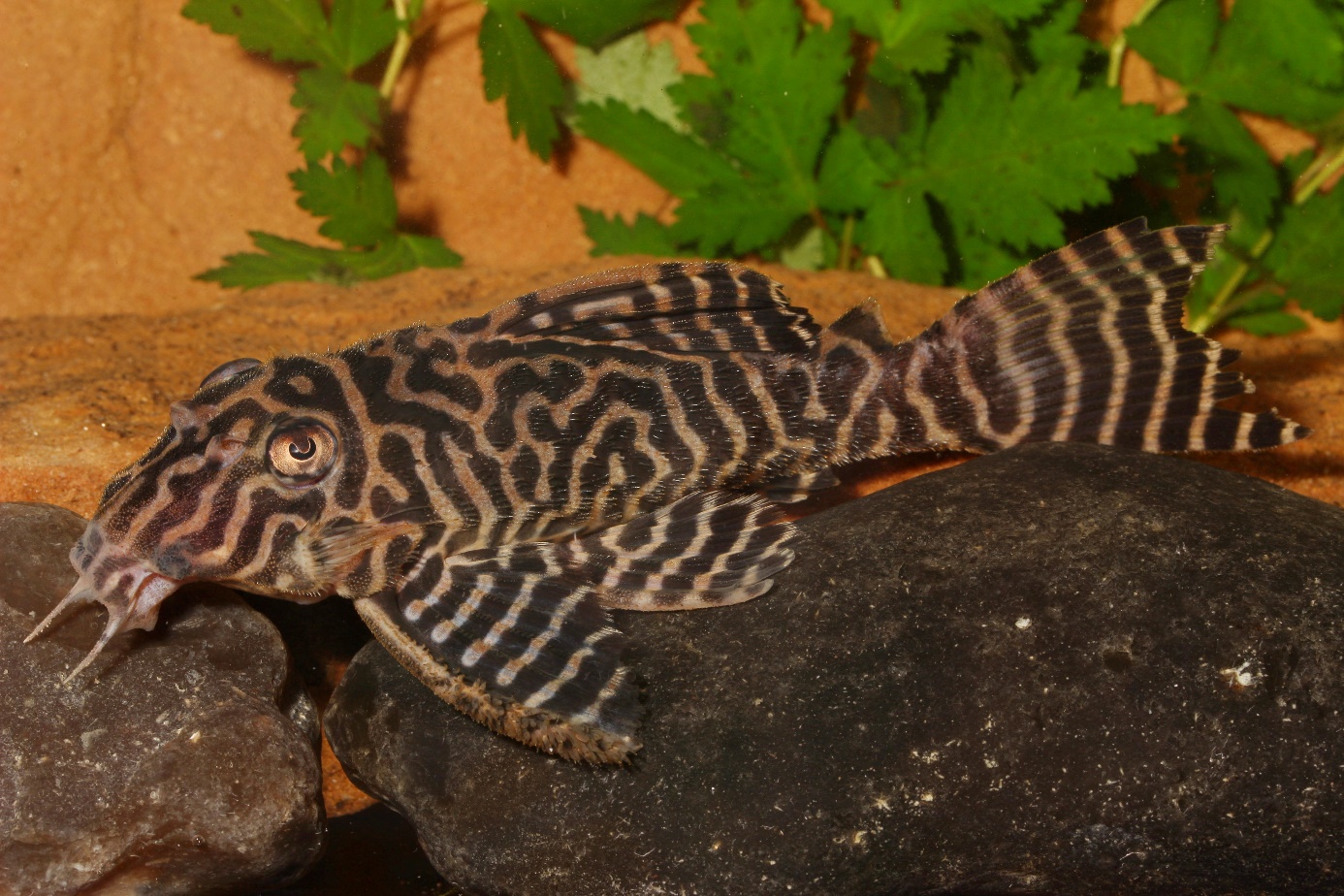


**Supplementary Figure 12. Habitus images of *Hypancistrus* sp. L333.**


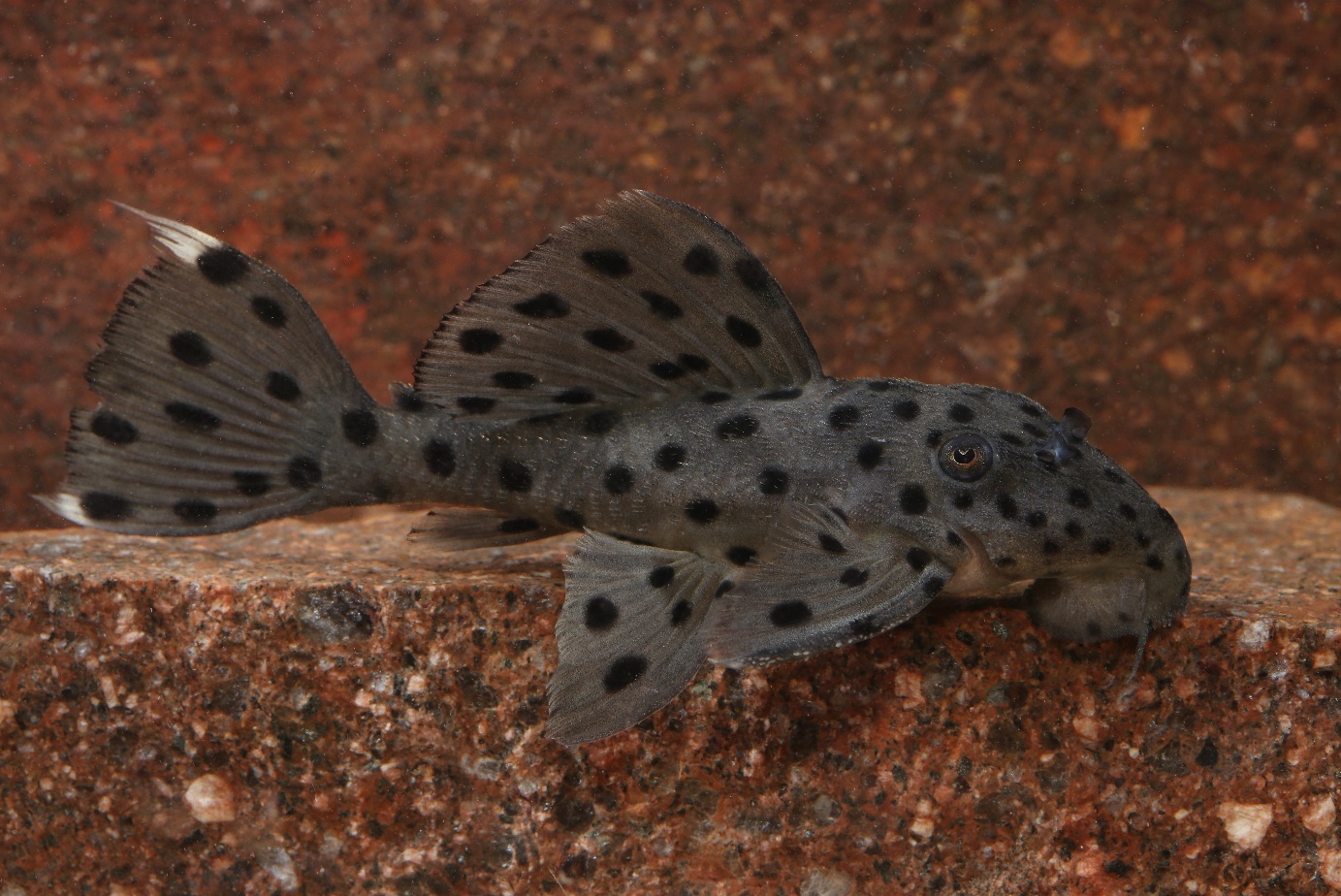

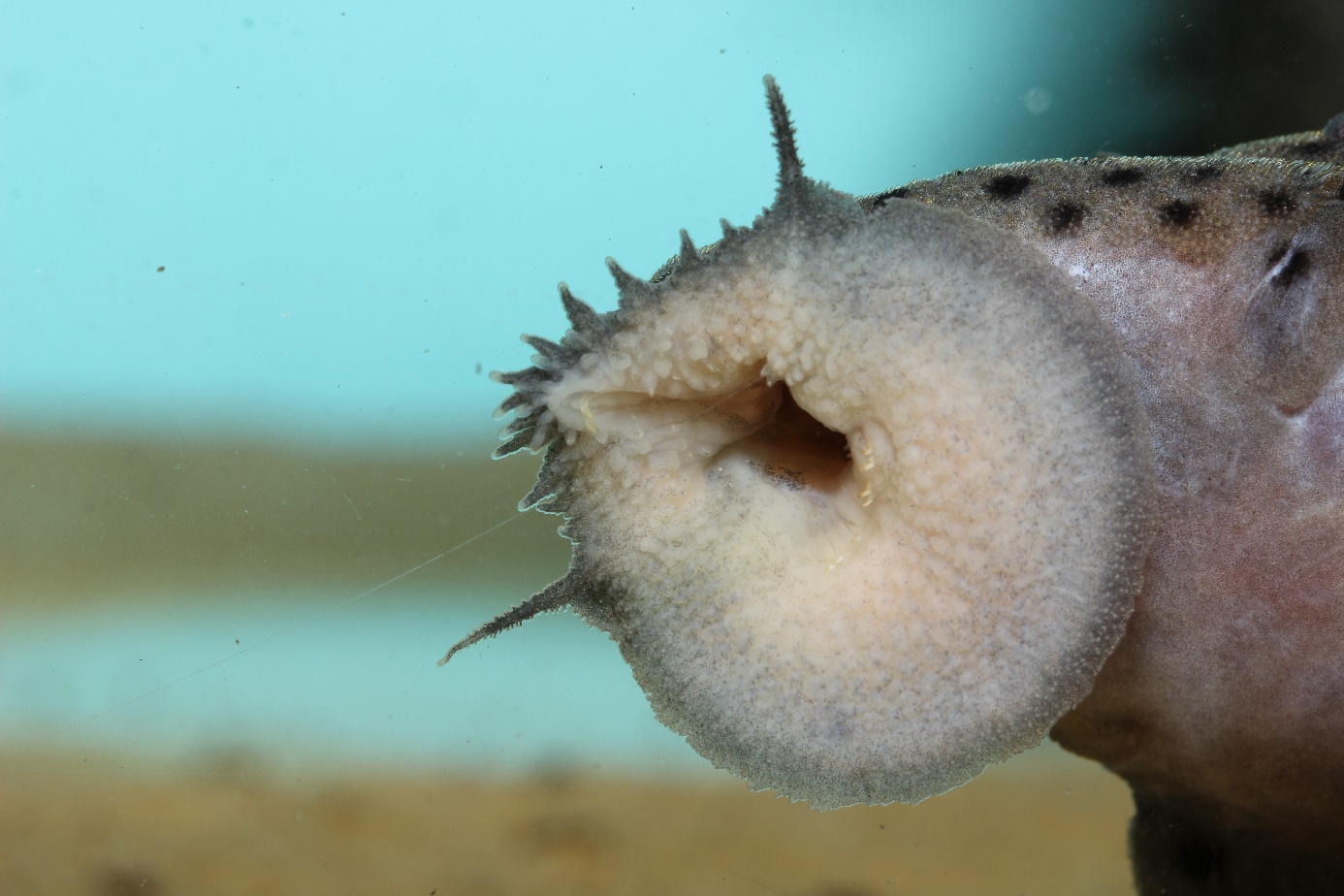


**Supplementary Figure 13. Habitus images of *Leporacanthicus joselimai*.**


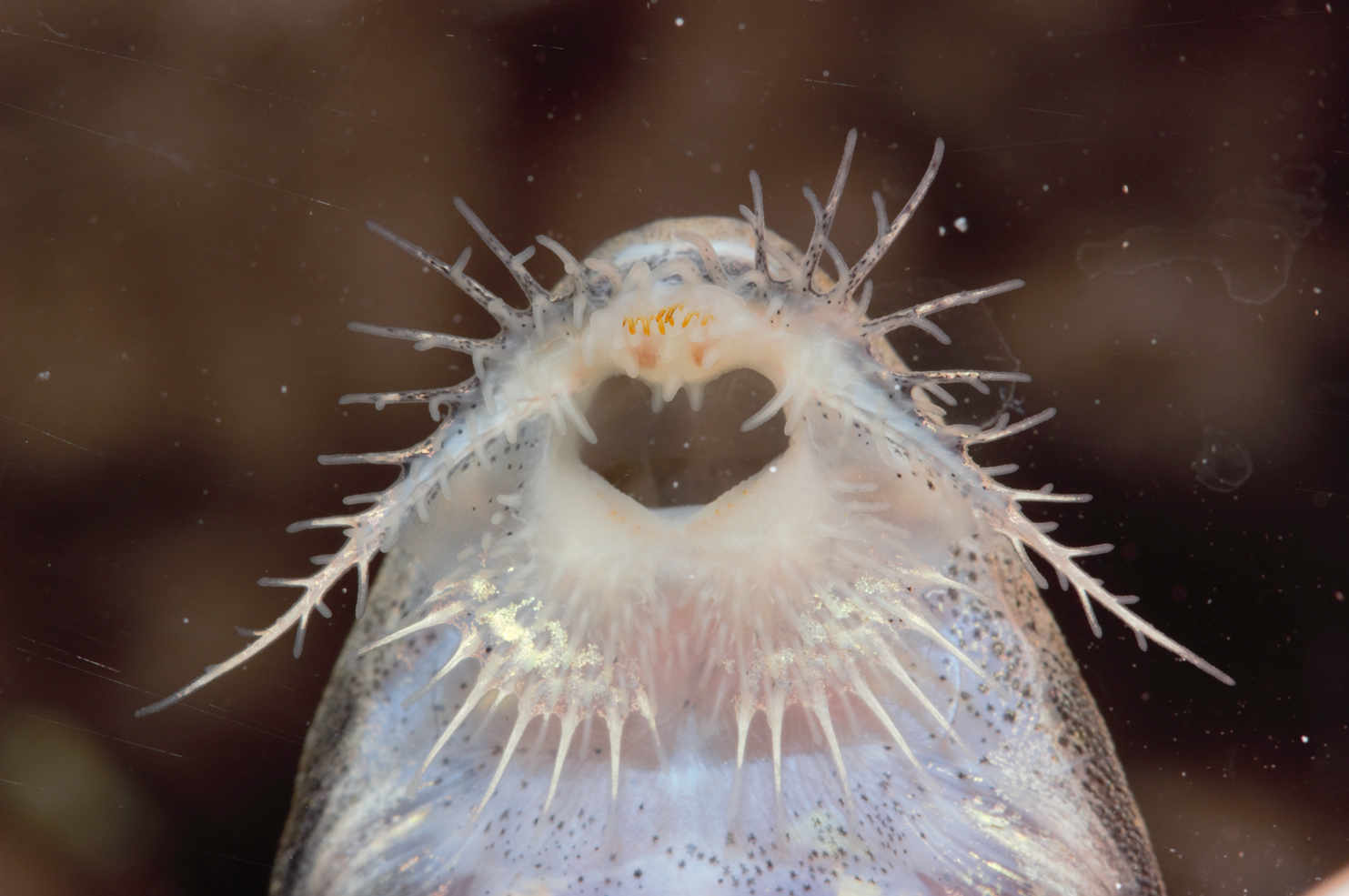


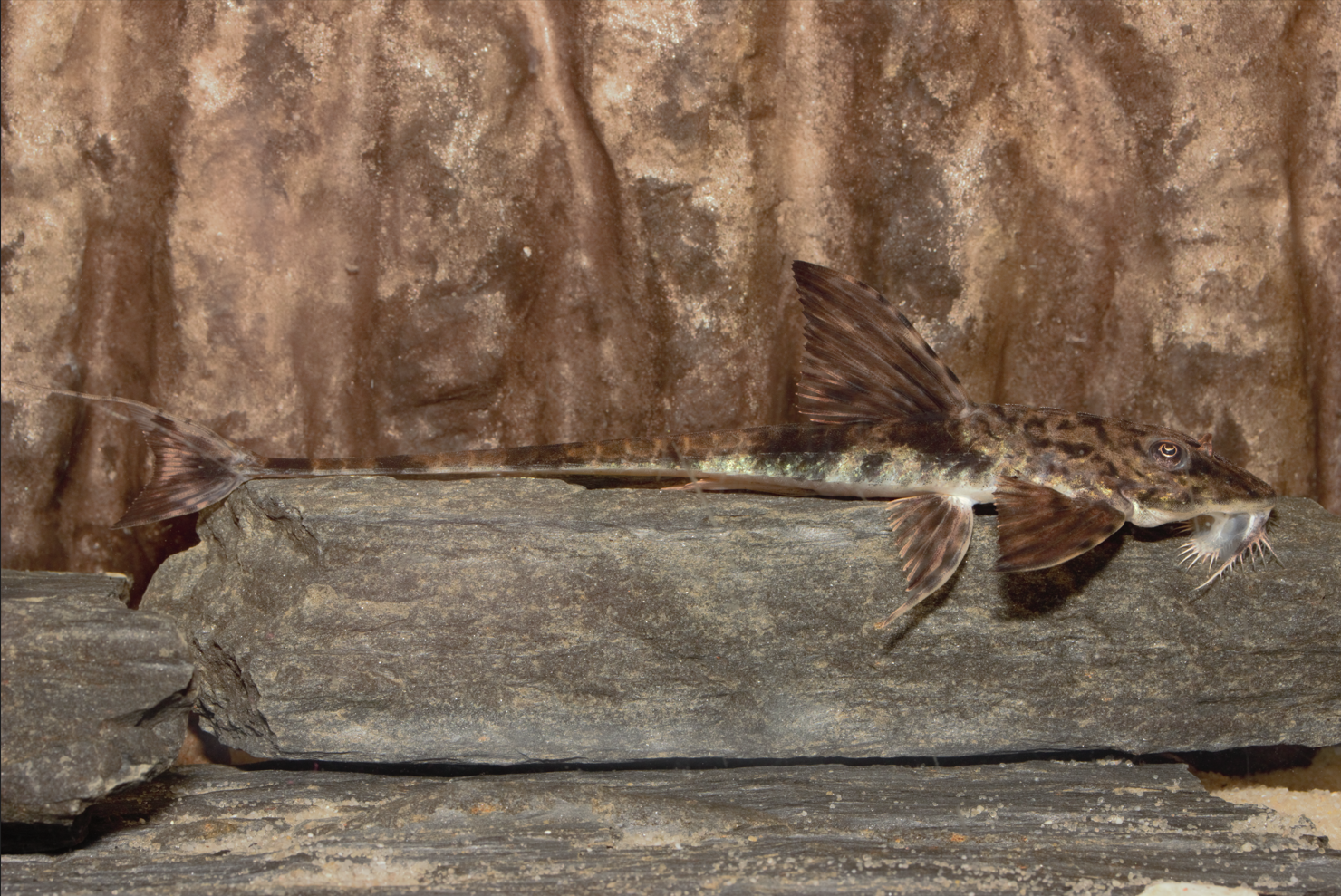


**Supplementary Figure 14. Habitus images of *Loricaria simillima*.**


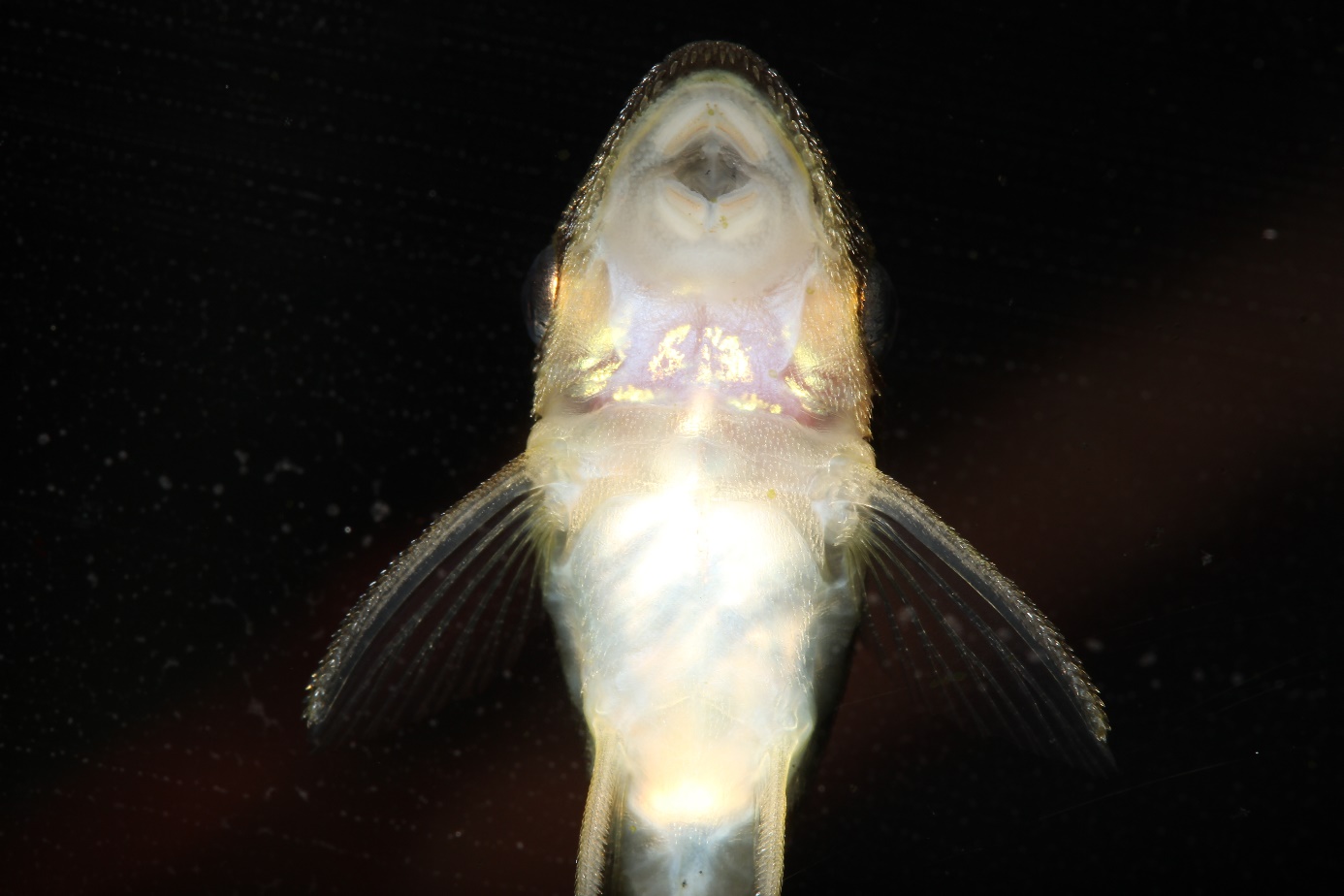

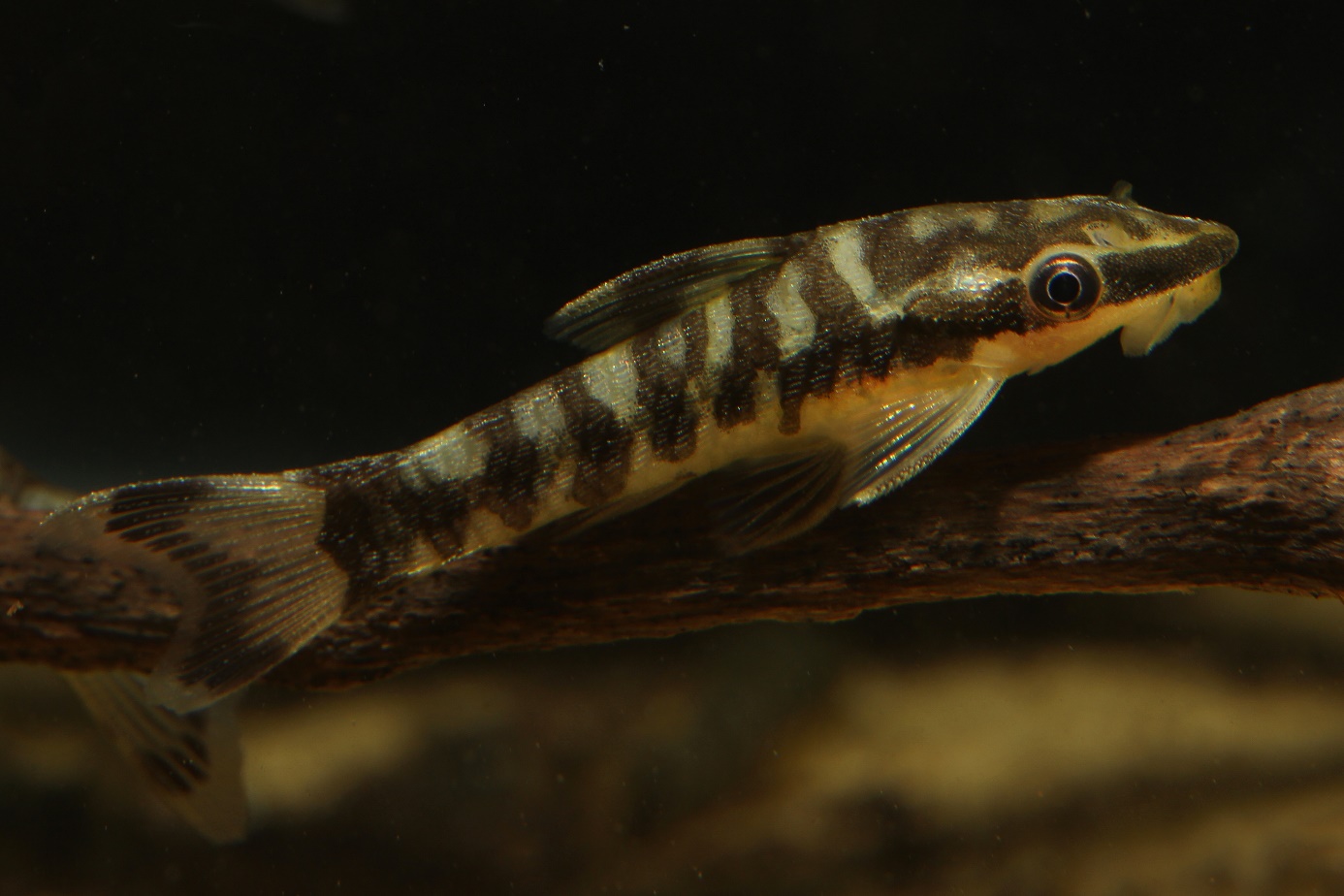


**Supplementary Figure 15. Habitus images of *Otocinclus cocama*.**


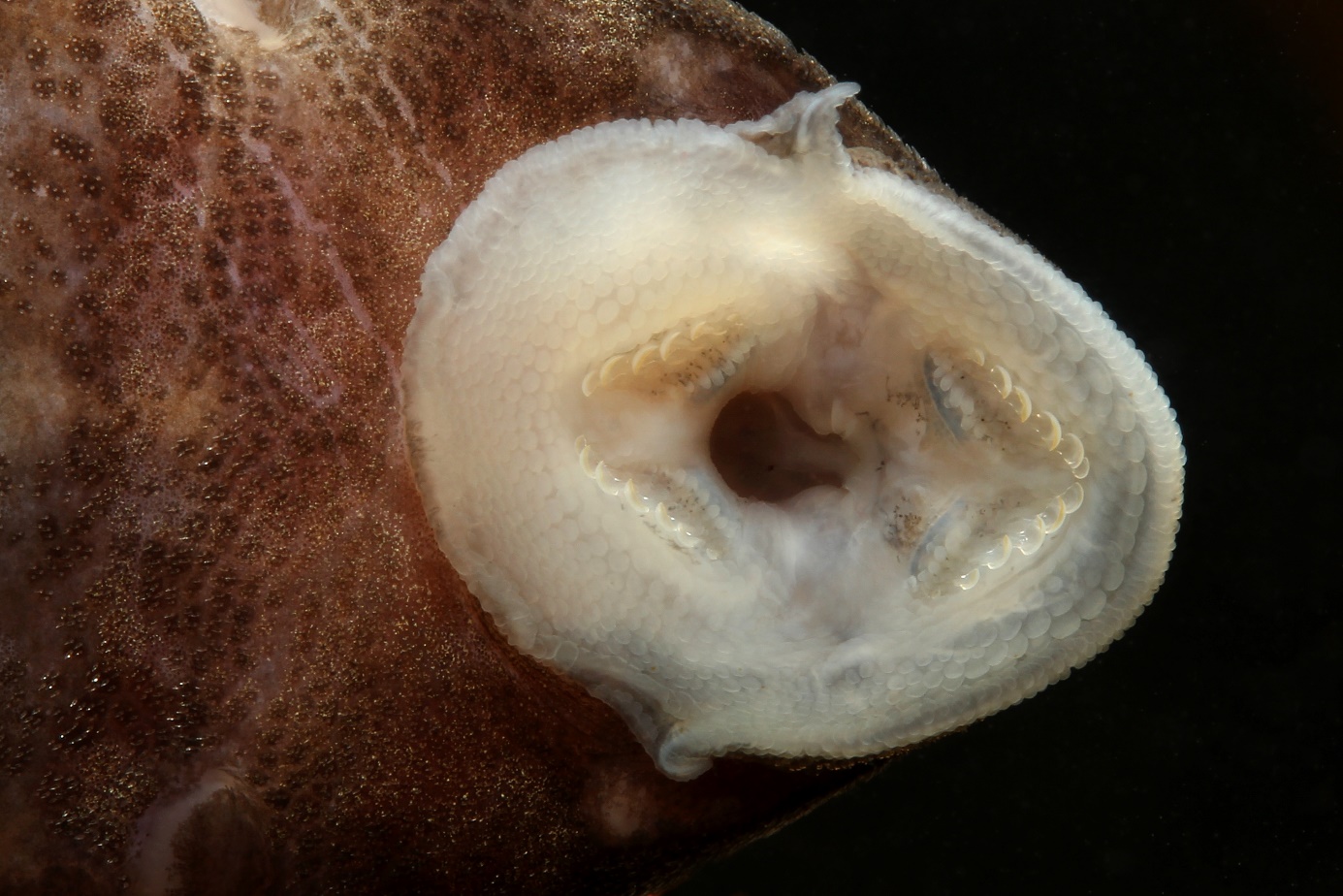

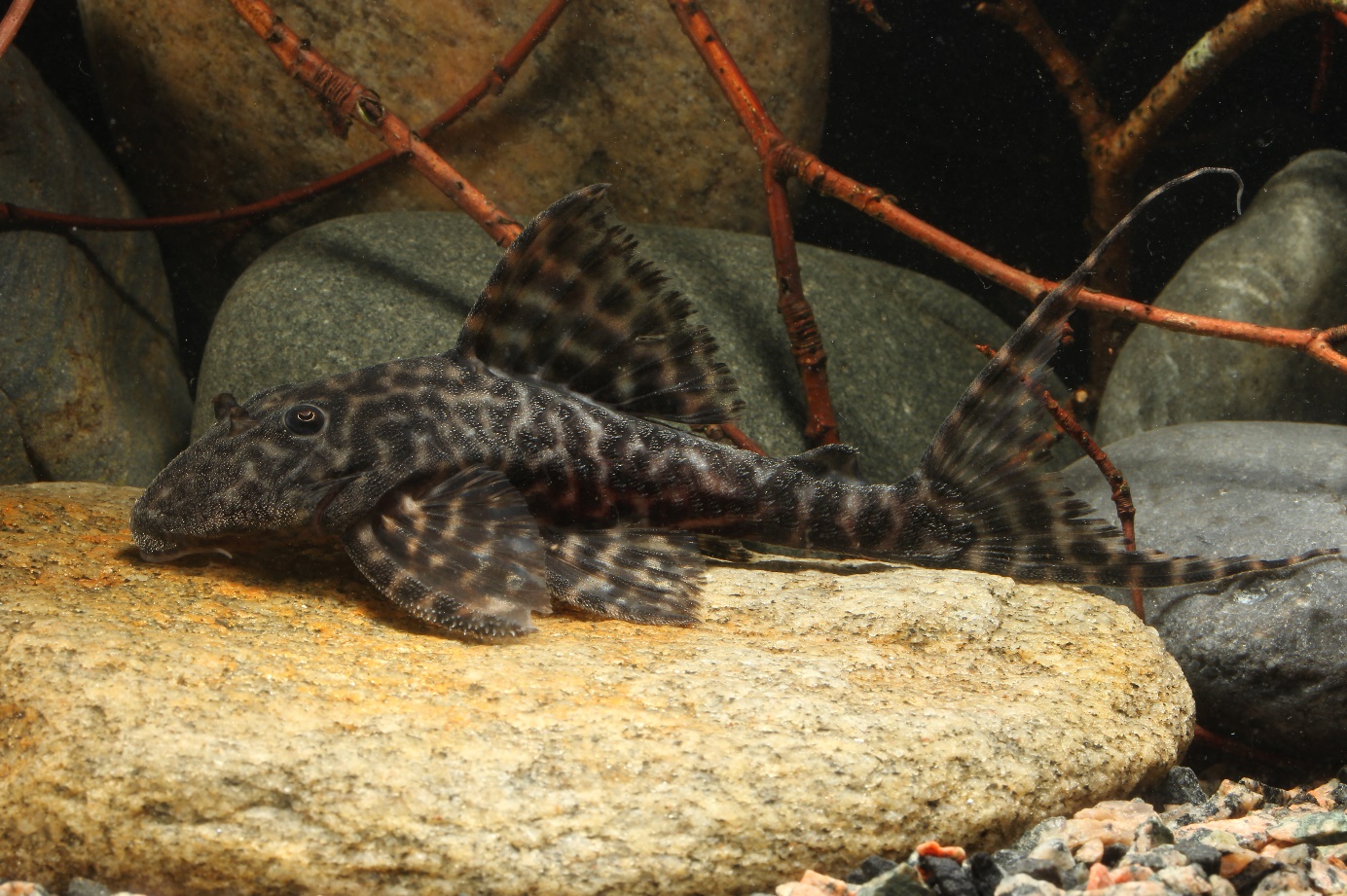


**Supplementary Figure 16. Habitus images of *Panaqolus* sp. L351.**


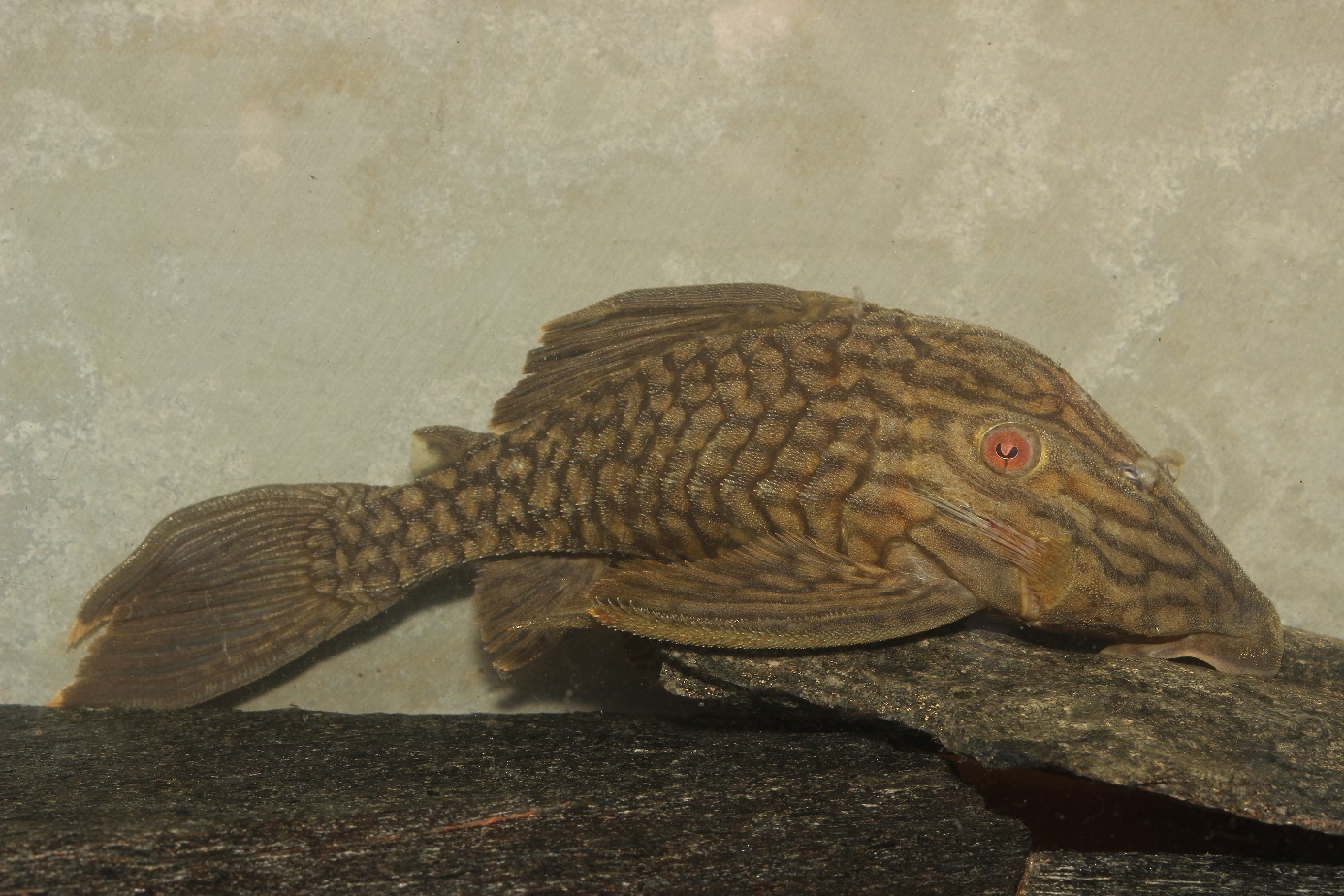

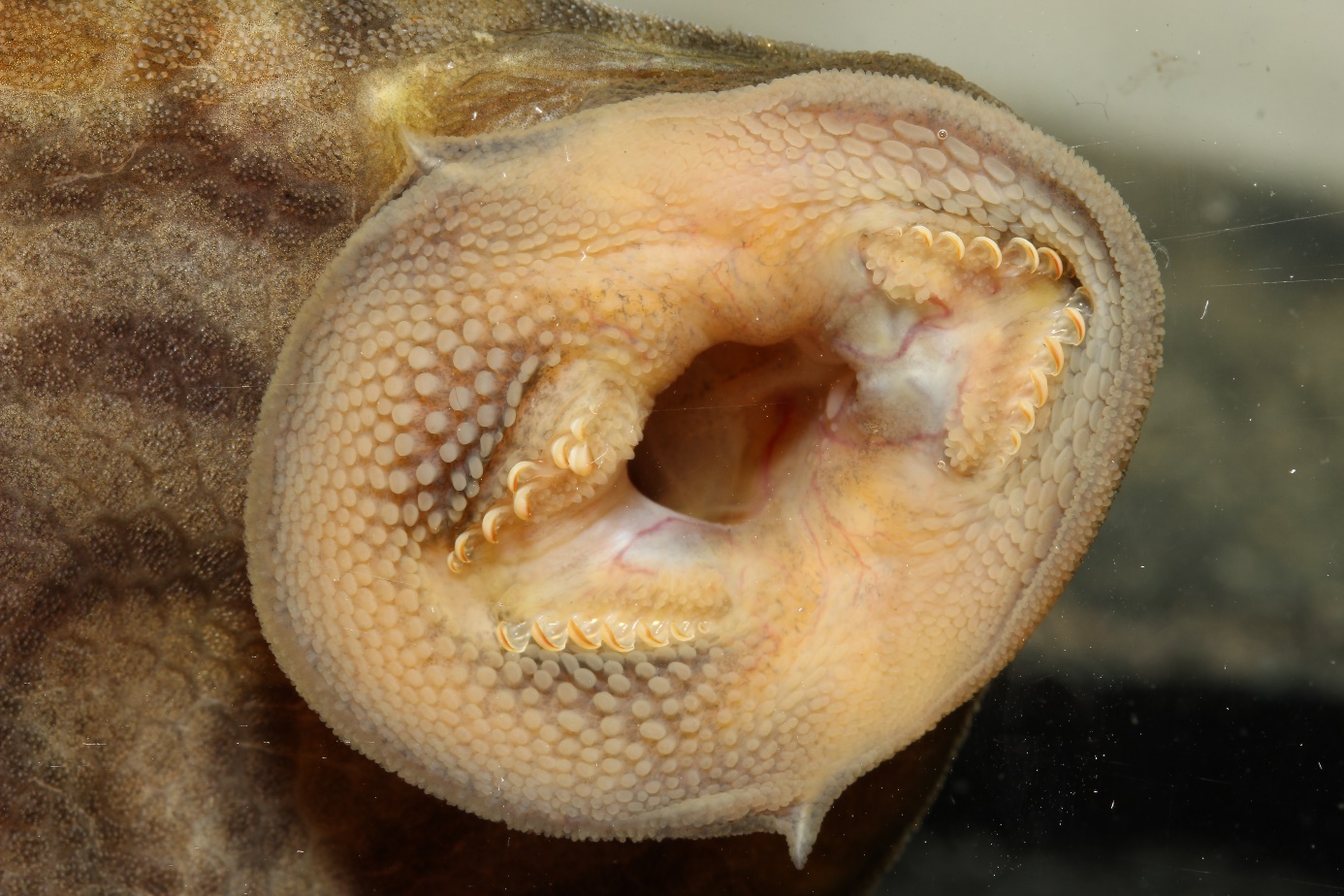


**Supplementary Figure 17. Habitus images of *Panaque nigrolineatus*.**


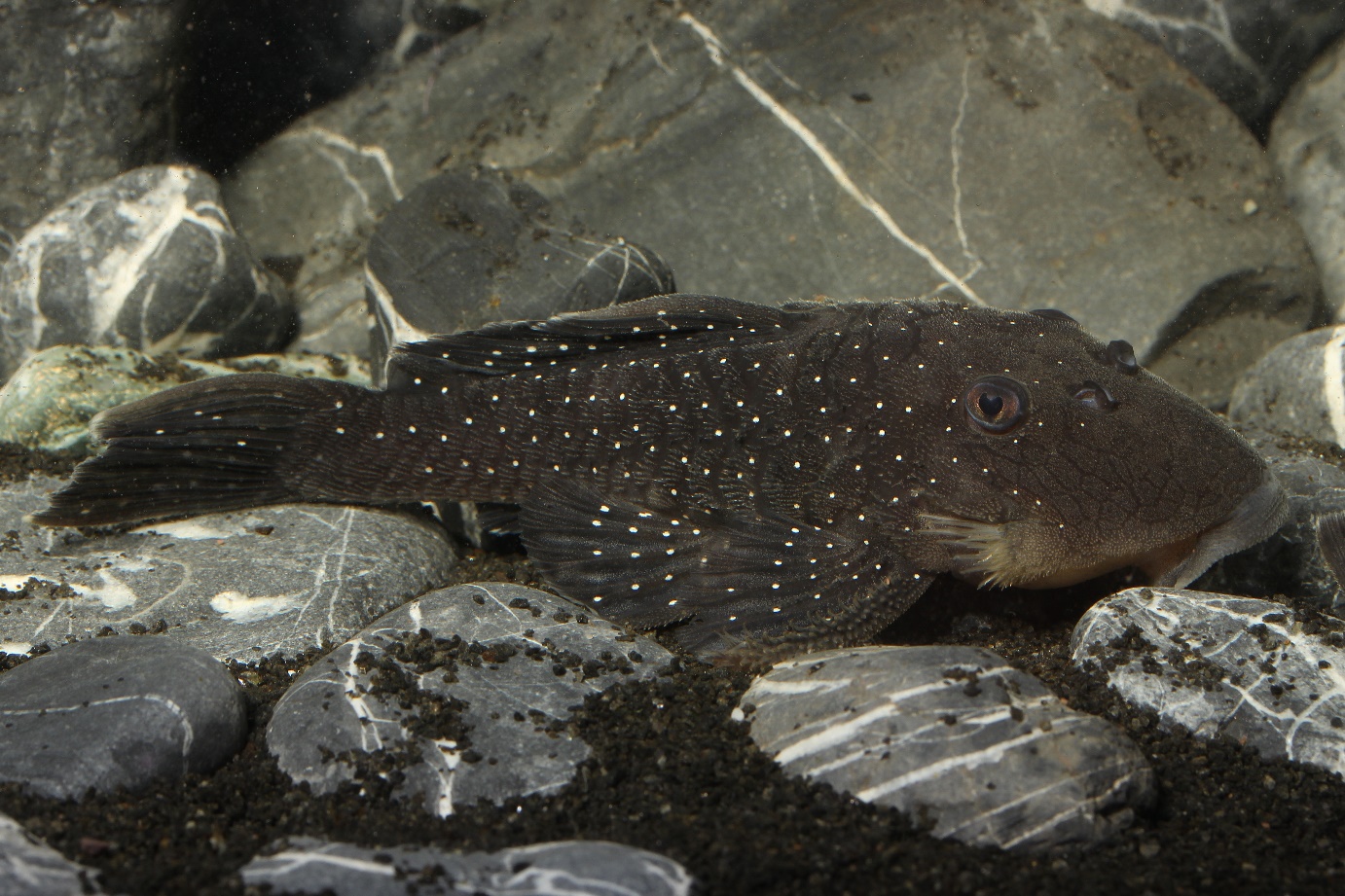

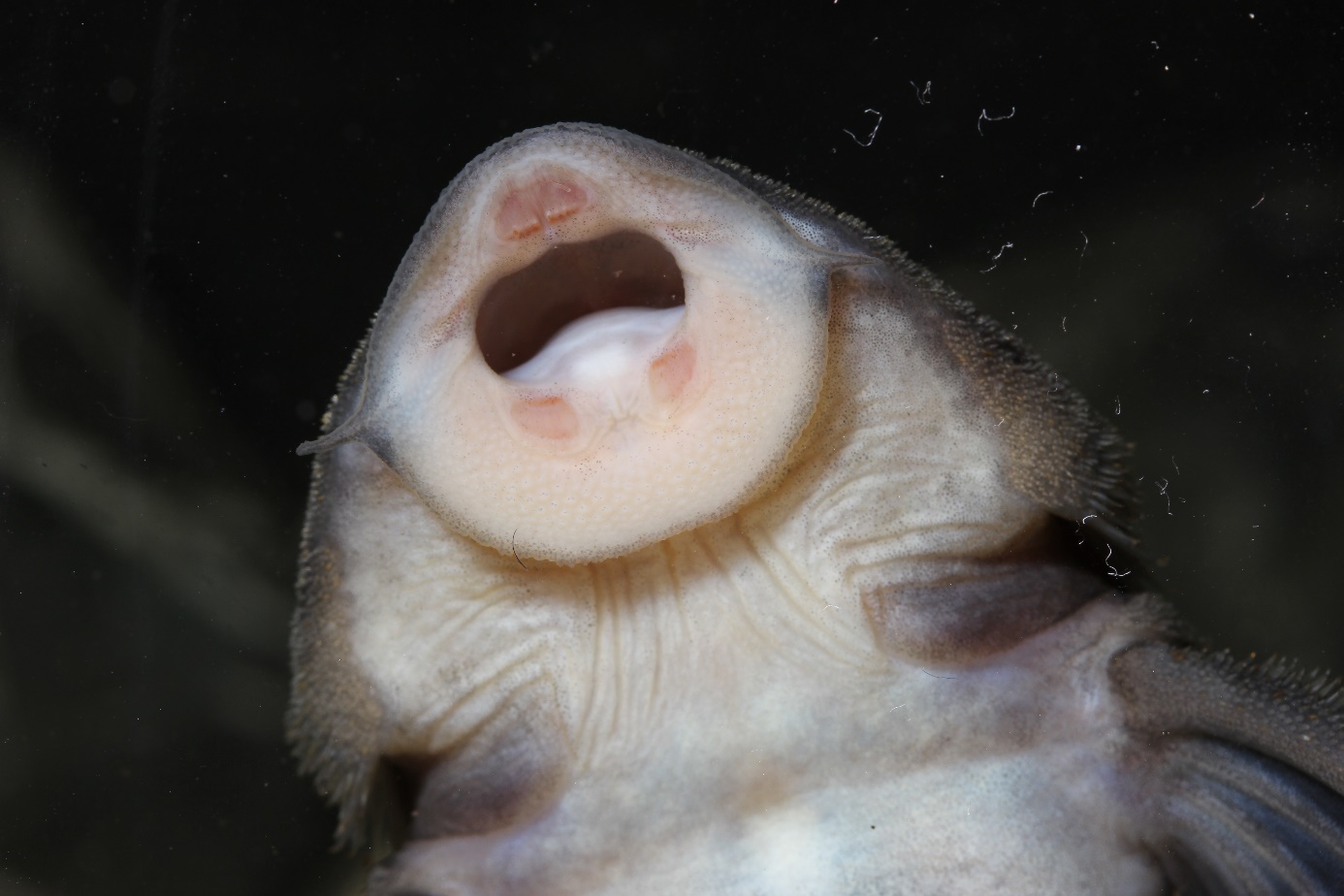


**Supplementary Figure 18. Habitus images of *Parancistrus nudiventris*.**


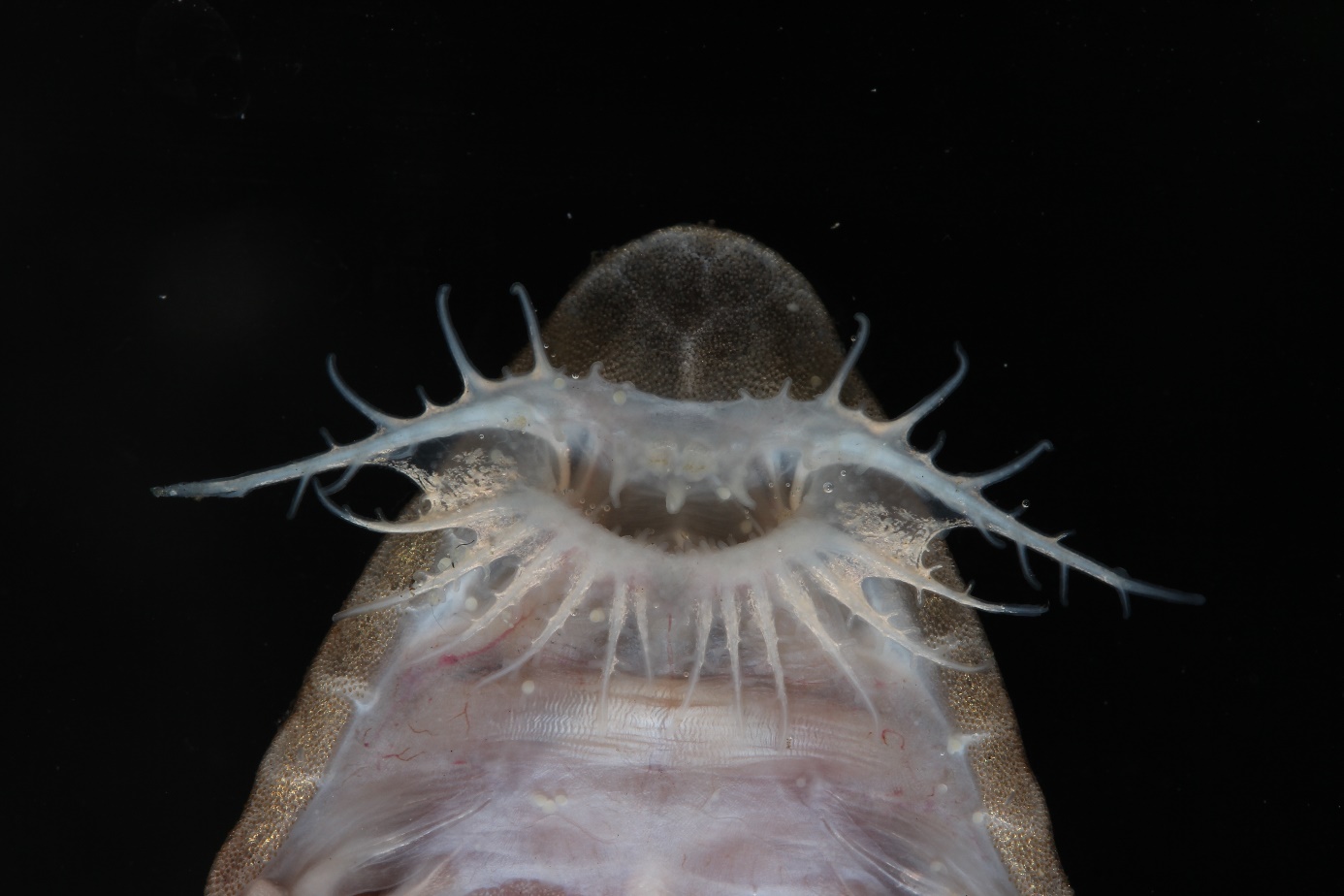

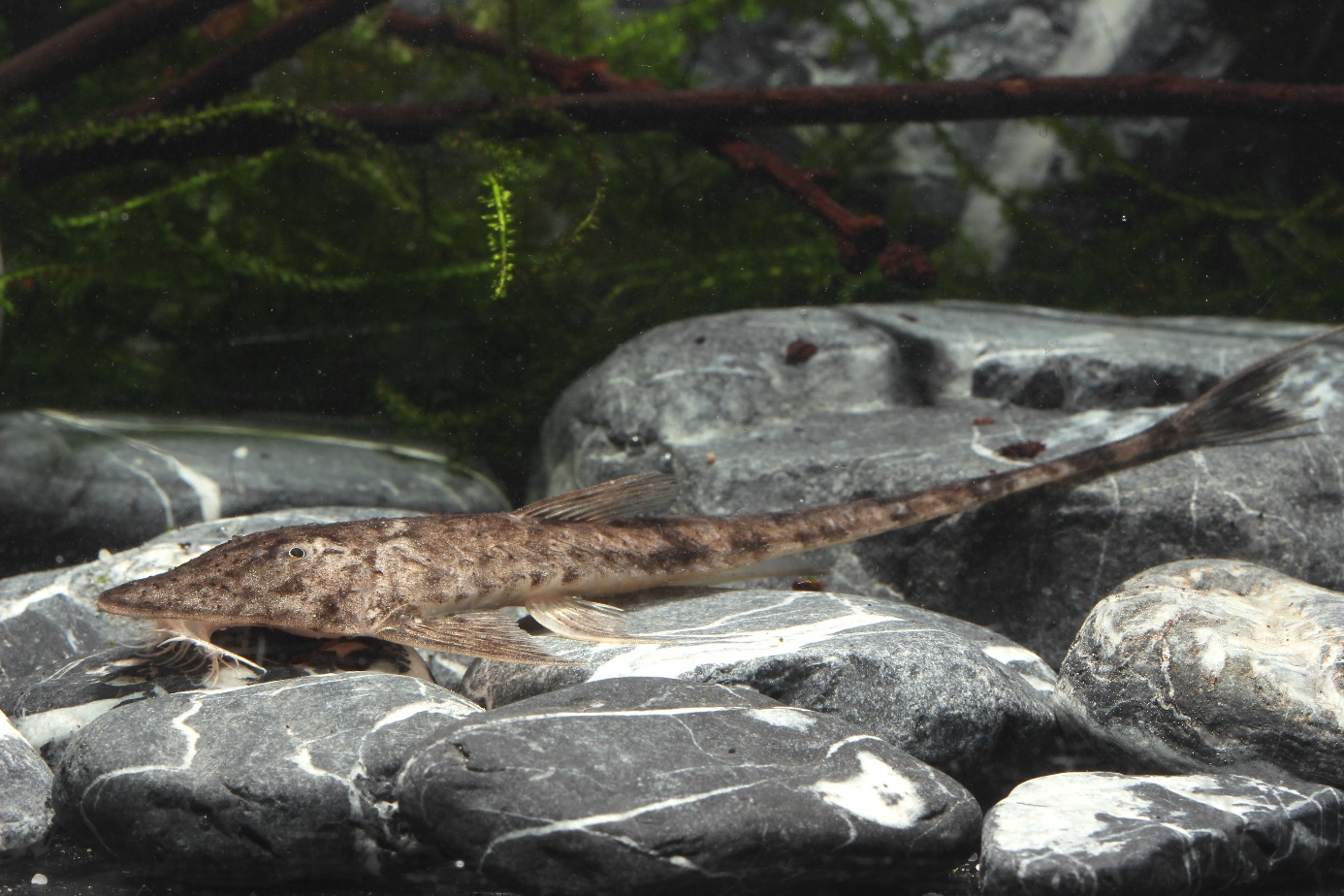


**Supplementary Figure 19. Habitus images of *Pseudohemiodon almendarizi*.**


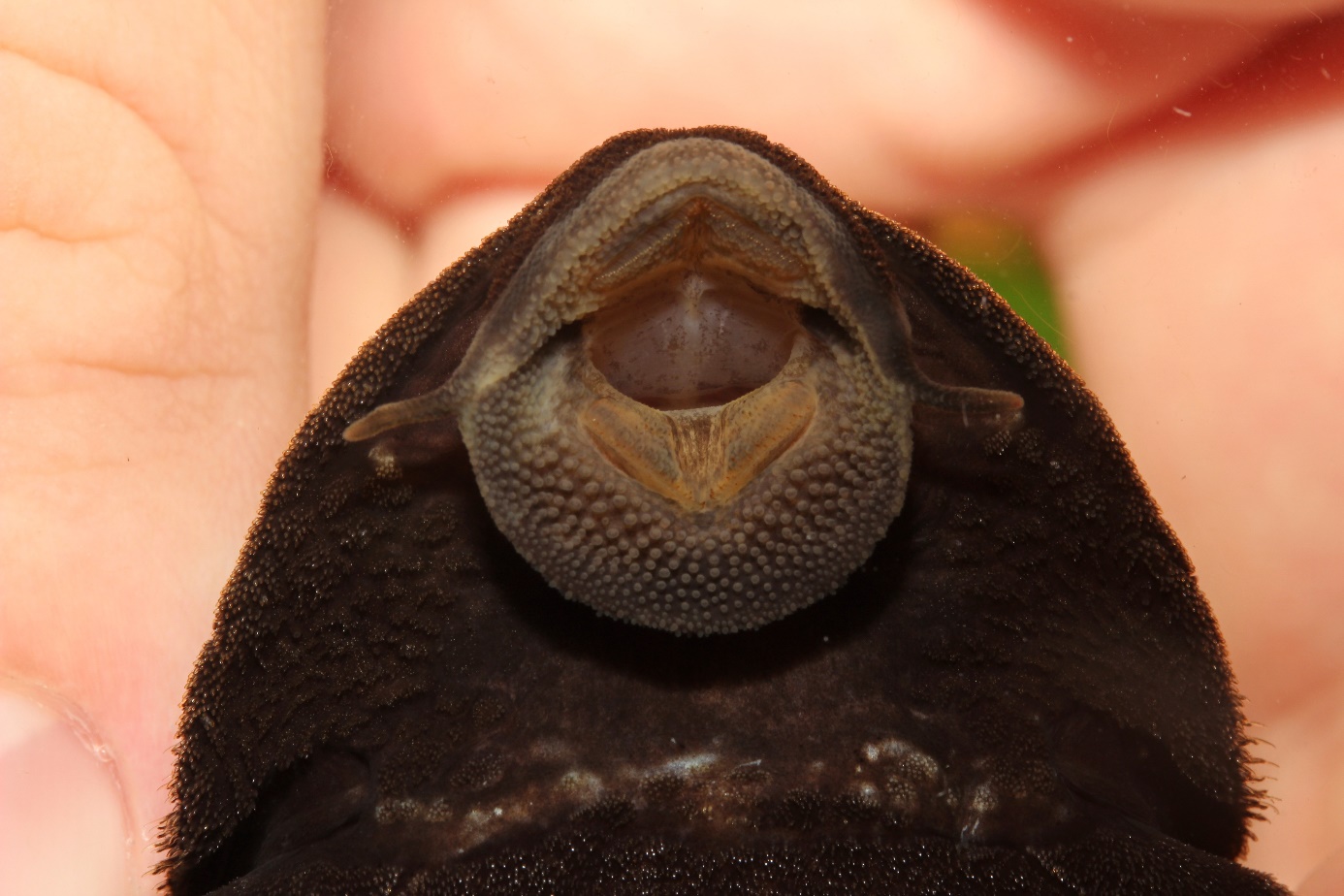

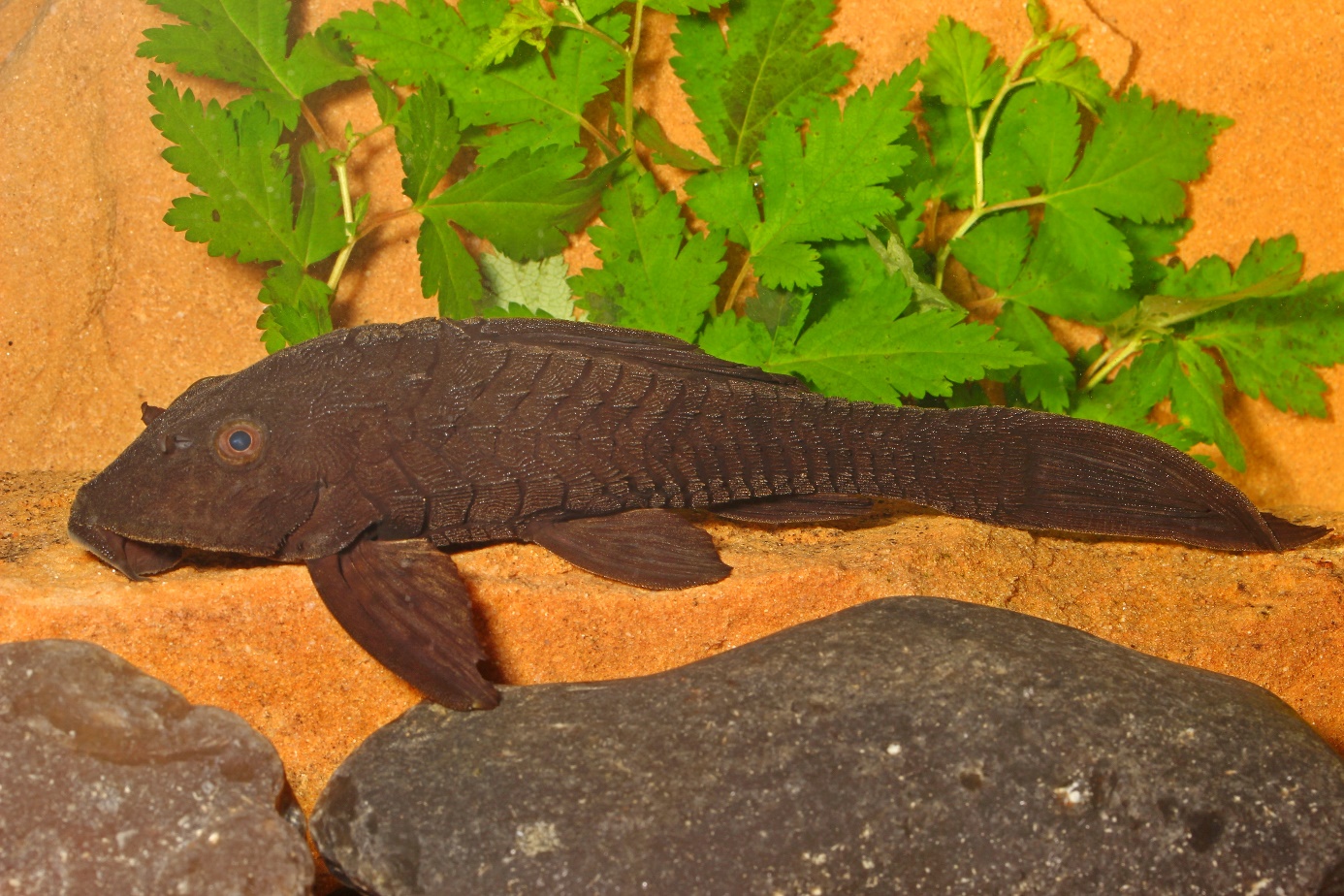


**Supplementary Figure 20. Habitus images of *Pseudorinelepis genibarbis*.**


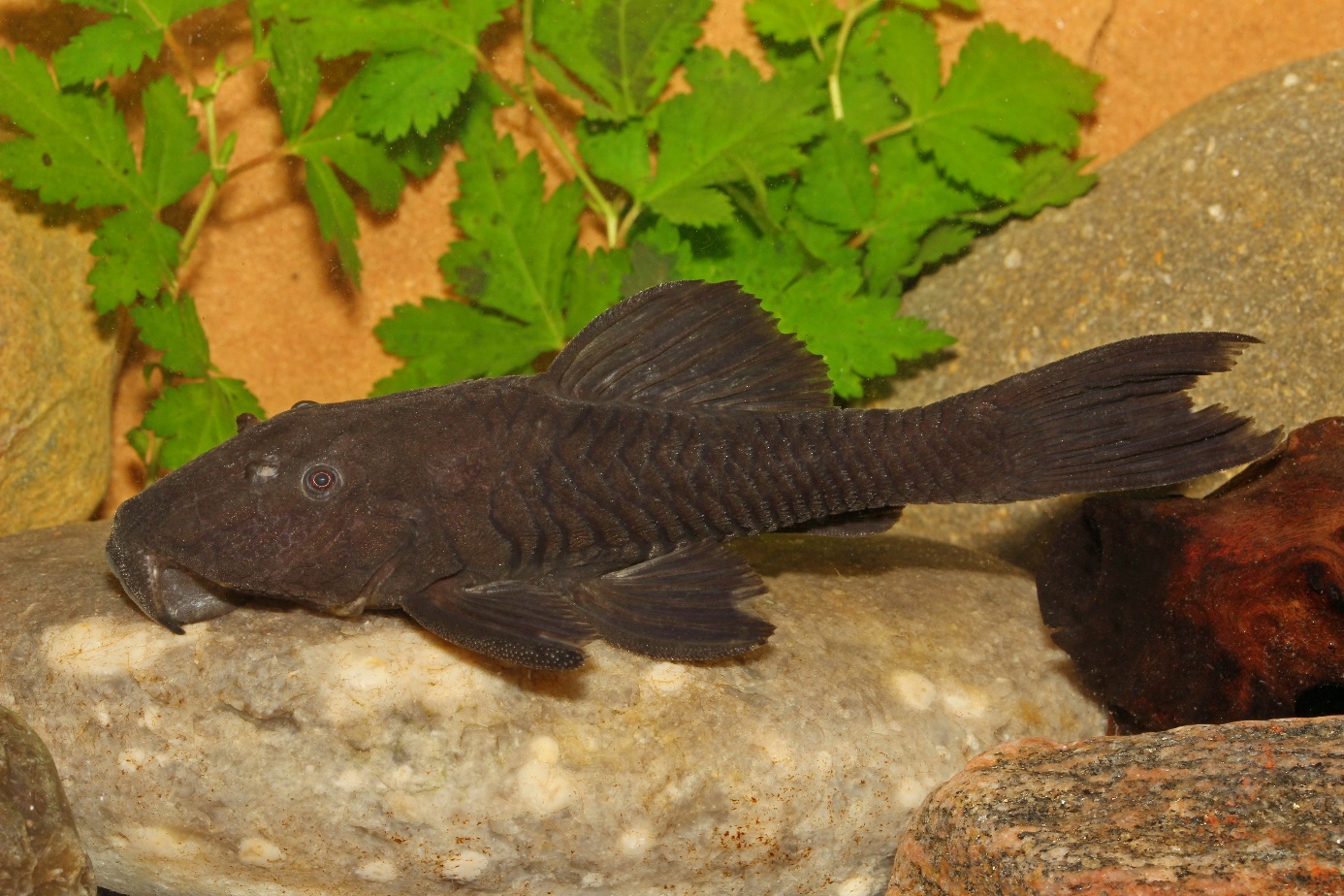

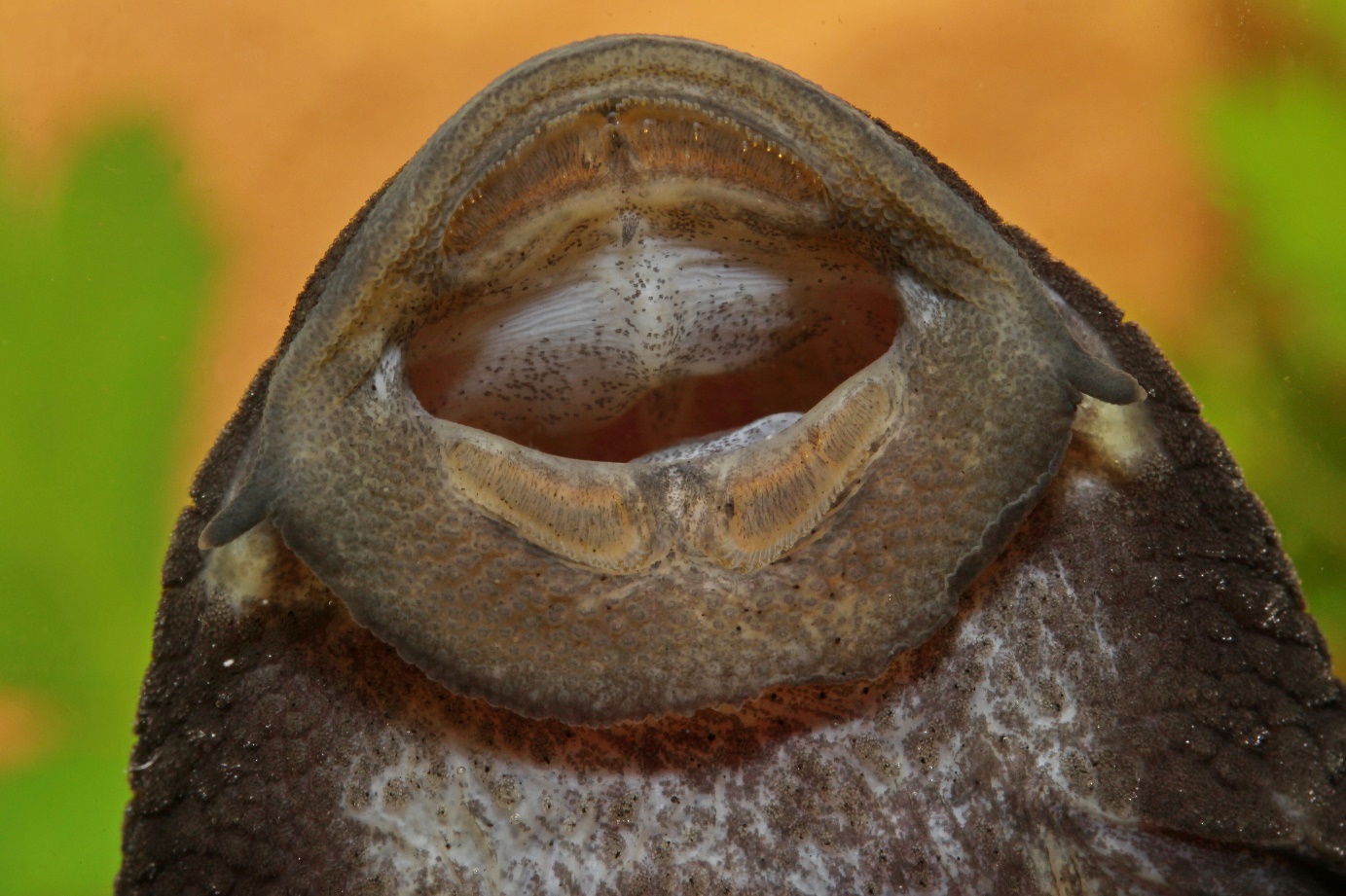


**Supplementary Figure 21. Habitus images of *Rhinelepis aspera*.**


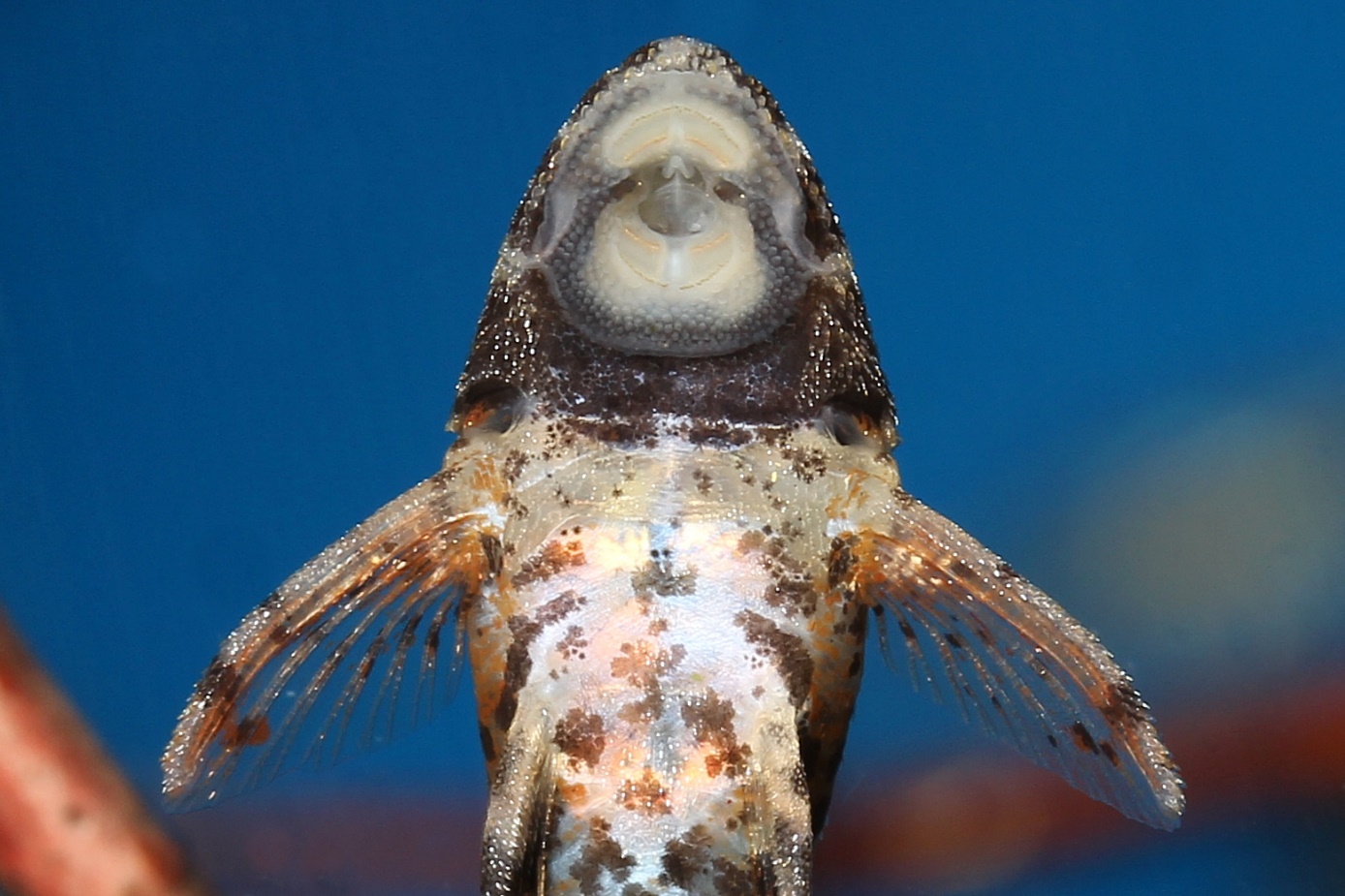

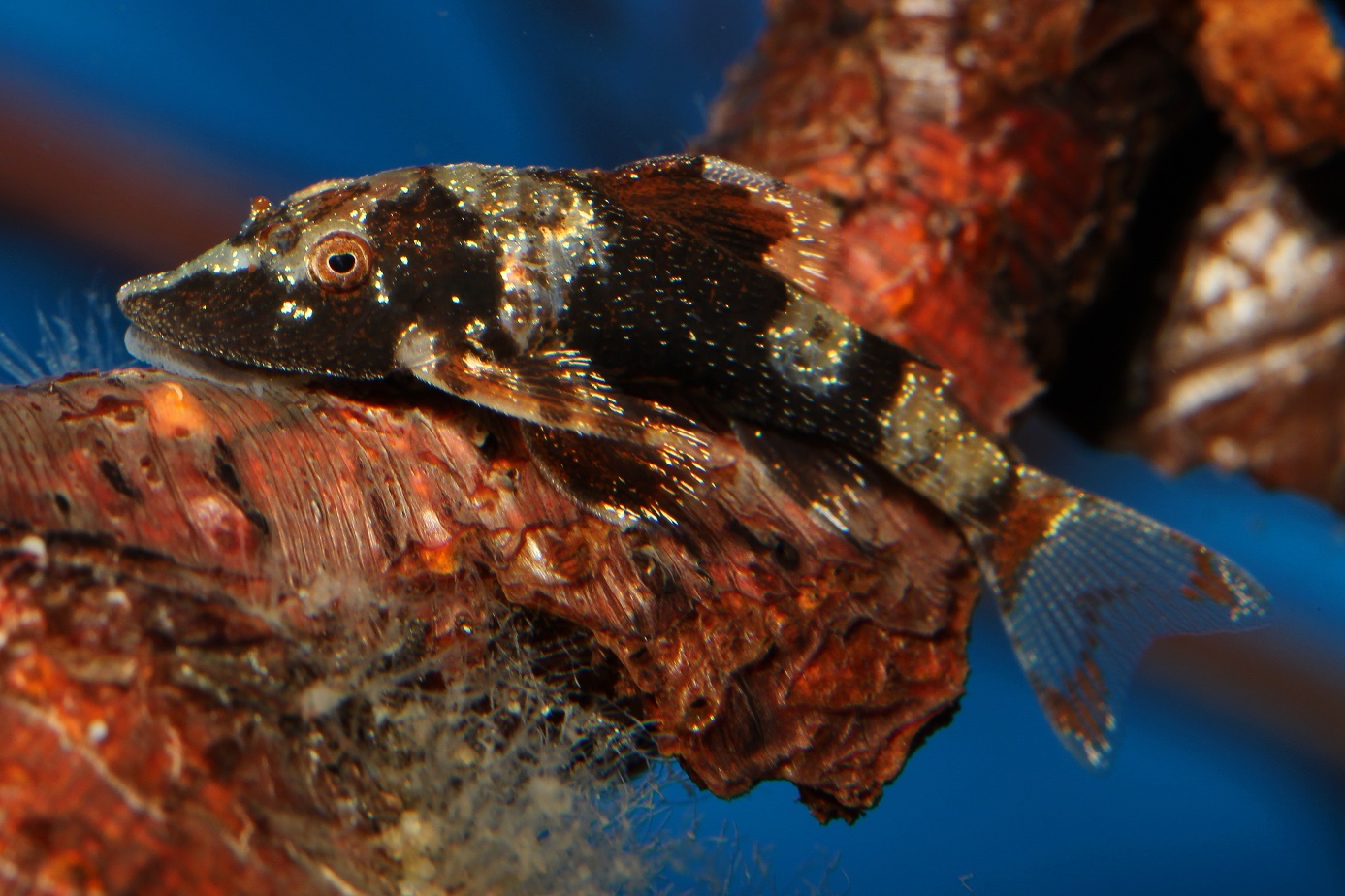


**Supplementary Figure 22. Habitus images of *Rhinotocinclus isabelae*.**


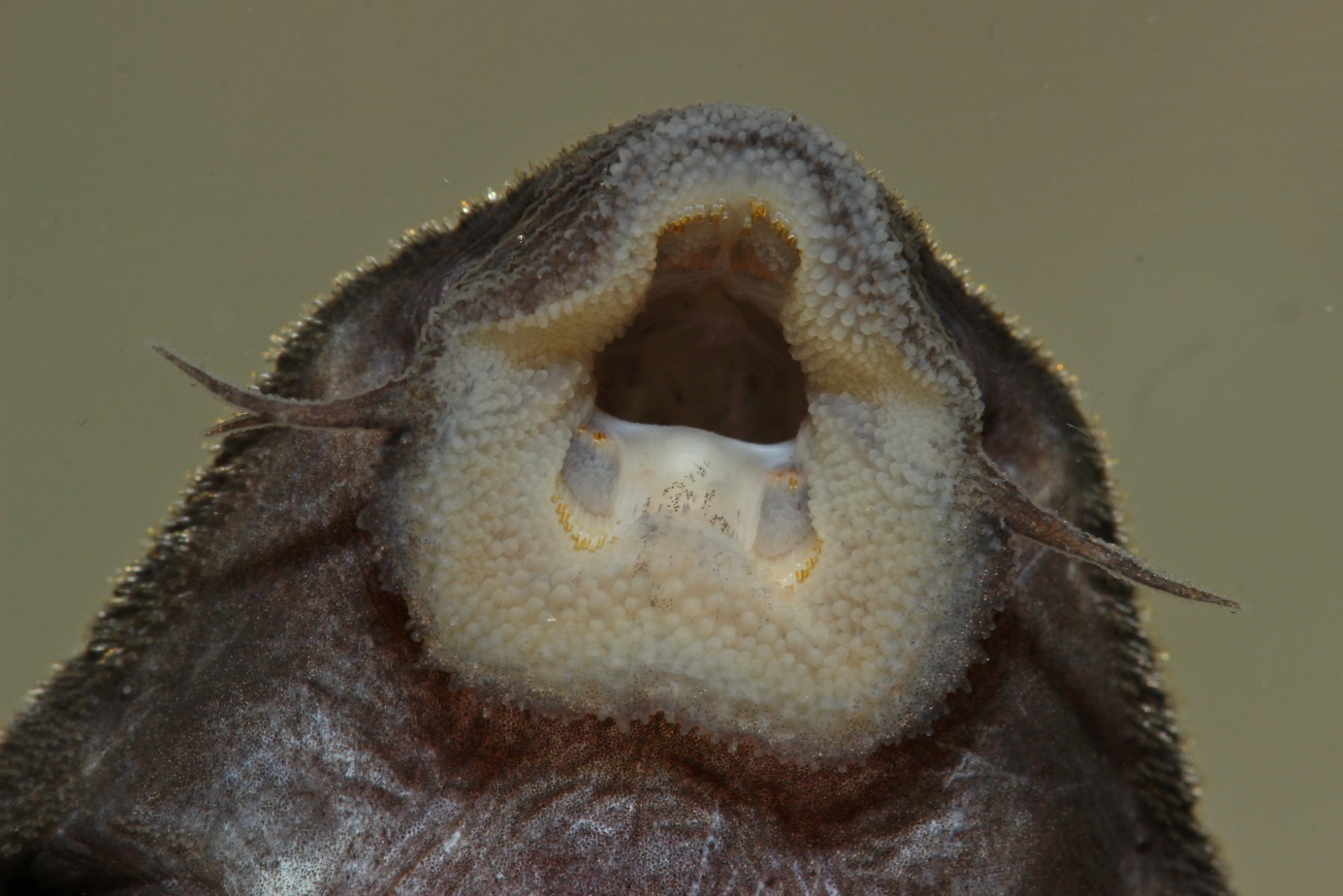

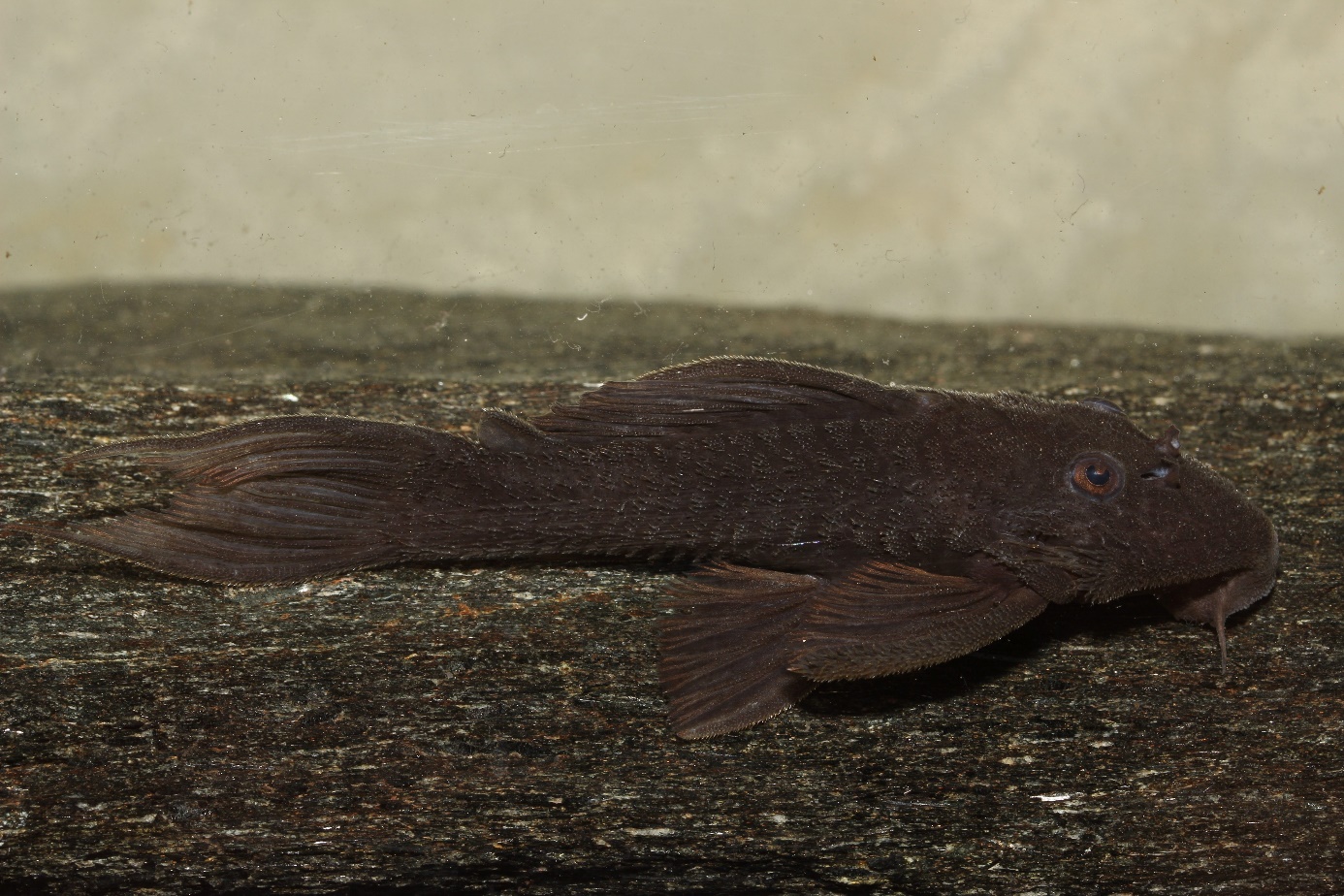


**Supplementary Figure 23. Habitus images of *Spectracanthicus immaculatus*.**
